# Supplementary figures and images for: Multi-Pathway Mechanisms of Engeletin in Ischemic Stroke: A Comprehensive Study Based on Network Pharmacology, Machine Learning, and Immune Infiltration Analysis
Source: Int J Mol Sci. 2025 Nov 26;26(23):11446. doi: 10.3390/ijms262311446 (PMC12692429; doi:10.3390/ijms262311446)

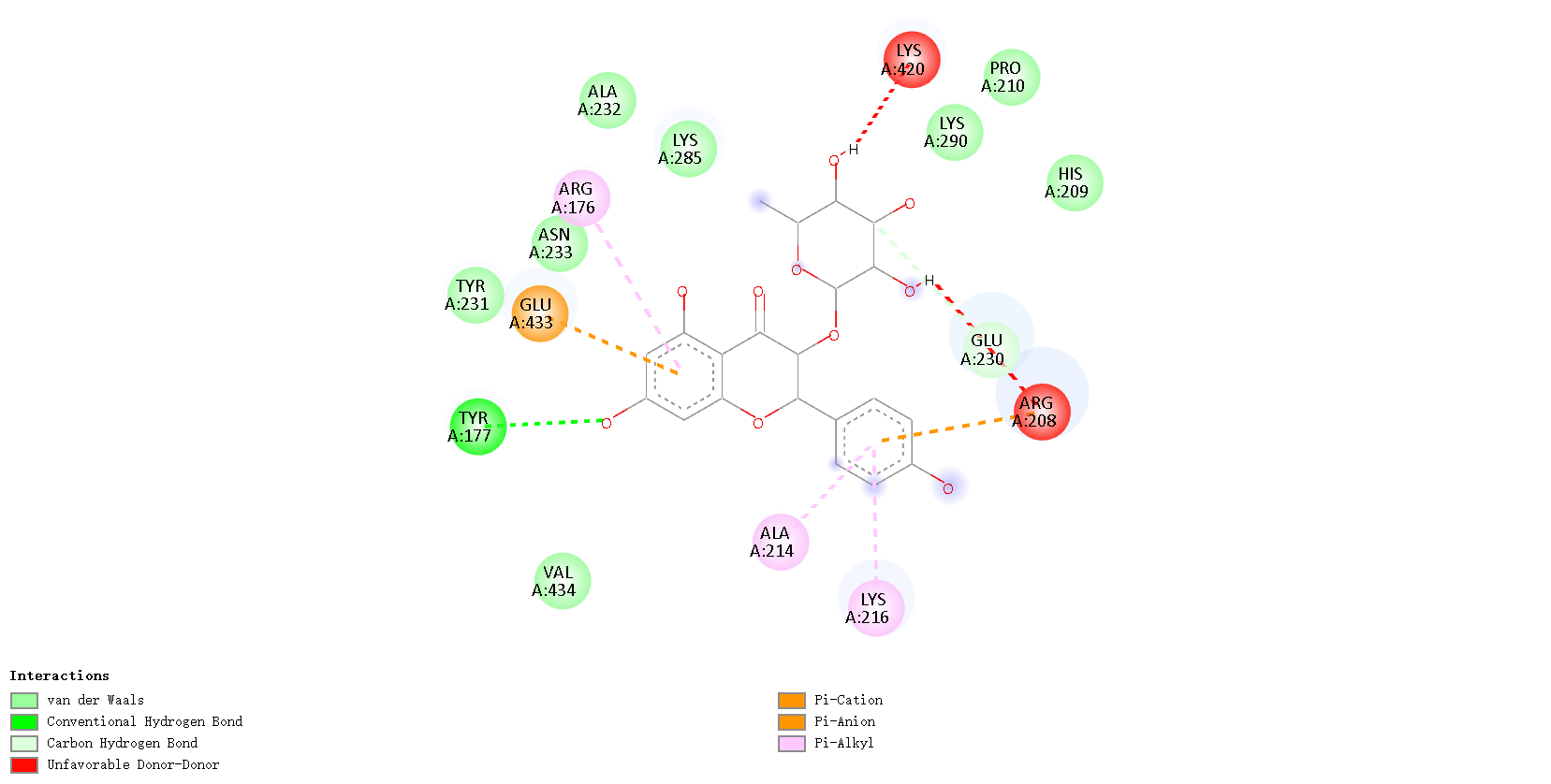

Supplement: Supplementary file 1 [file ijms-26-11446-s001.zip › AKT/2D.png]

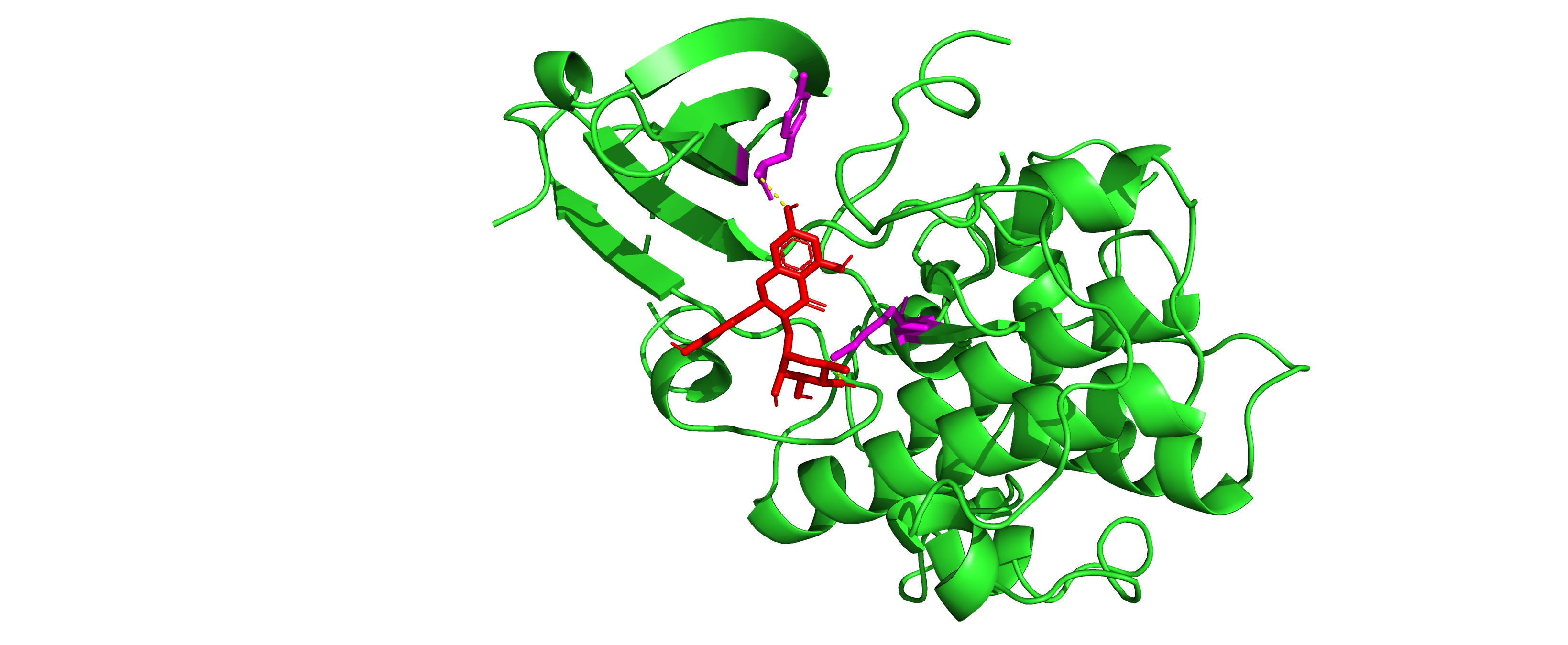

Supplement: Supplementary file 1 [file ijms-26-11446-s001.zip › AKT/big.png]

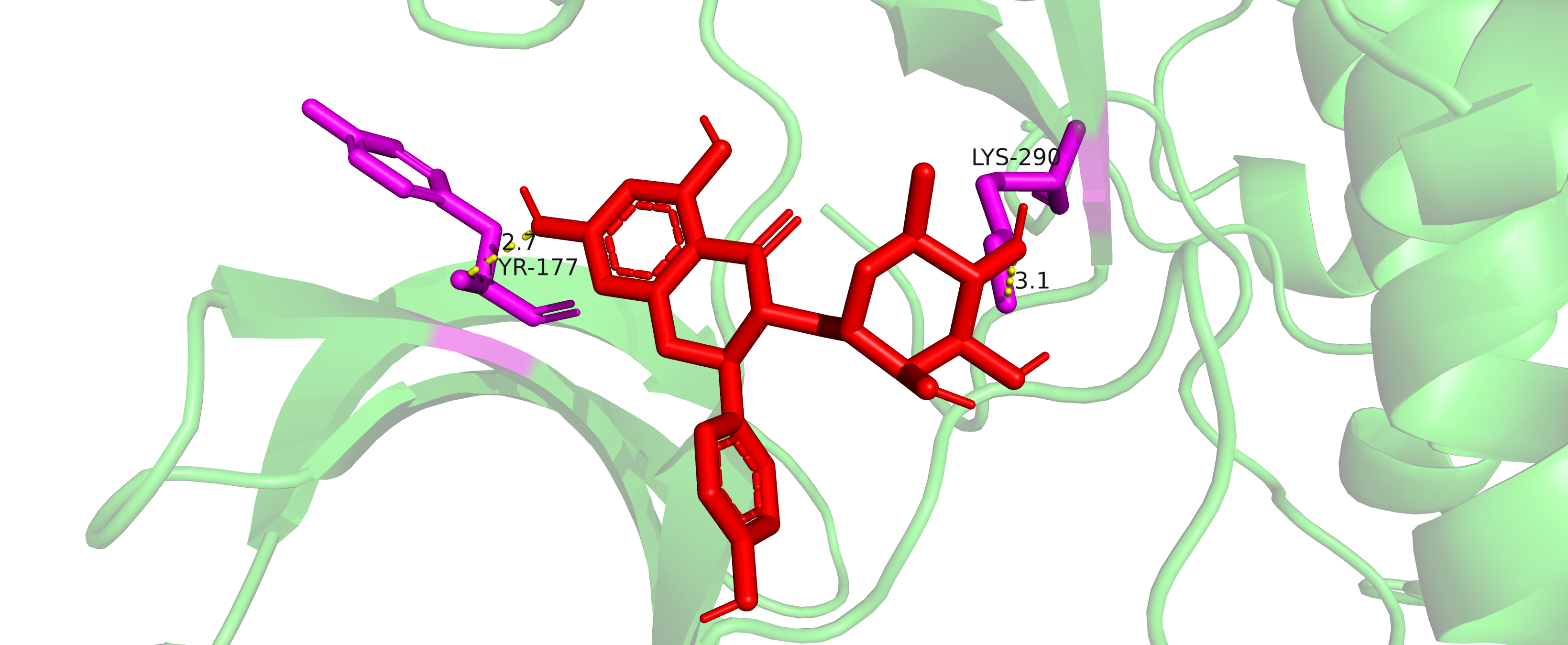

Supplement: Supplementary file 1 [file ijms-26-11446-s001.zip › AKT/smll.png]

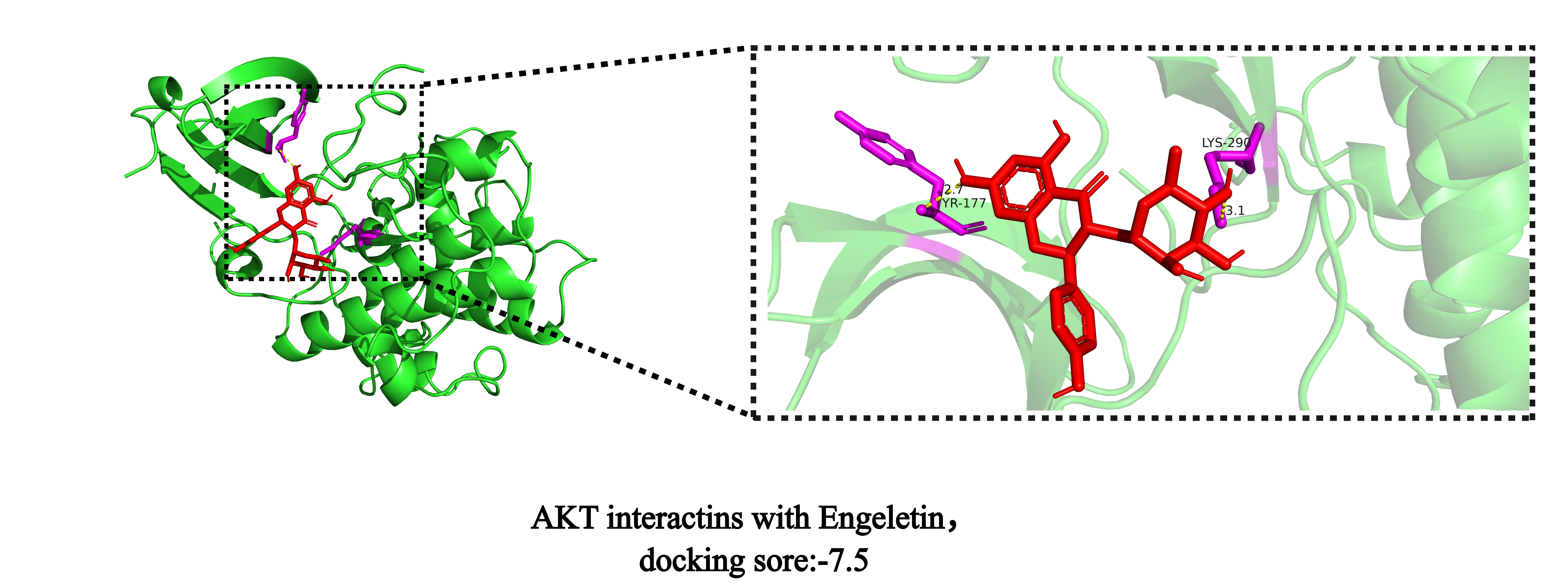

Supplement: Supplementary file 1 [file ijms-26-11446-s001.zip › AKT/combination.png]

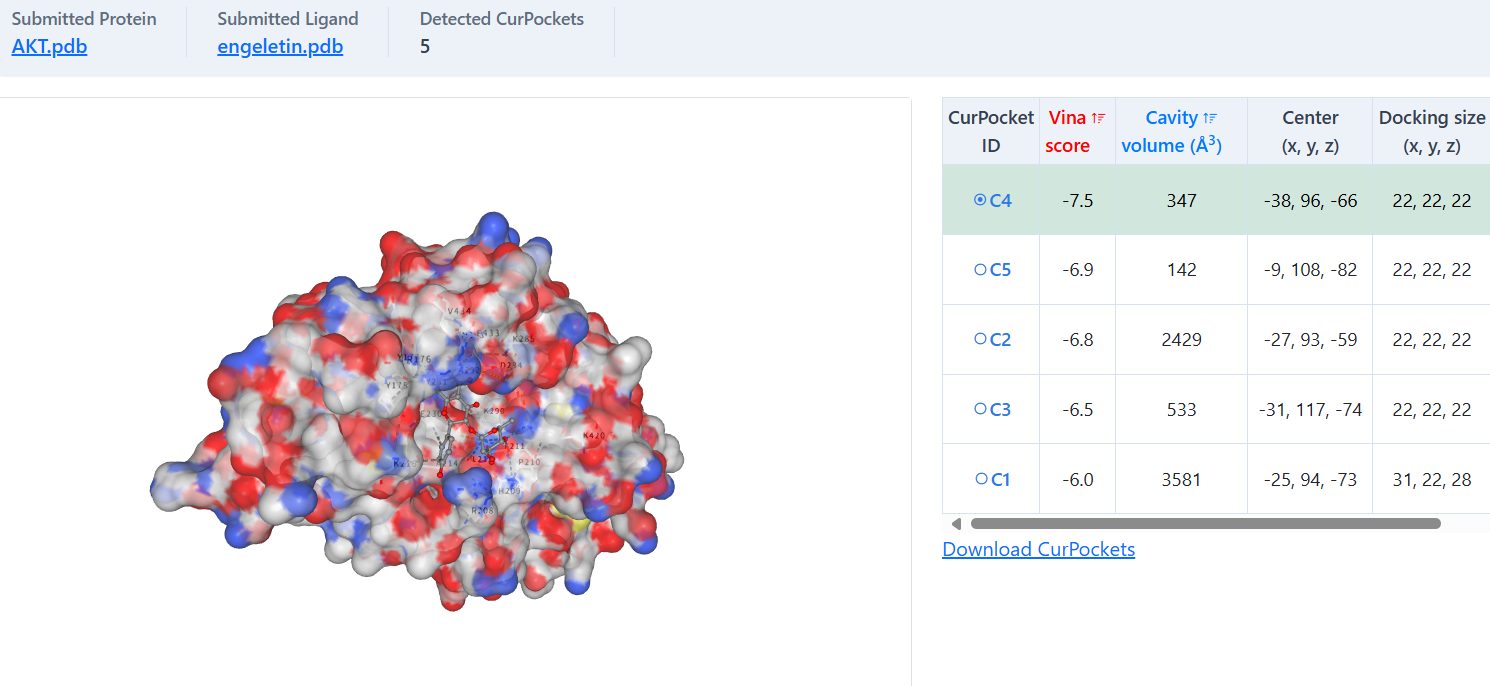

Supplement: Supplementary file 1 [file ijms-26-11446-s001.zip › AKT/free energy.png]

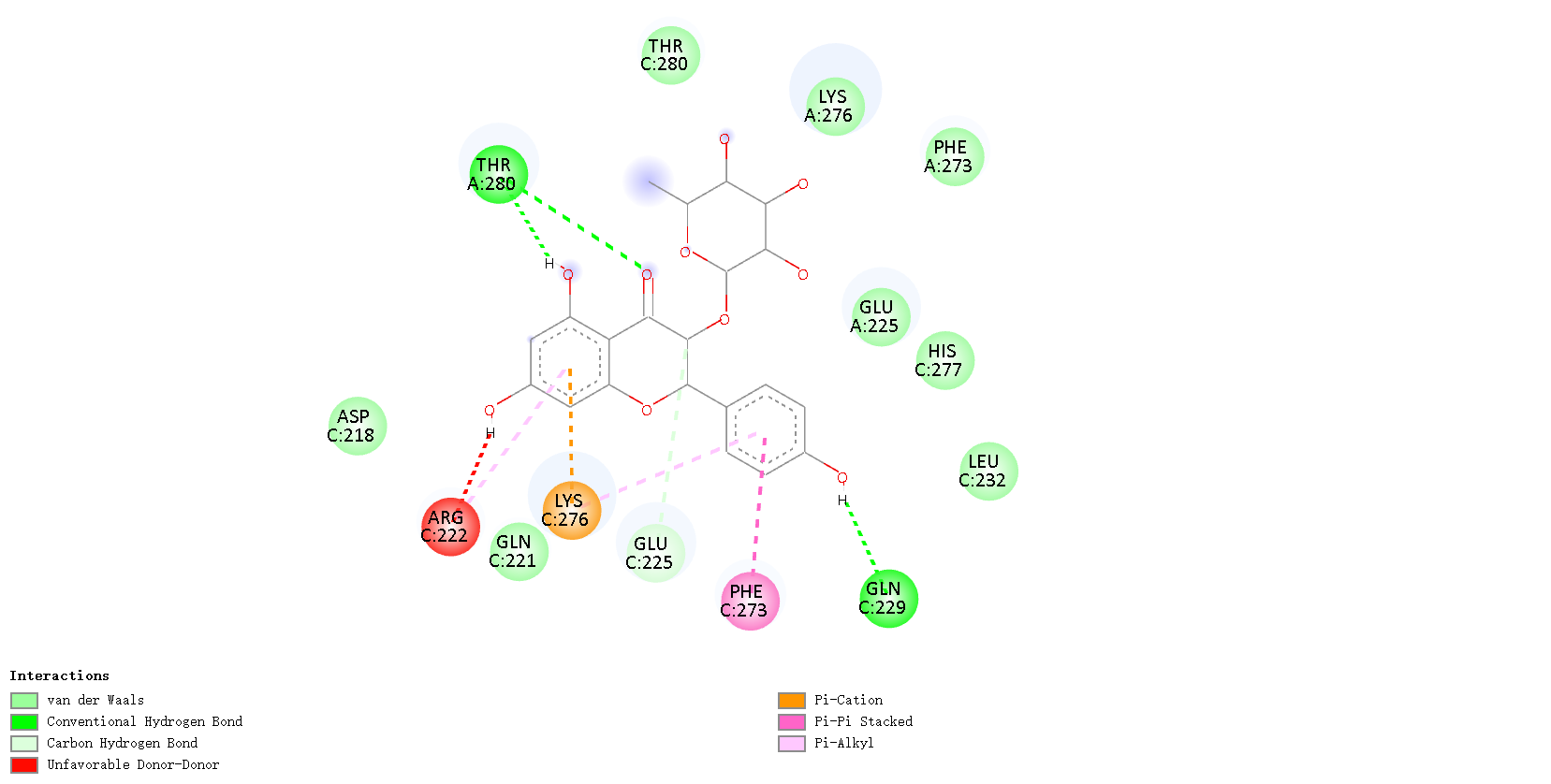

Supplement: Supplementary file 1 [file ijms-26-11446-s001.zip › BAX/2d.png]

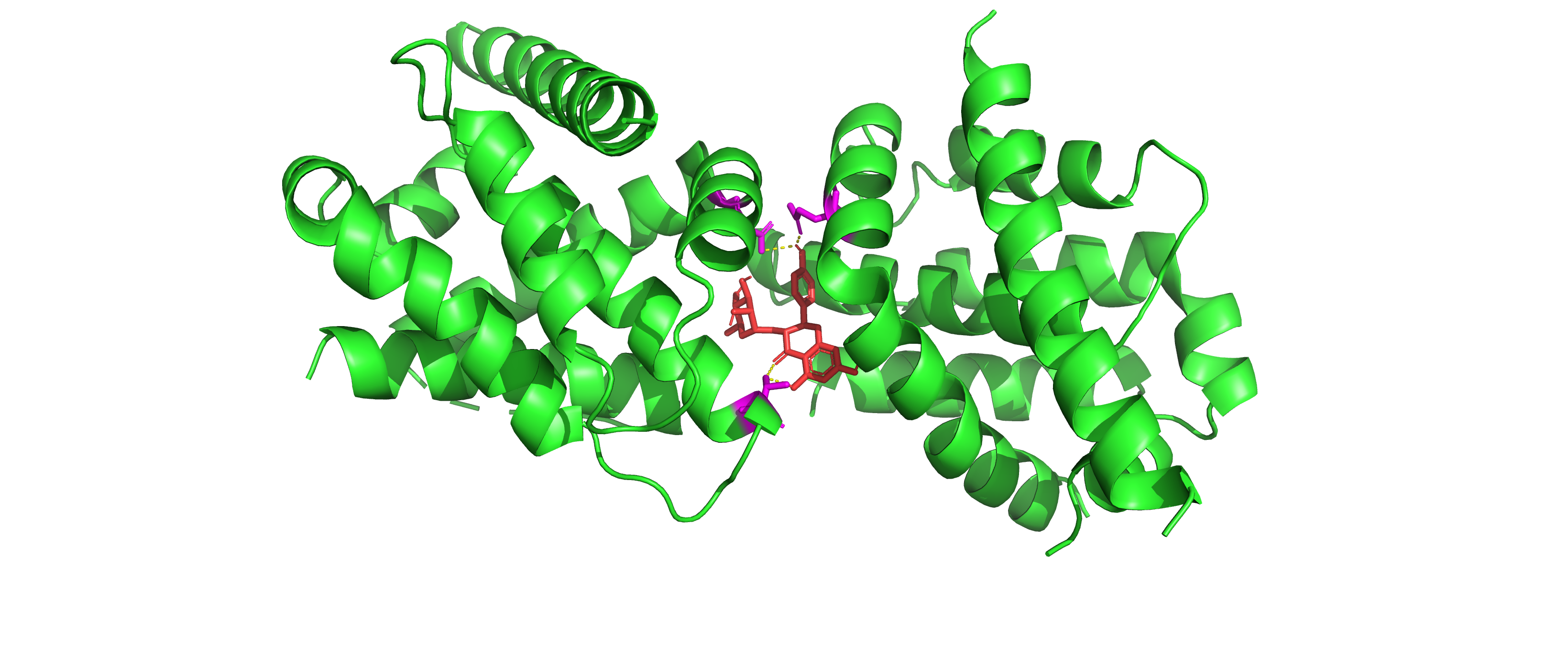

Supplement: Supplementary file 1 [file ijms-26-11446-s001.zip › BAX/big.png]

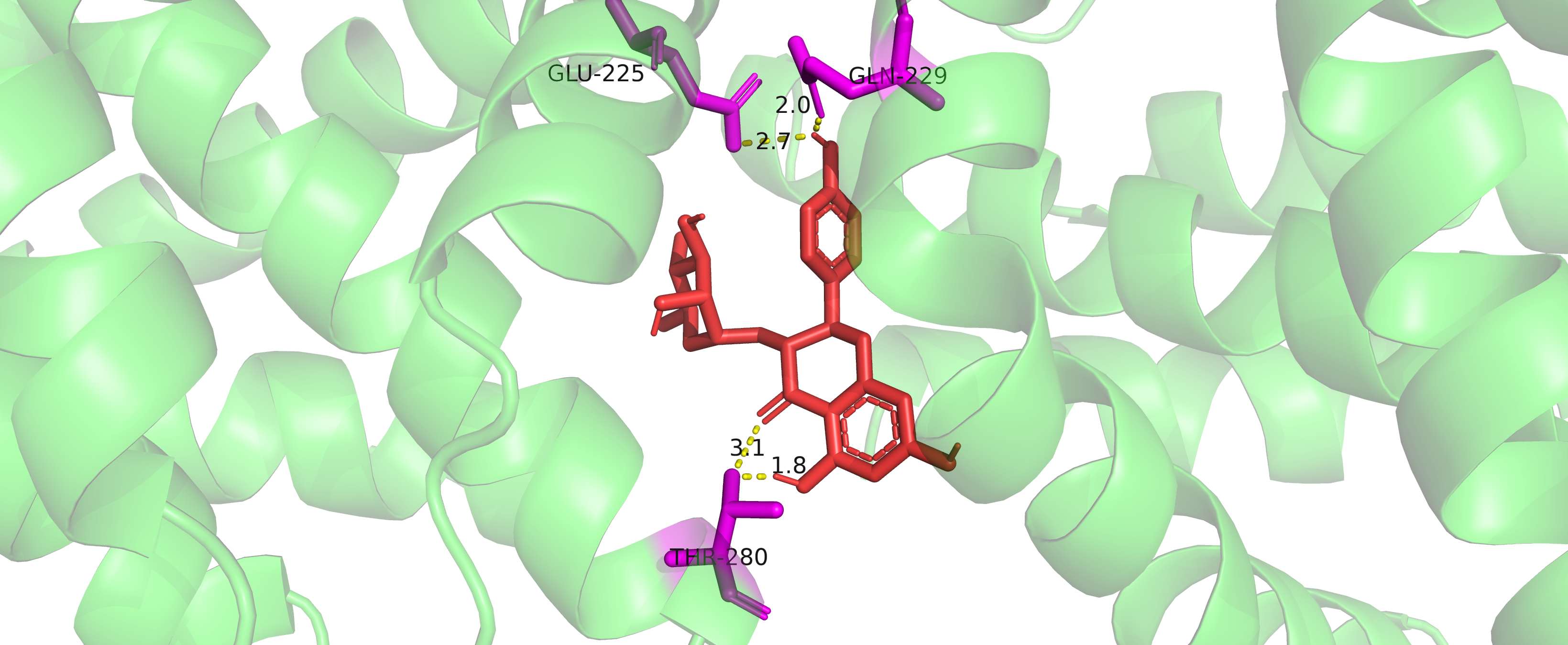

Supplement: Supplementary file 1 [file ijms-26-11446-s001.zip › BAX/small.png]

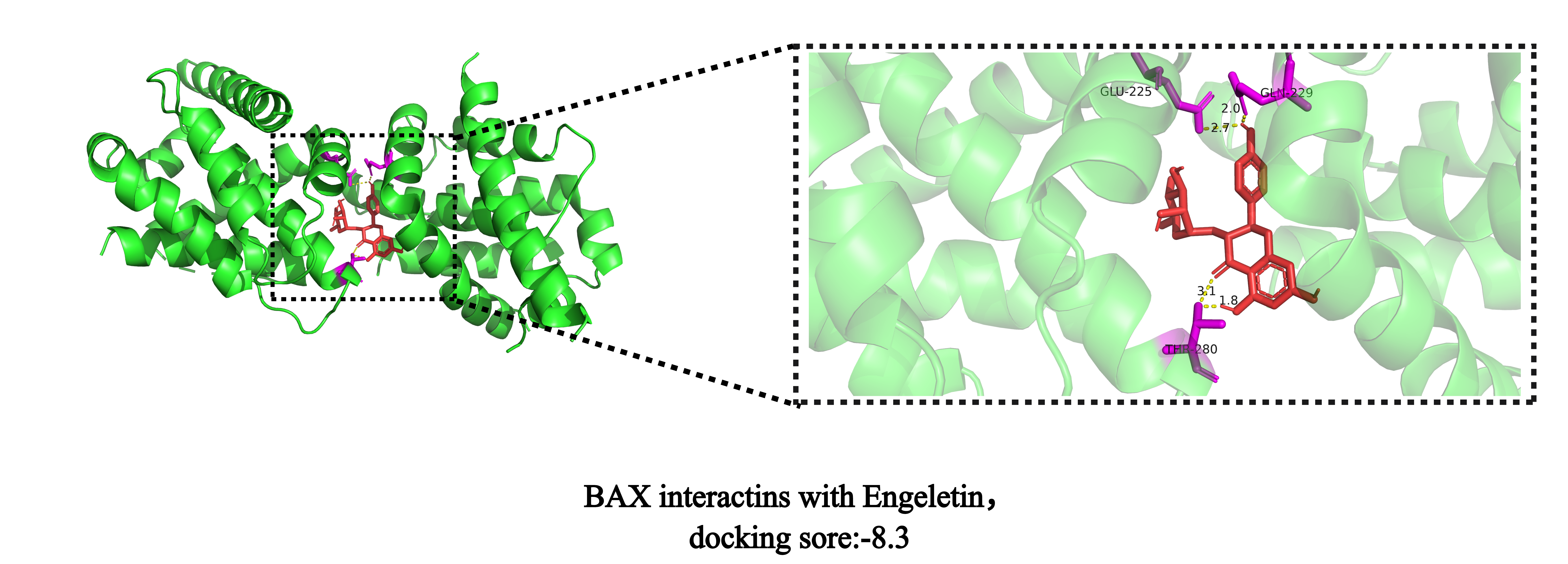

Supplement: Supplementary file 1 [file ijms-26-11446-s001.zip › BAX/combination.png]

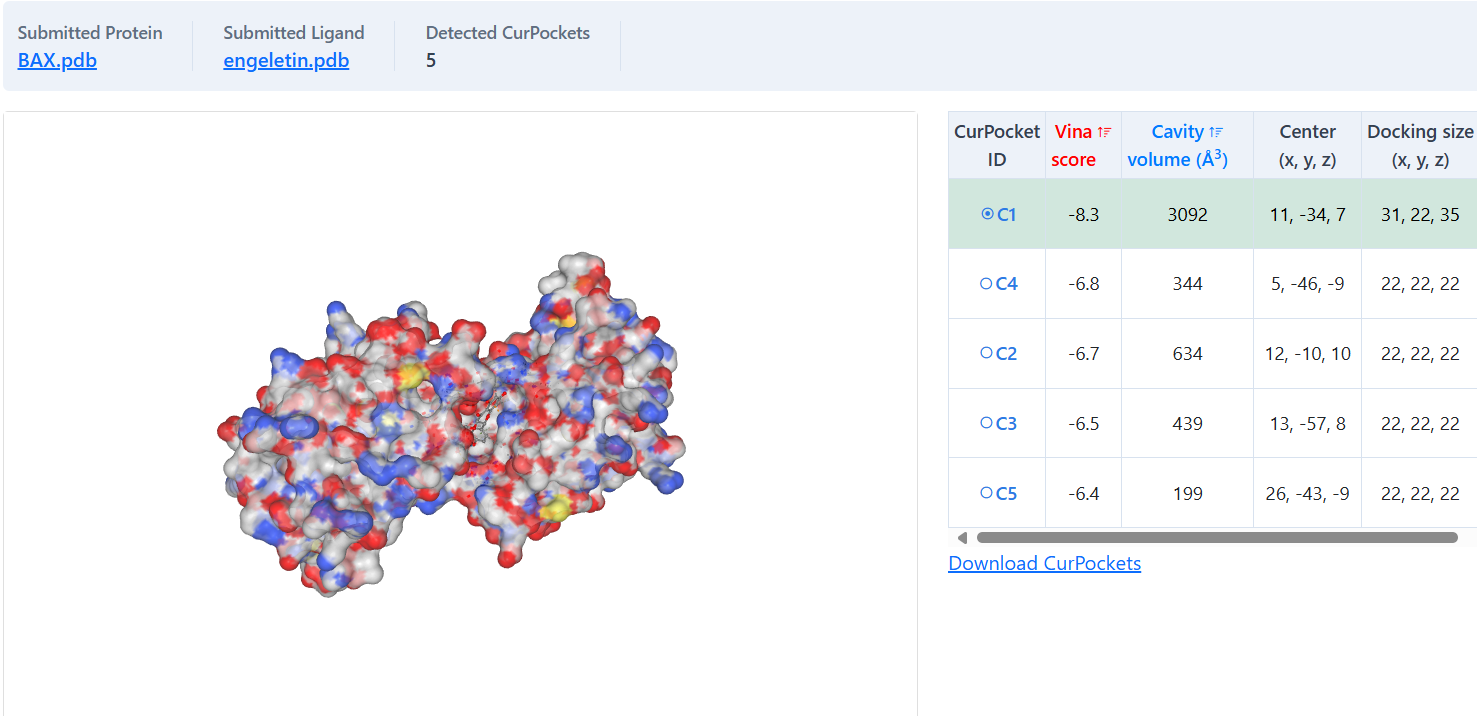

Supplement: Supplementary file 1 [file ijms-26-11446-s001.zip › BAX/free energy.png]

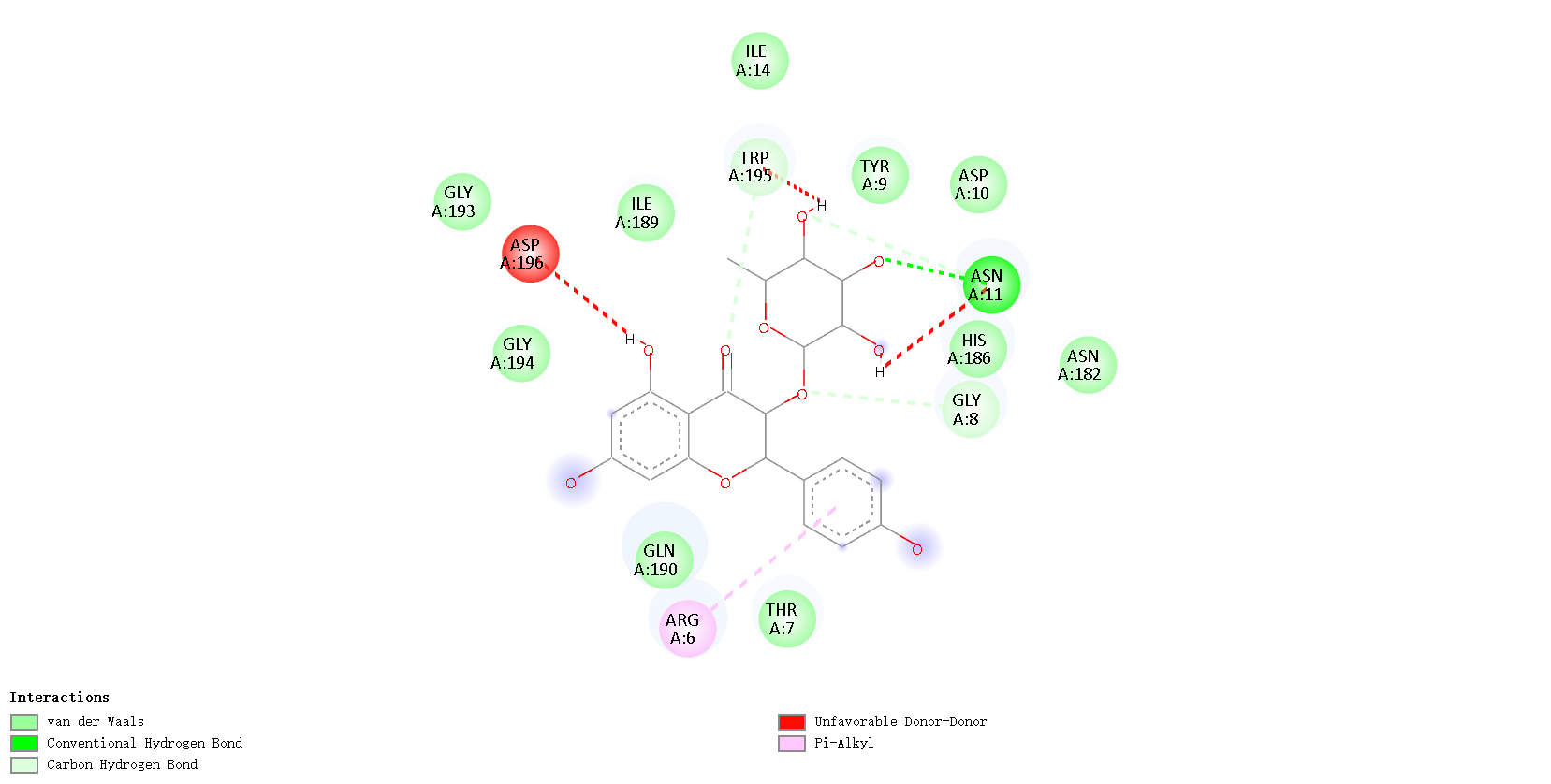

Supplement: Supplementary file 1 [file ijms-26-11446-s001.zip › Bcl-2/2D.png]

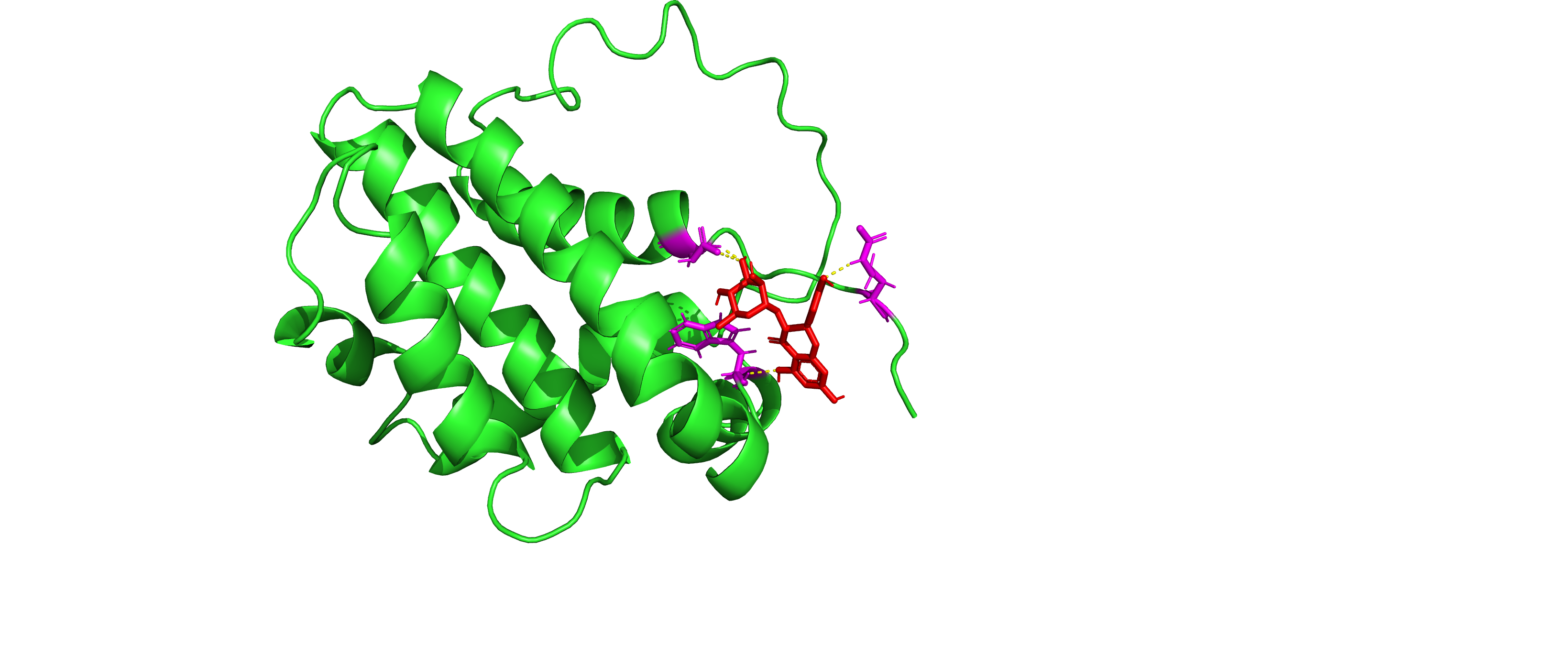

Supplement: Supplementary file 1 [file ijms-26-11446-s001.zip › Bcl-2/big.png]

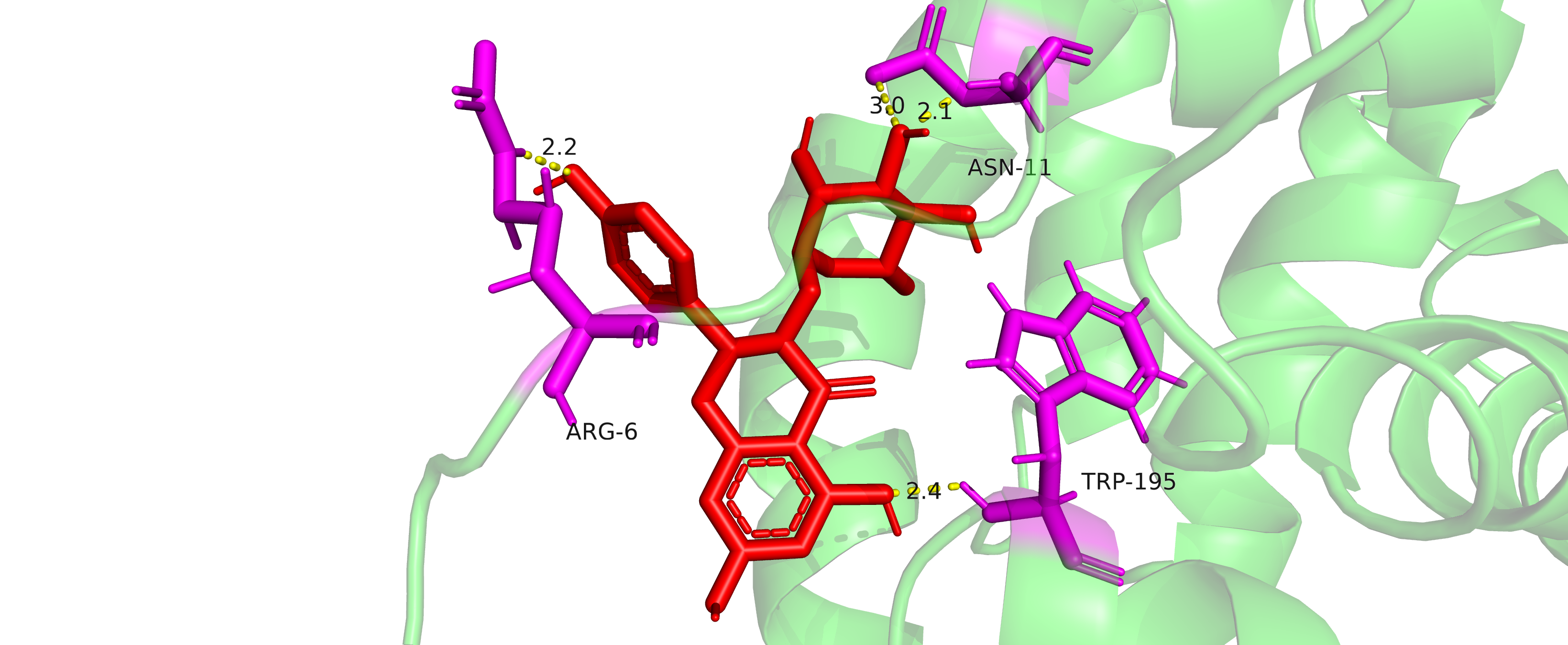

Supplement: Supplementary file 1 [file ijms-26-11446-s001.zip › Bcl-2/small.png]

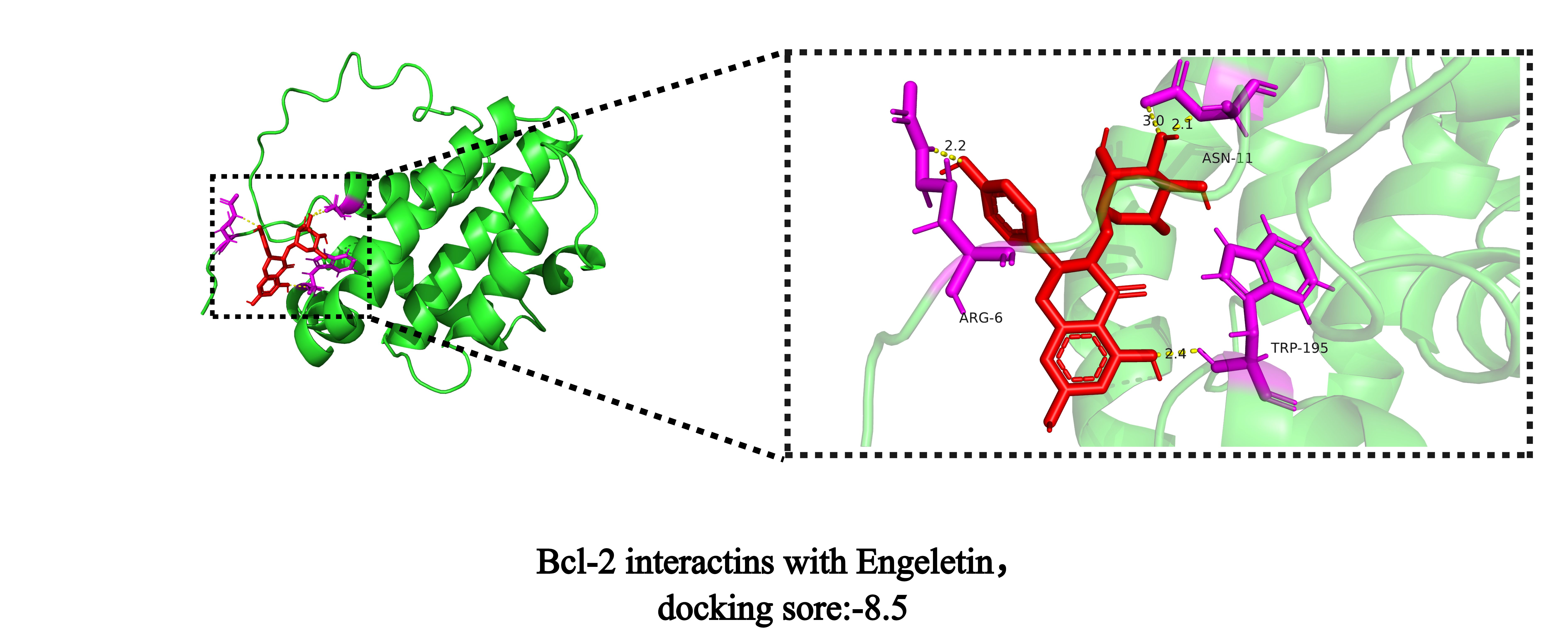

Supplement: Supplementary file 1 [file ijms-26-11446-s001.zip › Bcl-2/combination.png]

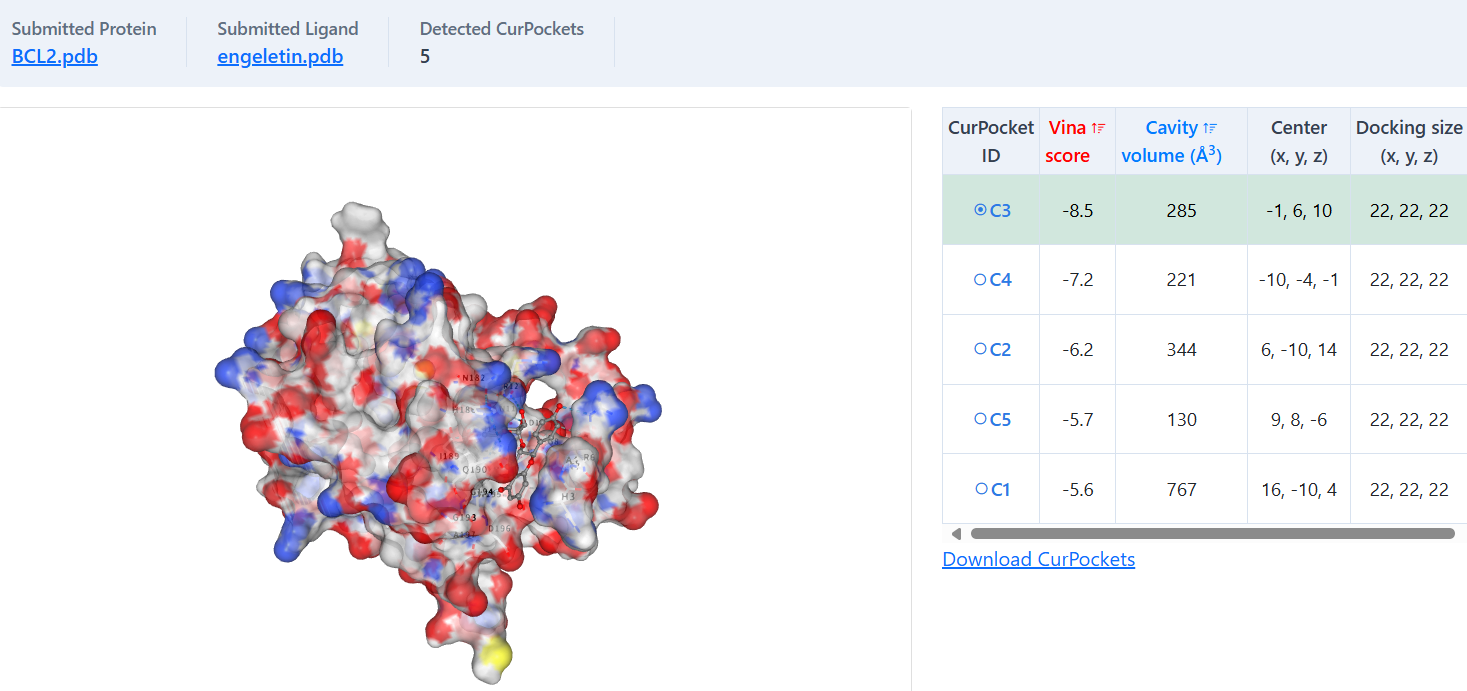

Supplement: Supplementary file 1 [file ijms-26-11446-s001.zip › Bcl-2/free energy.png]

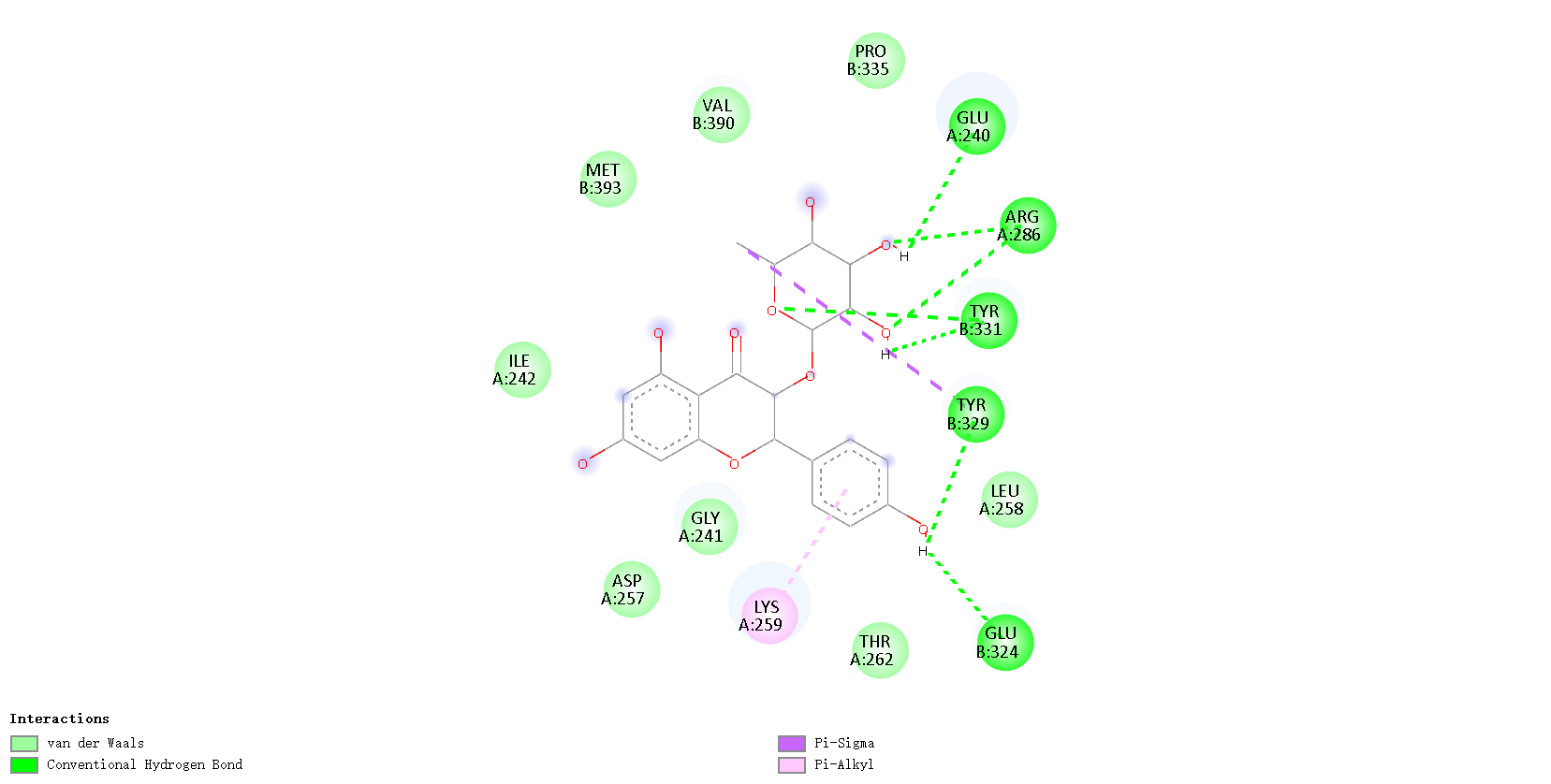

Supplement: Supplementary file 1 [file ijms-26-11446-s001.zip › Caspase-3/2D.png]

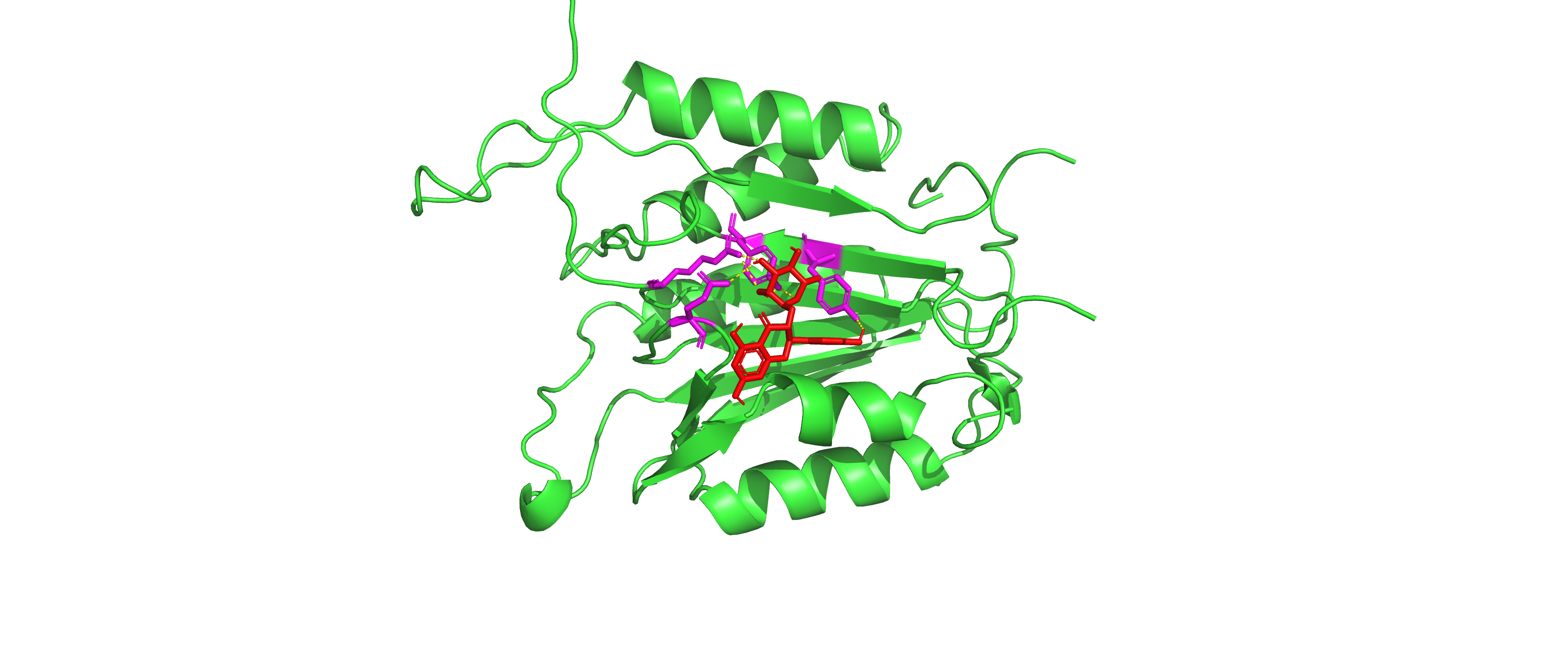

Supplement: Supplementary file 1 [file ijms-26-11446-s001.zip › Caspase-3/big.png]

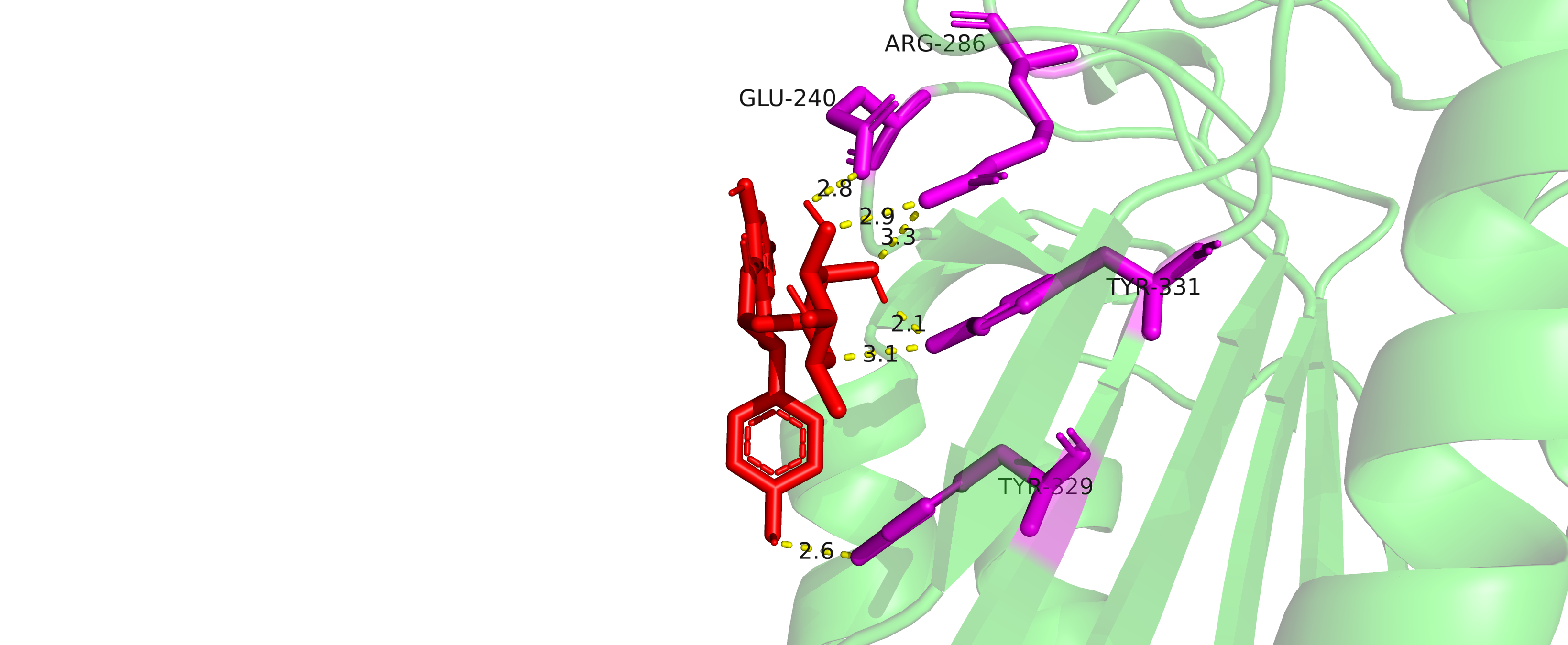

Supplement: Supplementary file 1 [file ijms-26-11446-s001.zip › Caspase-3/small.png]

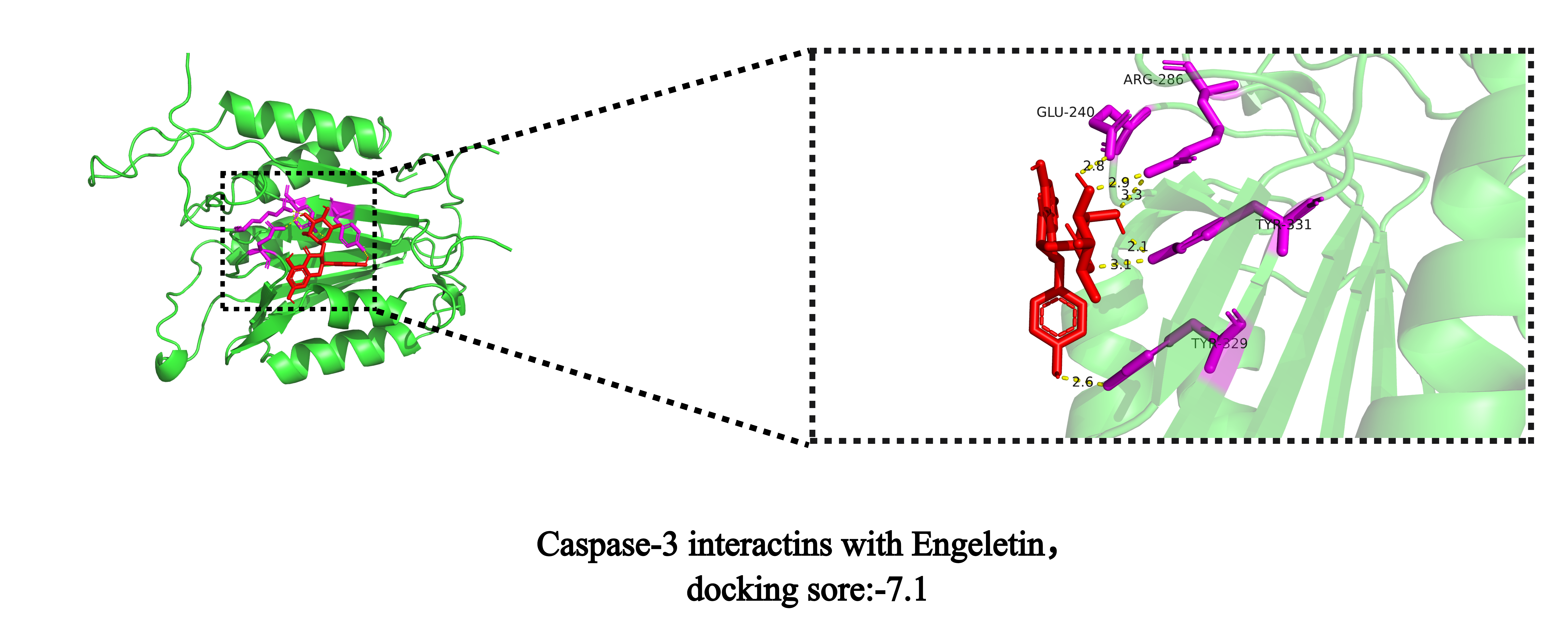

Supplement: Supplementary file 1 [file ijms-26-11446-s001.zip › Caspase-3/combination.png]

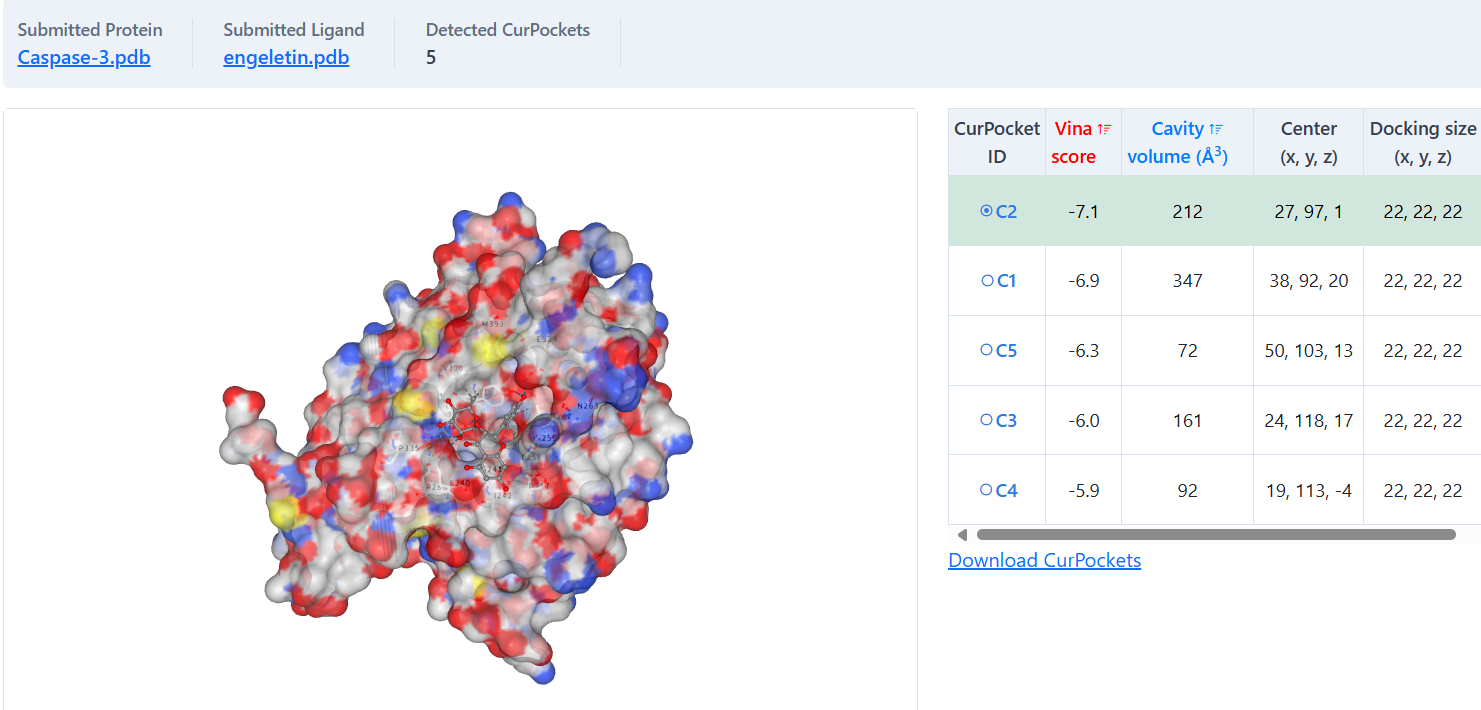

Supplement: Supplementary file 1 [file ijms-26-11446-s001.zip › Caspase-3/free energy.png]

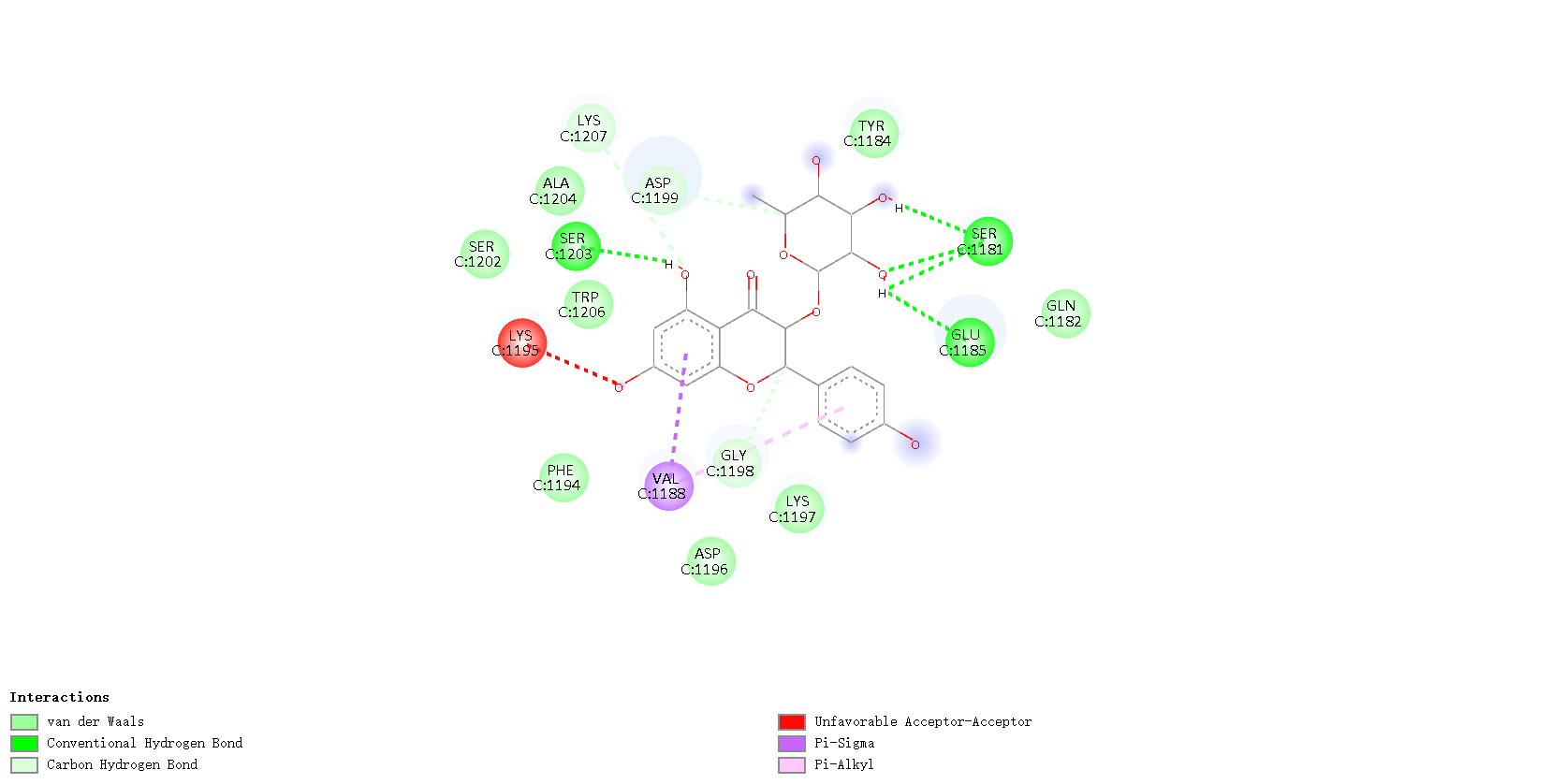

Supplement: Supplementary file 1 [file ijms-26-11446-s001.zip › foxo/2D.png]

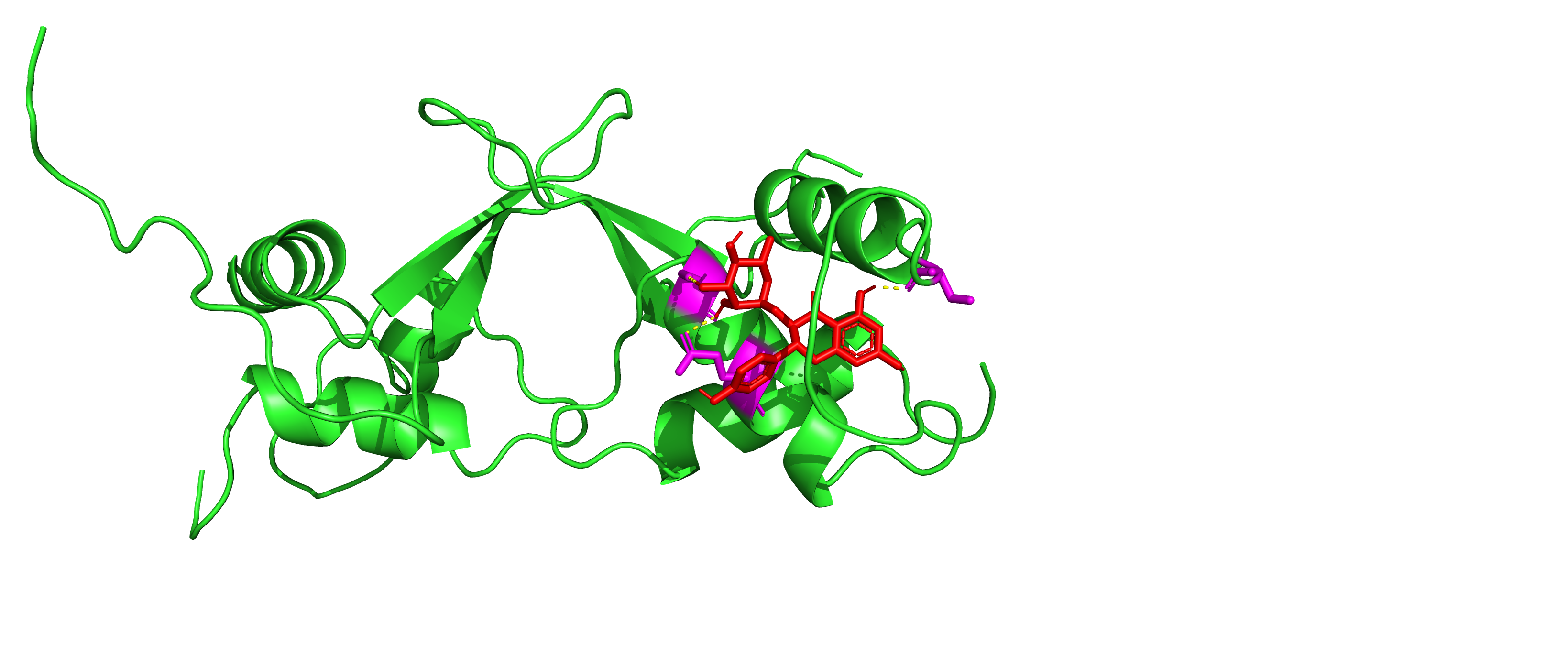

Supplement: Supplementary file 1 [file ijms-26-11446-s001.zip › foxo/big.png]

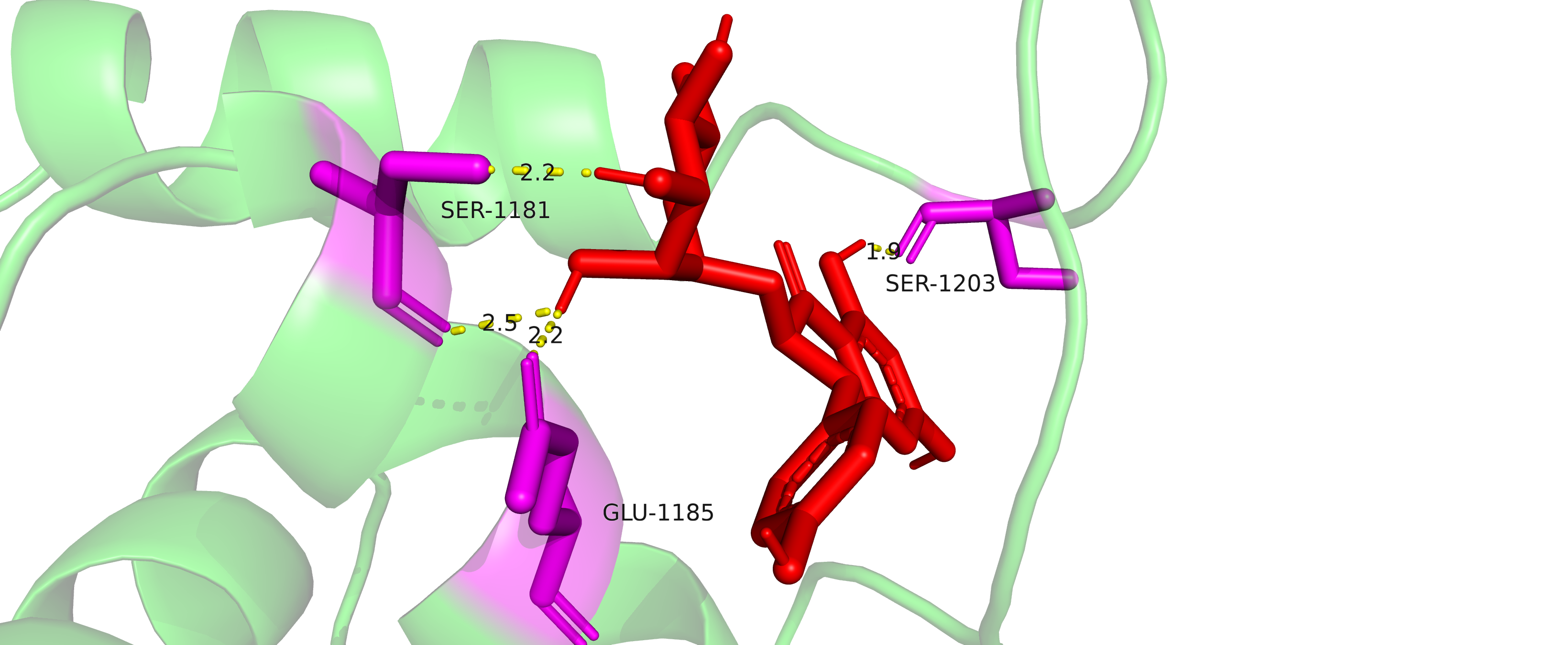

Supplement: Supplementary file 1 [file ijms-26-11446-s001.zip › foxo/small.png]

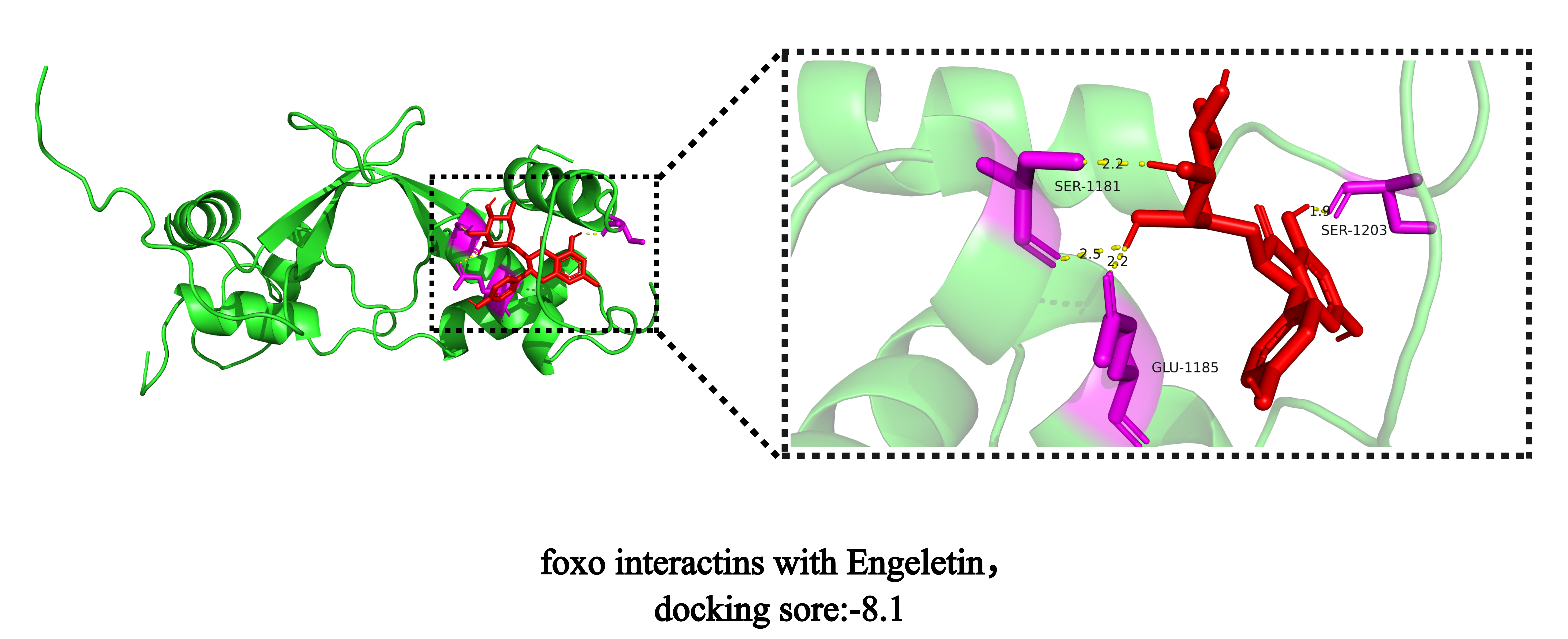

Supplement: Supplementary file 1 [file ijms-26-11446-s001.zip › foxo/combination.png]

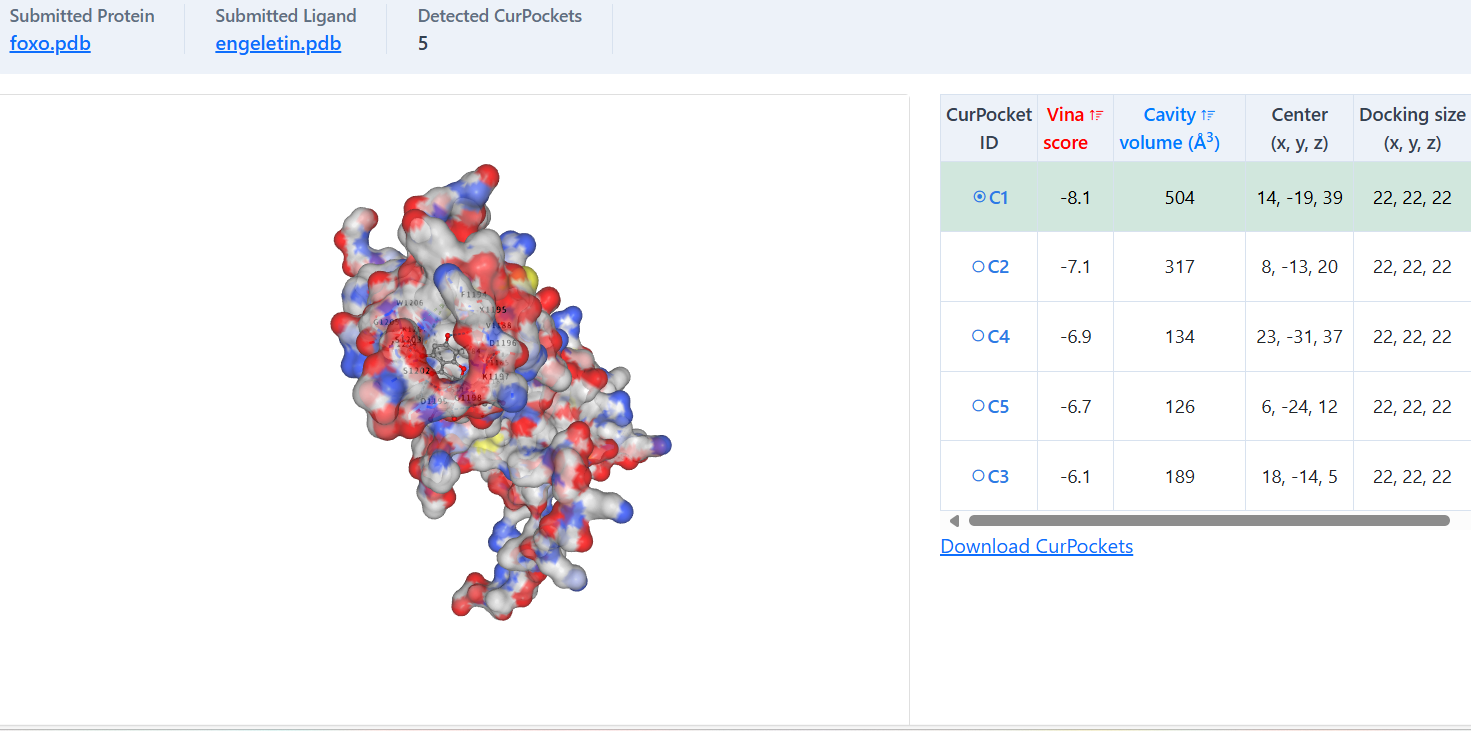

Supplement: Supplementary file 1 [file ijms-26-11446-s001.zip › foxo/free energy.png]

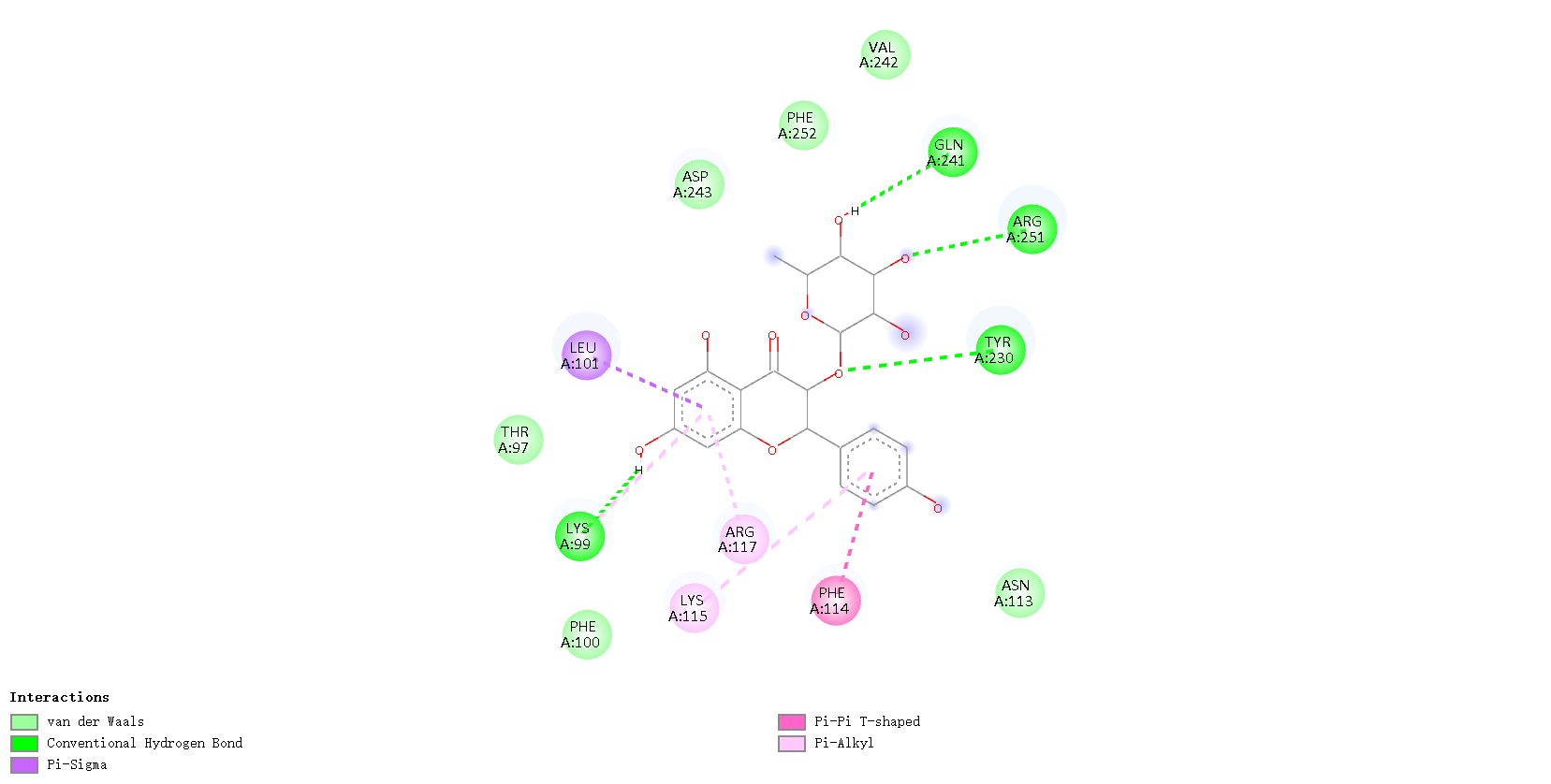

Supplement: Supplementary file 1 [file ijms-26-11446-s001.zip › Hif1/2D.png]

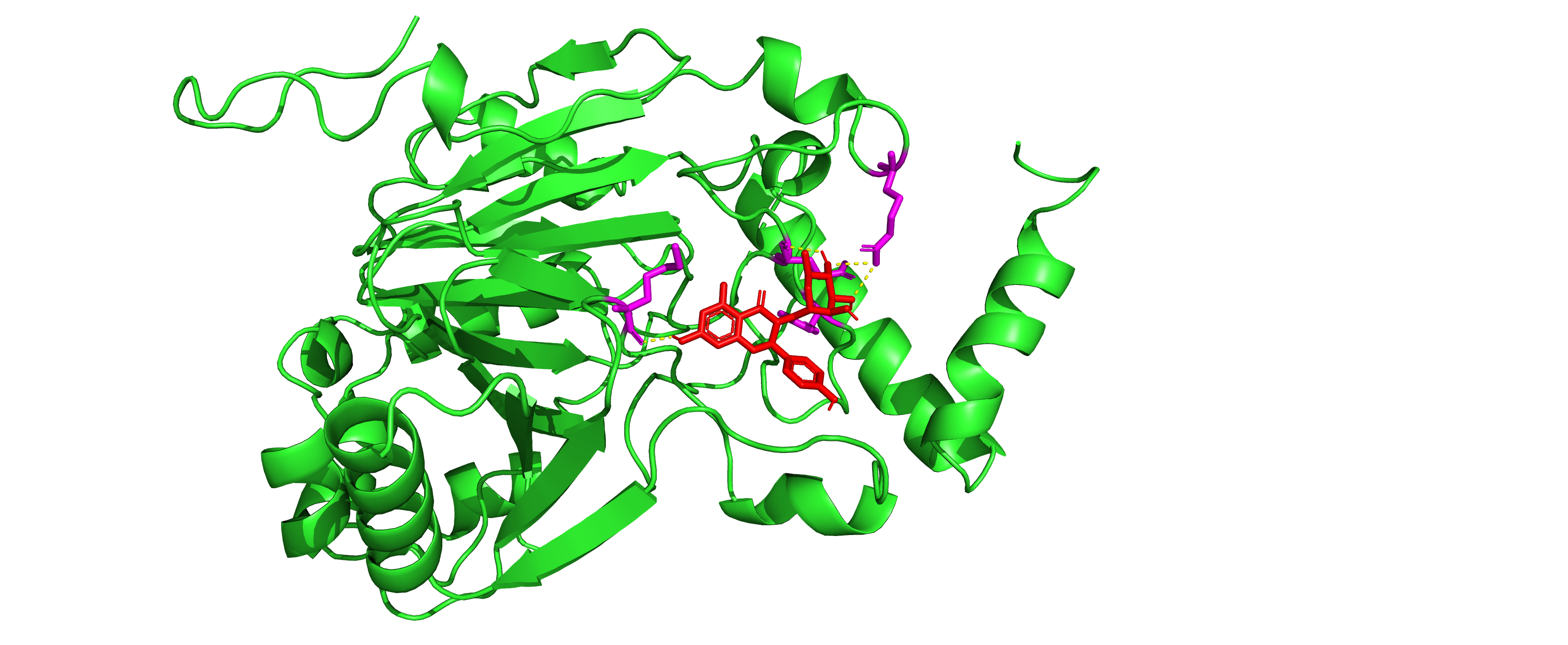

Supplement: Supplementary file 1 [file ijms-26-11446-s001.zip › Hif1/big.png]

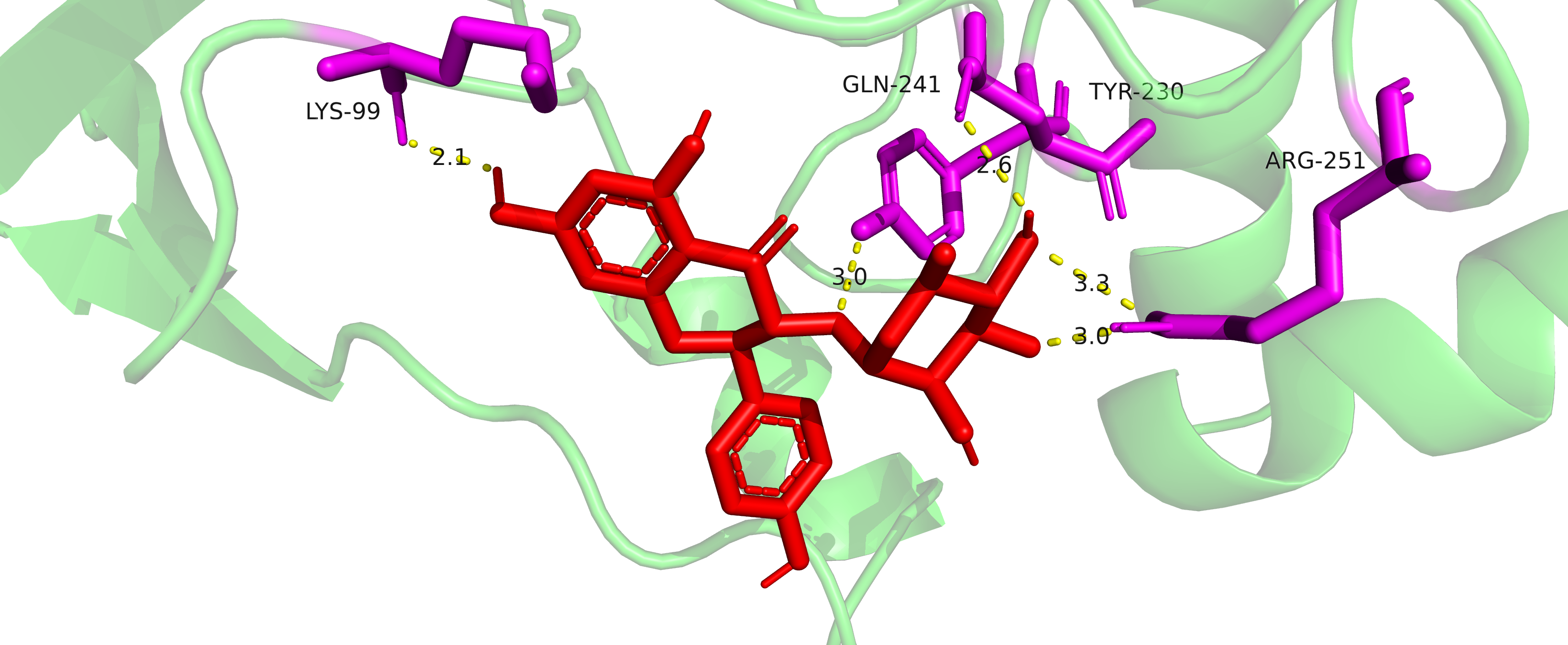

Supplement: Supplementary file 1 [file ijms-26-11446-s001.zip › Hif1/small.png]

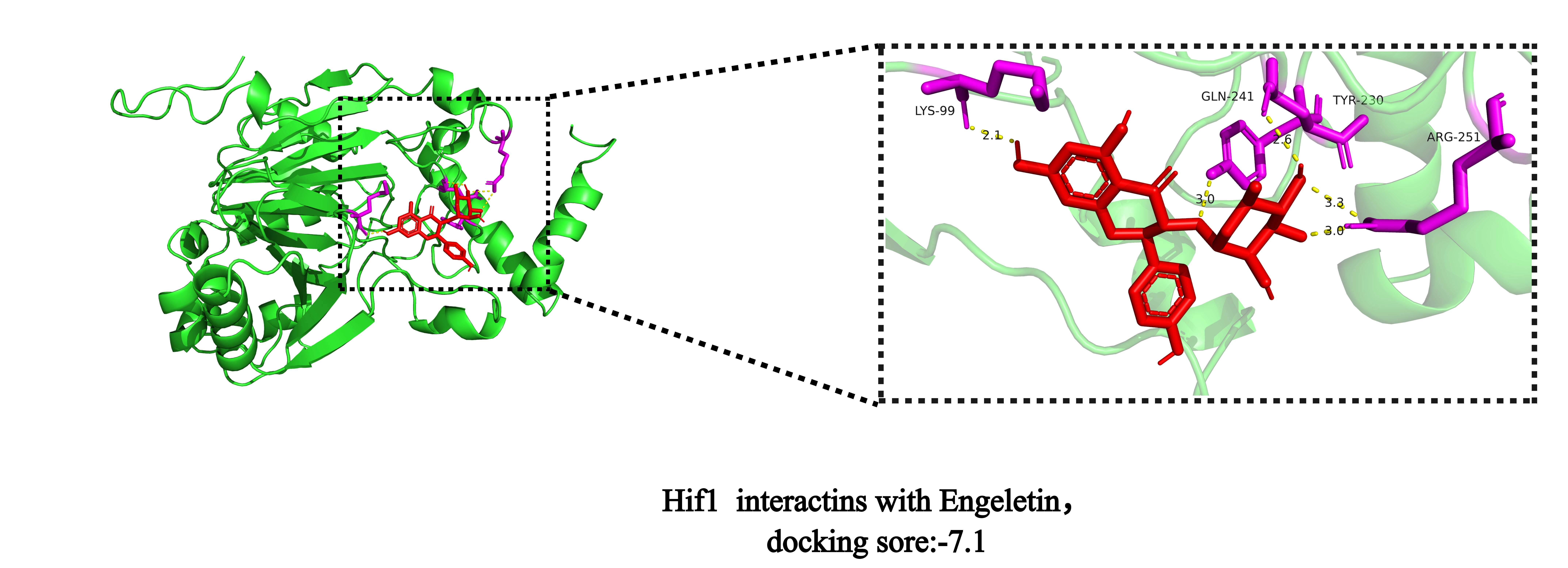

Supplement: Supplementary file 1 [file ijms-26-11446-s001.zip › Hif1/combination.png]

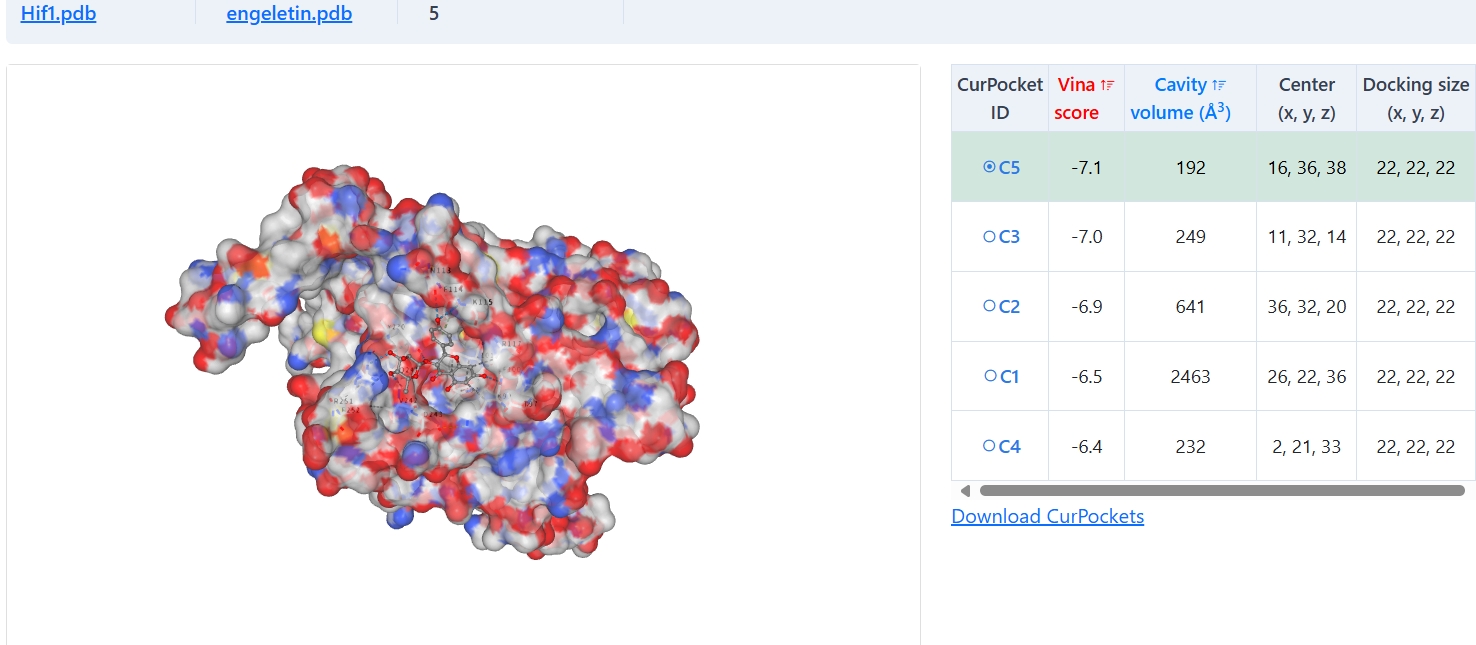

Supplement: Supplementary file 1 [file ijms-26-11446-s001.zip › Hif1/free energy.png]

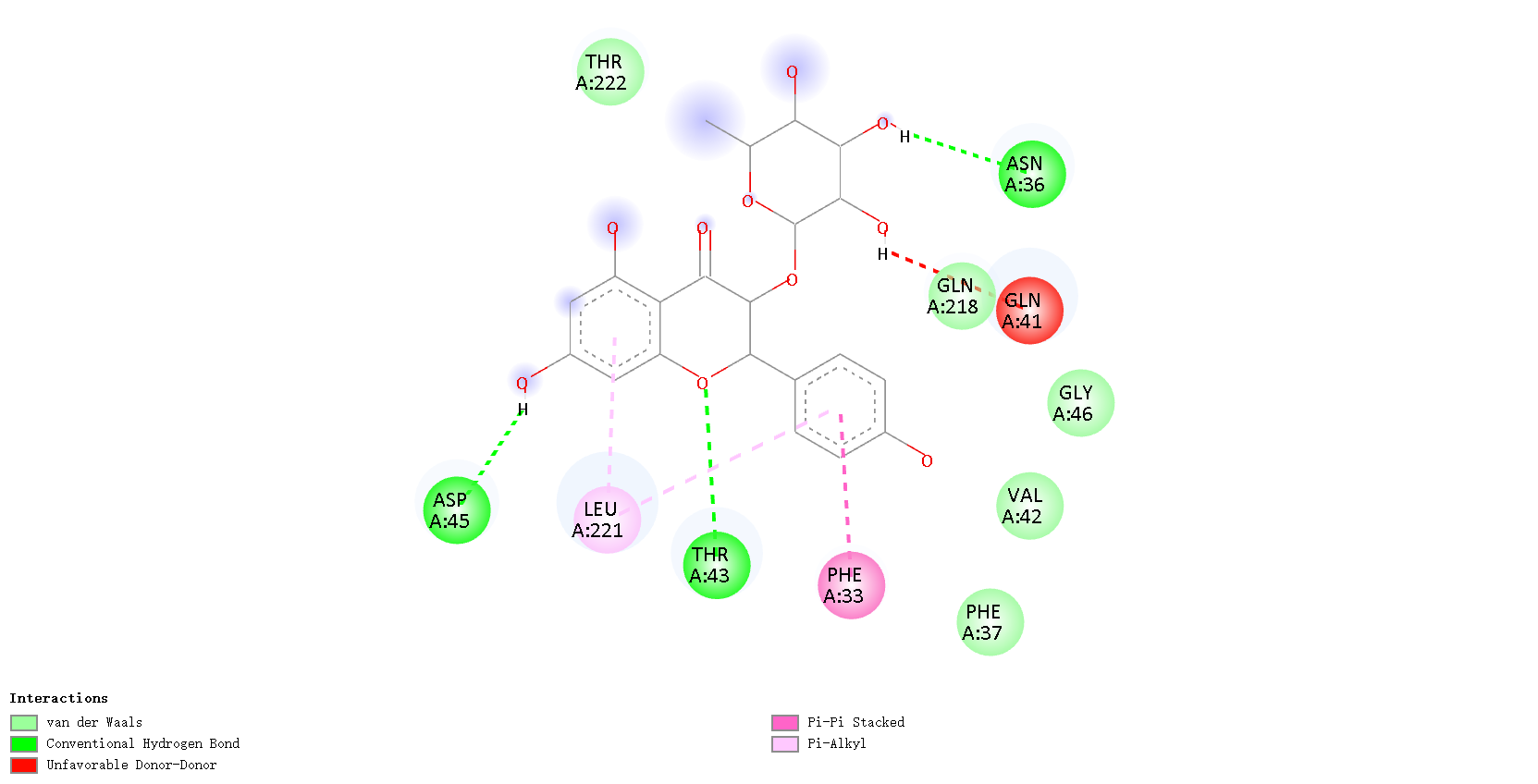

Supplement: Supplementary file 1 [file ijms-26-11446-s001.zip › HO-1/2D.png]

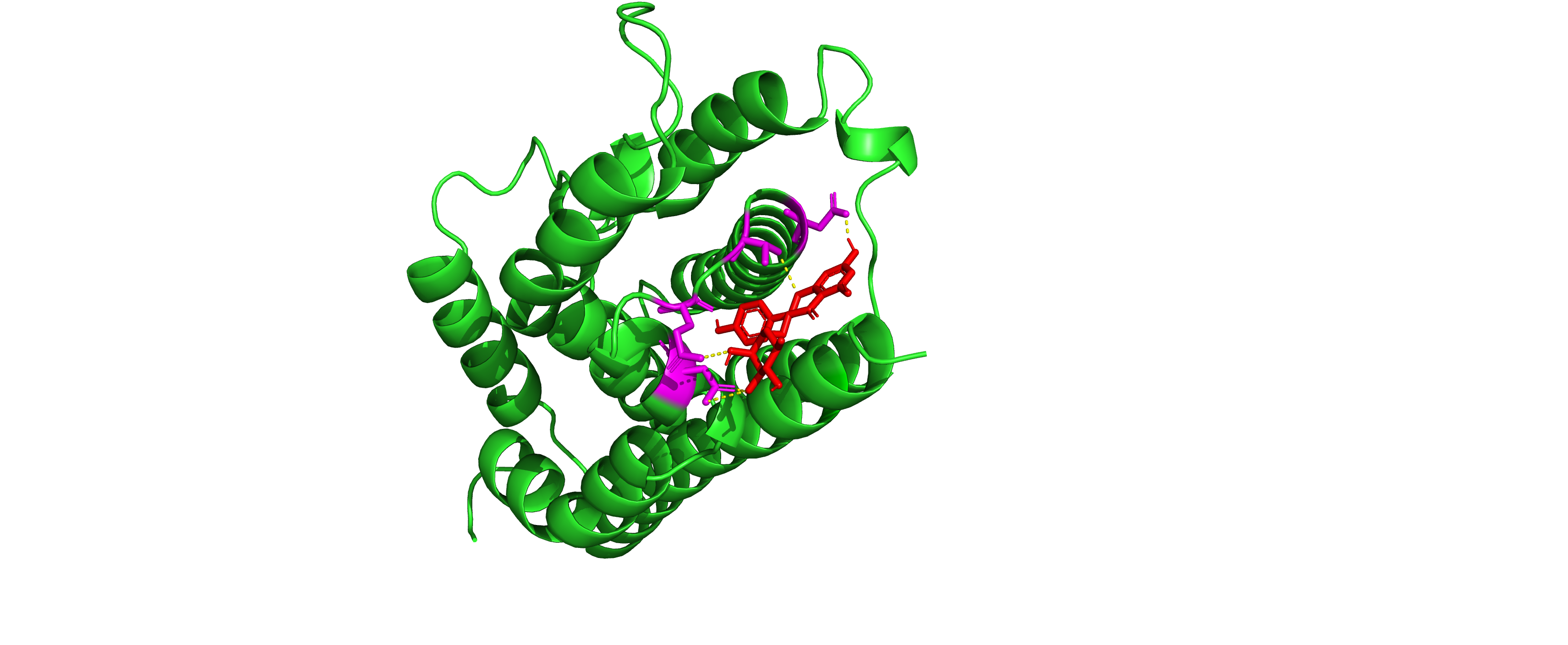

Supplement: Supplementary file 1 [file ijms-26-11446-s001.zip › HO-1/big.png]

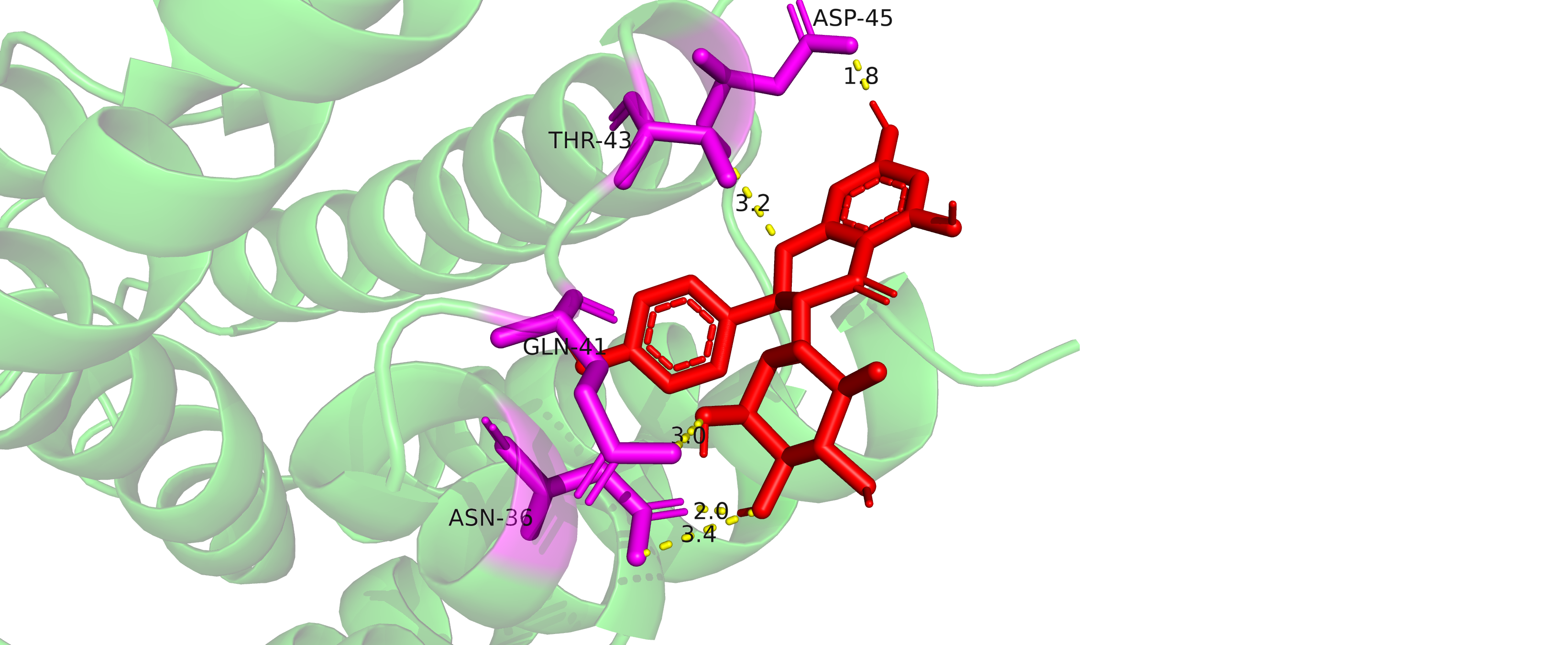

Supplement: Supplementary file 1 [file ijms-26-11446-s001.zip › HO-1/small.png]

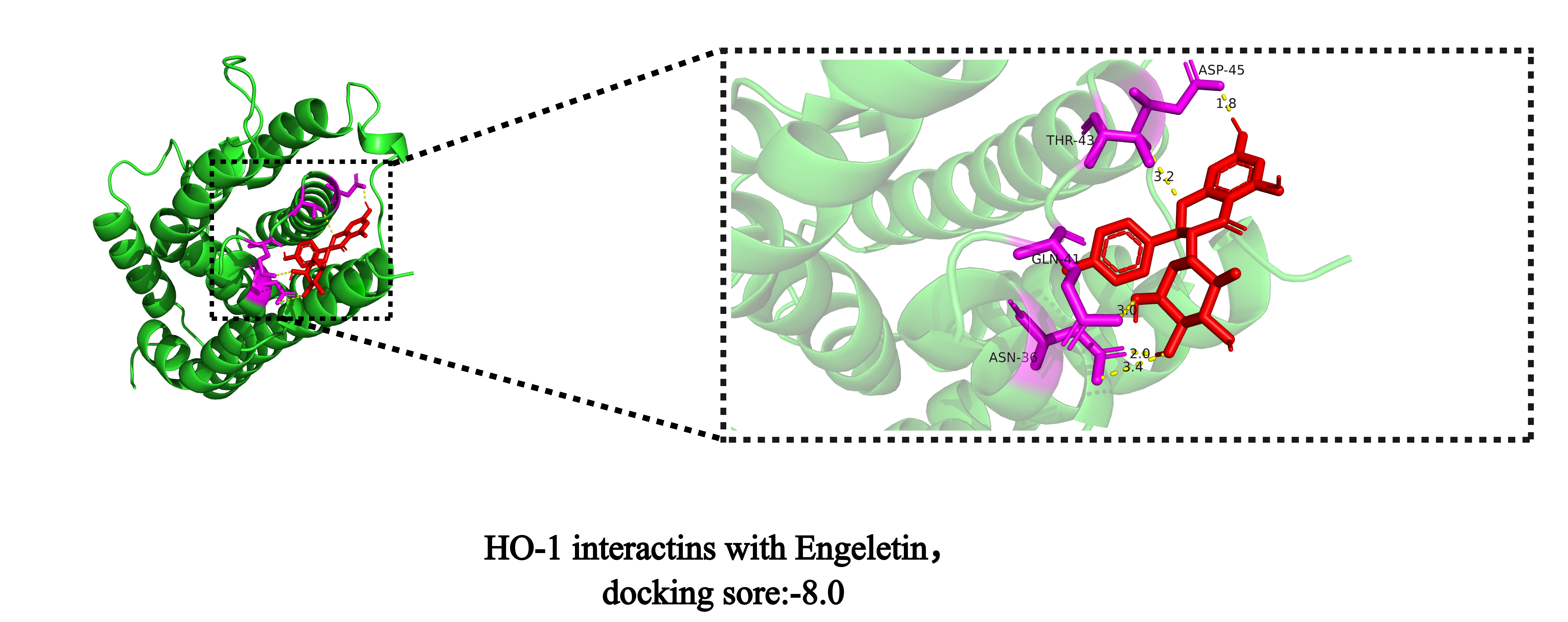

Supplement: Supplementary file 1 [file ijms-26-11446-s001.zip › HO-1/combination.png]

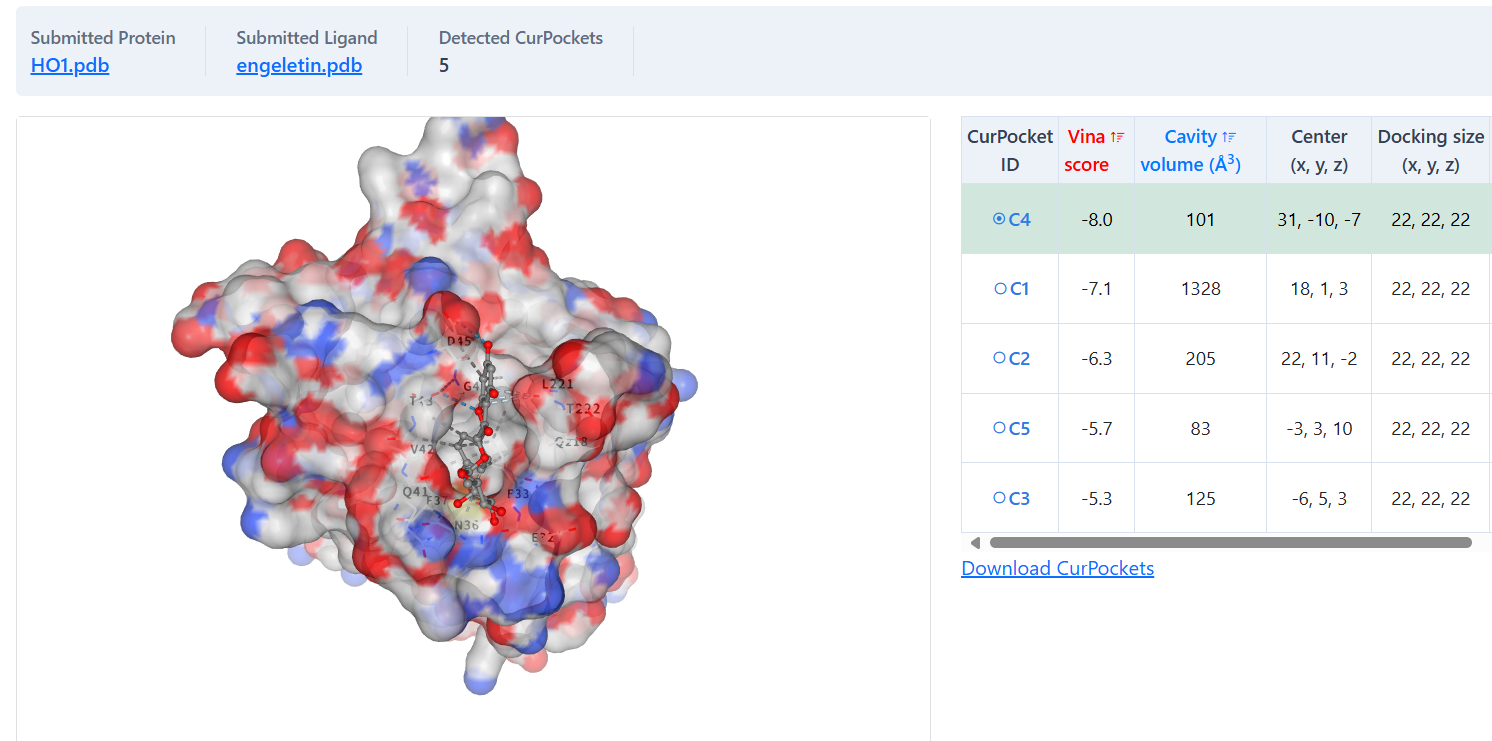

Supplement: Supplementary file 1 [file ijms-26-11446-s001.zip › HO-1/energy.png]

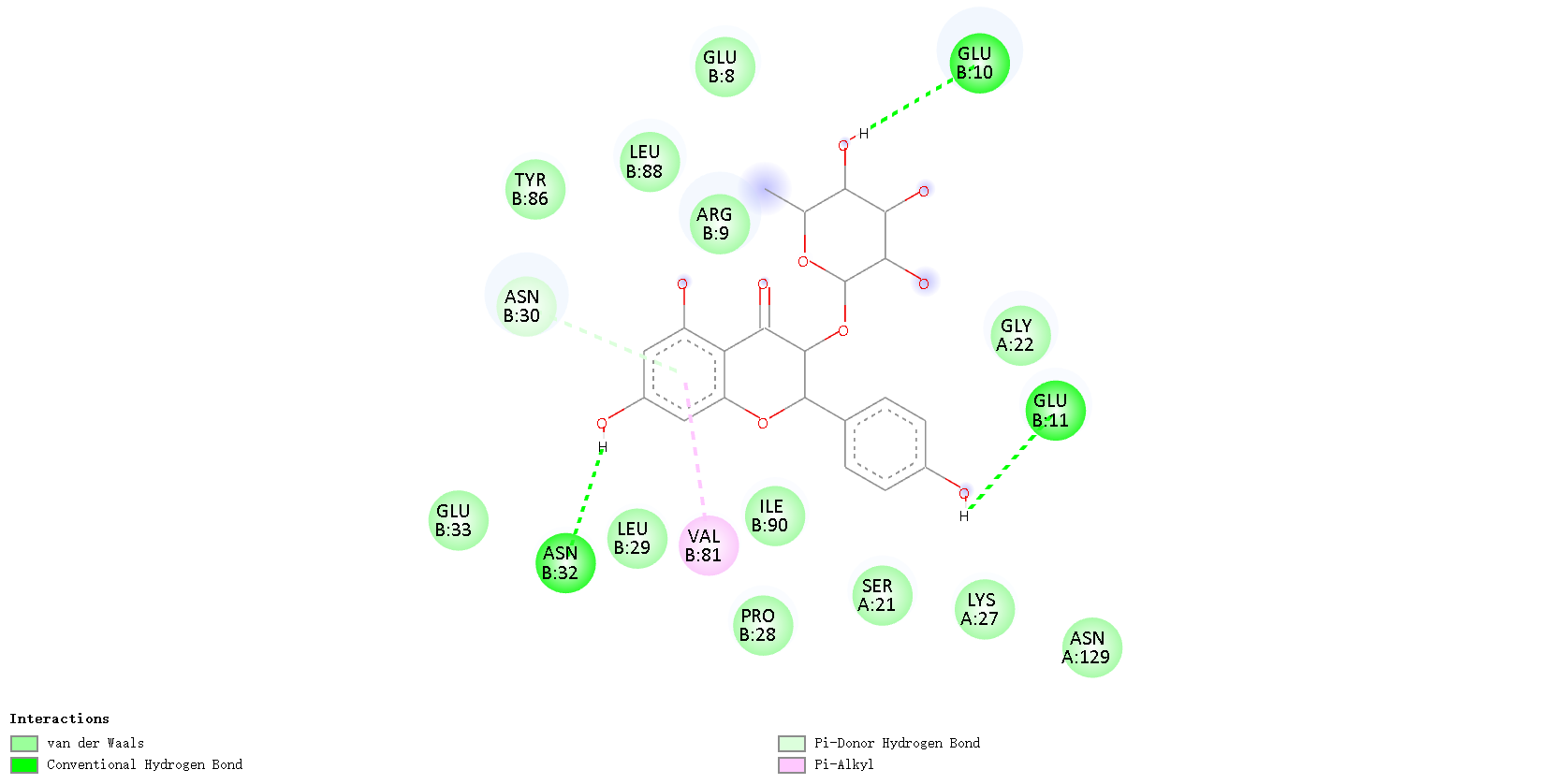

Supplement: Supplementary file 1 [file ijms-26-11446-s001.zip › IL-1/2D.png]

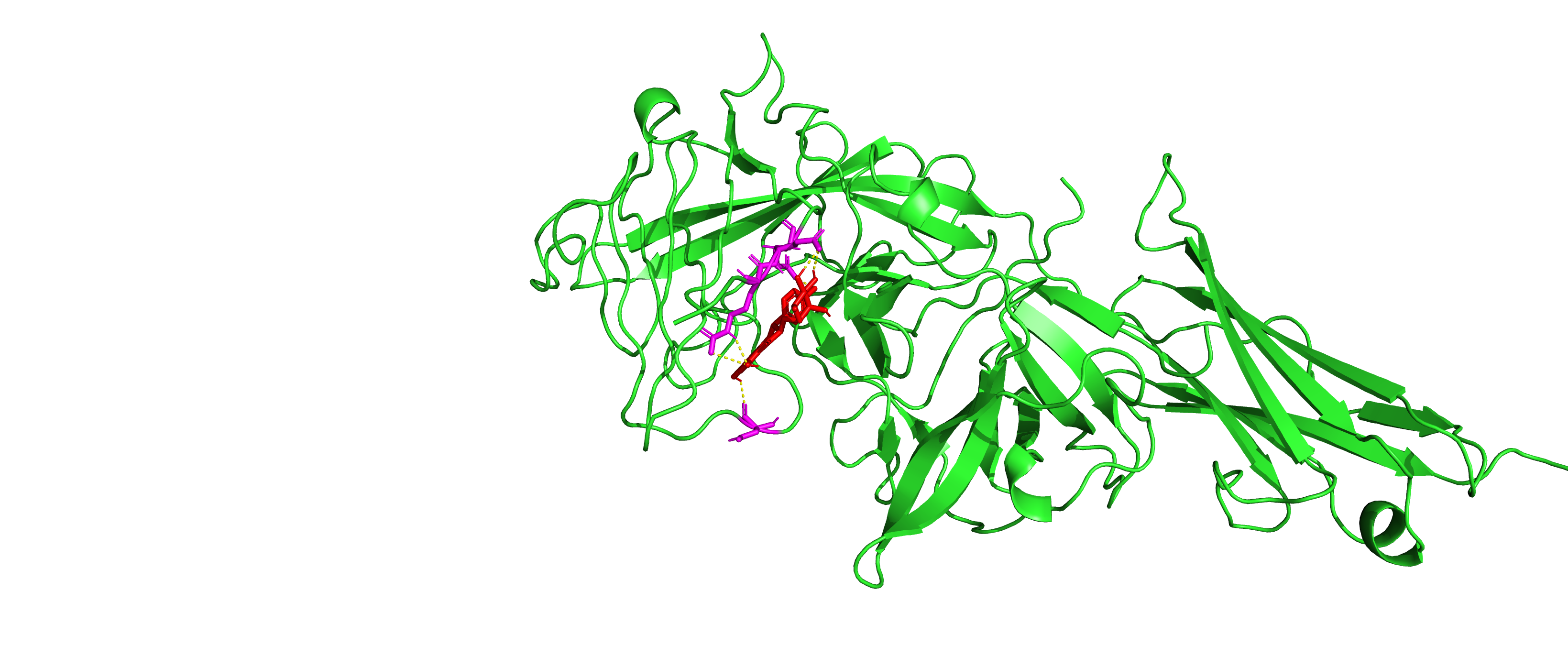

Supplement: Supplementary file 1 [file ijms-26-11446-s001.zip › IL-1/big.png]

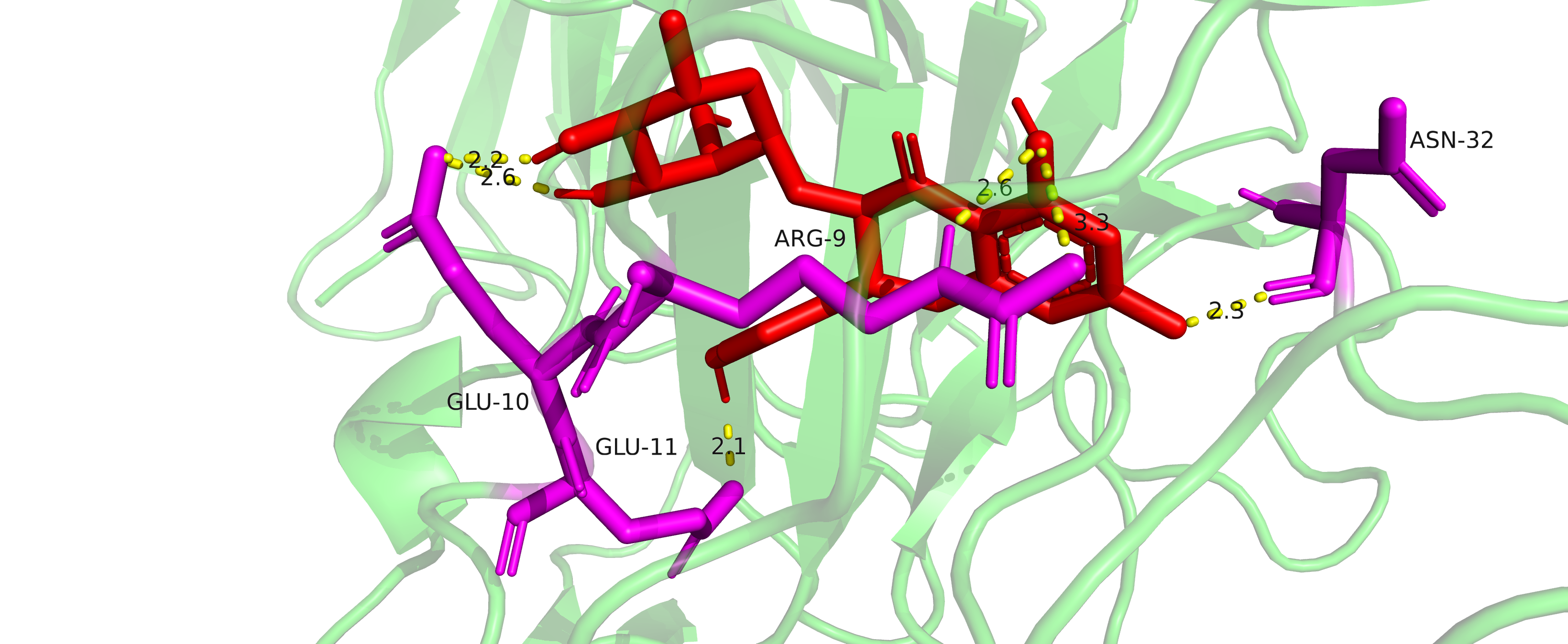

Supplement: Supplementary file 1 [file ijms-26-11446-s001.zip › IL-1/small.png]

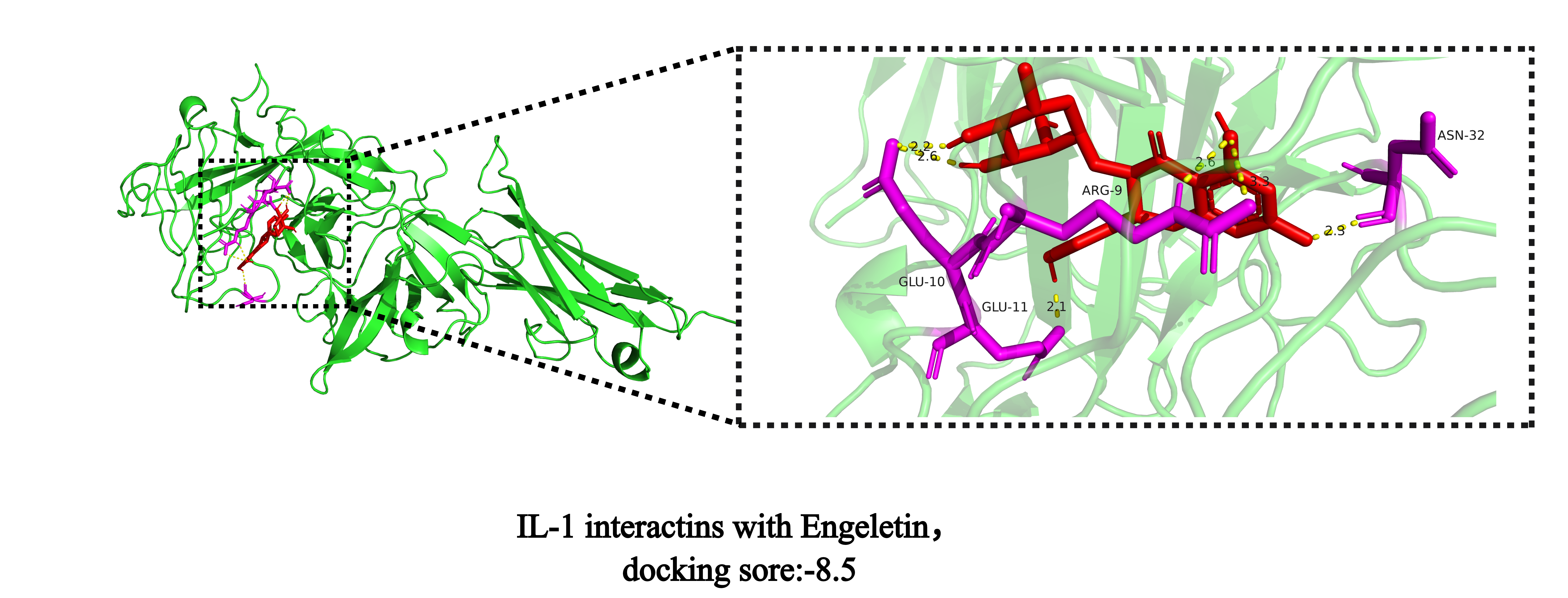

Supplement: Supplementary file 1 [file ijms-26-11446-s001.zip › IL-1/combination.png]

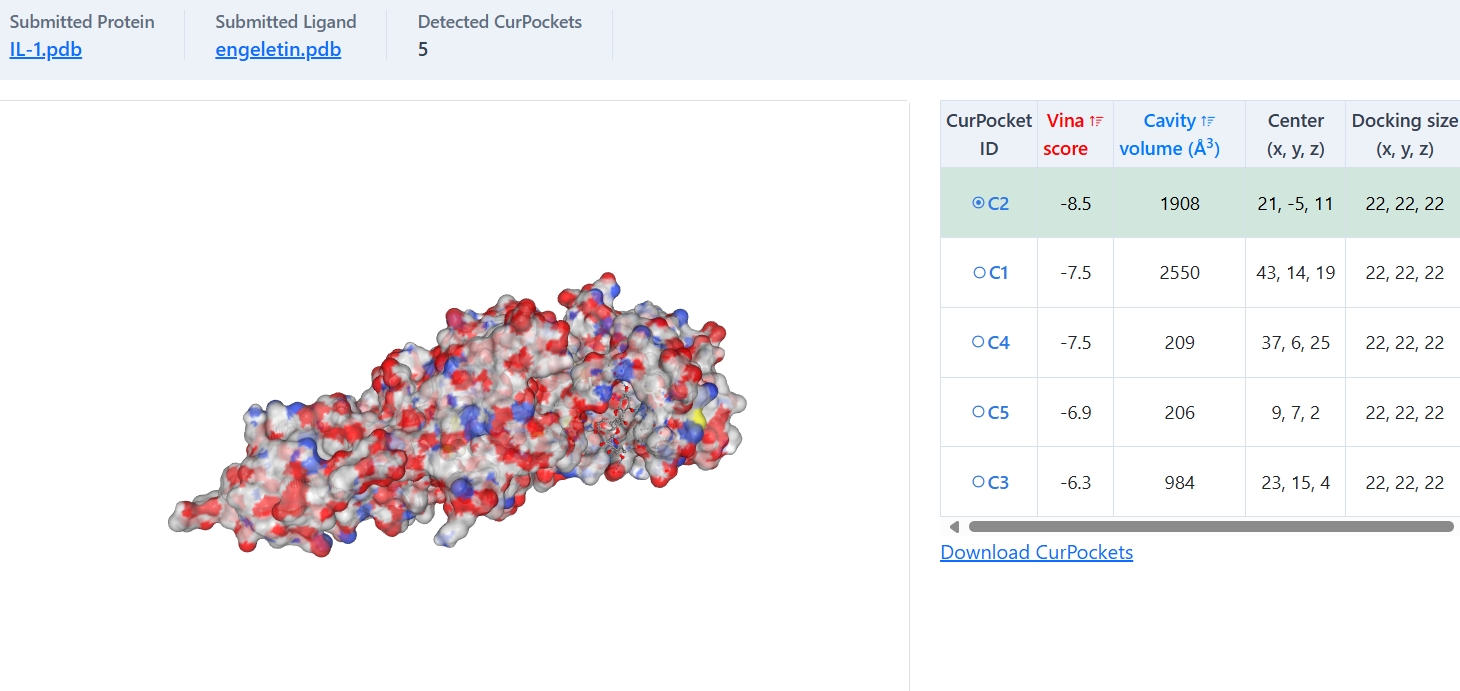

Supplement: Supplementary file 1 [file ijms-26-11446-s001.zip › IL-1/energy.png]

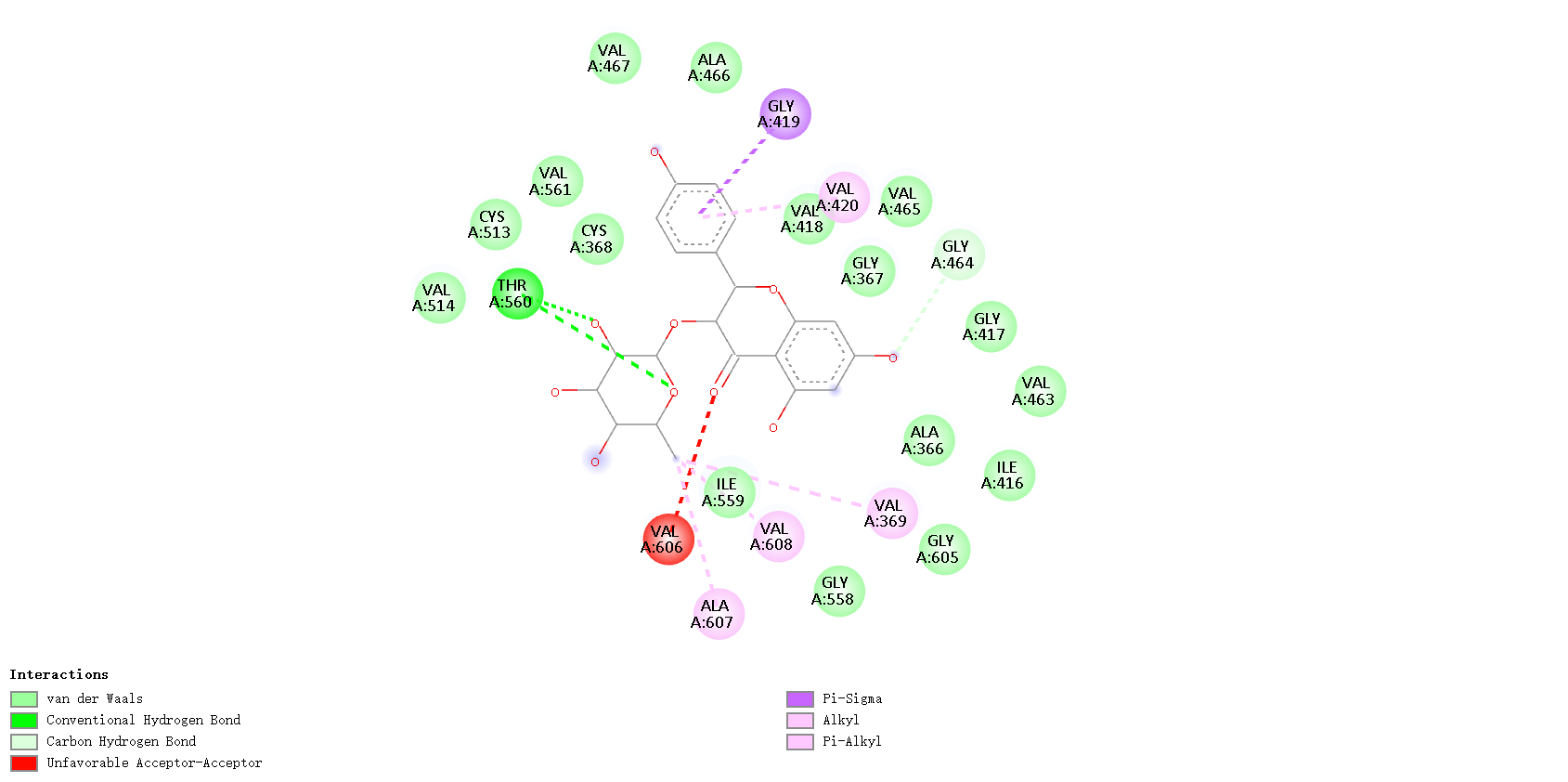

Supplement: Supplementary file 1 [file ijms-26-11446-s001.zip › keap1/2d图.png]

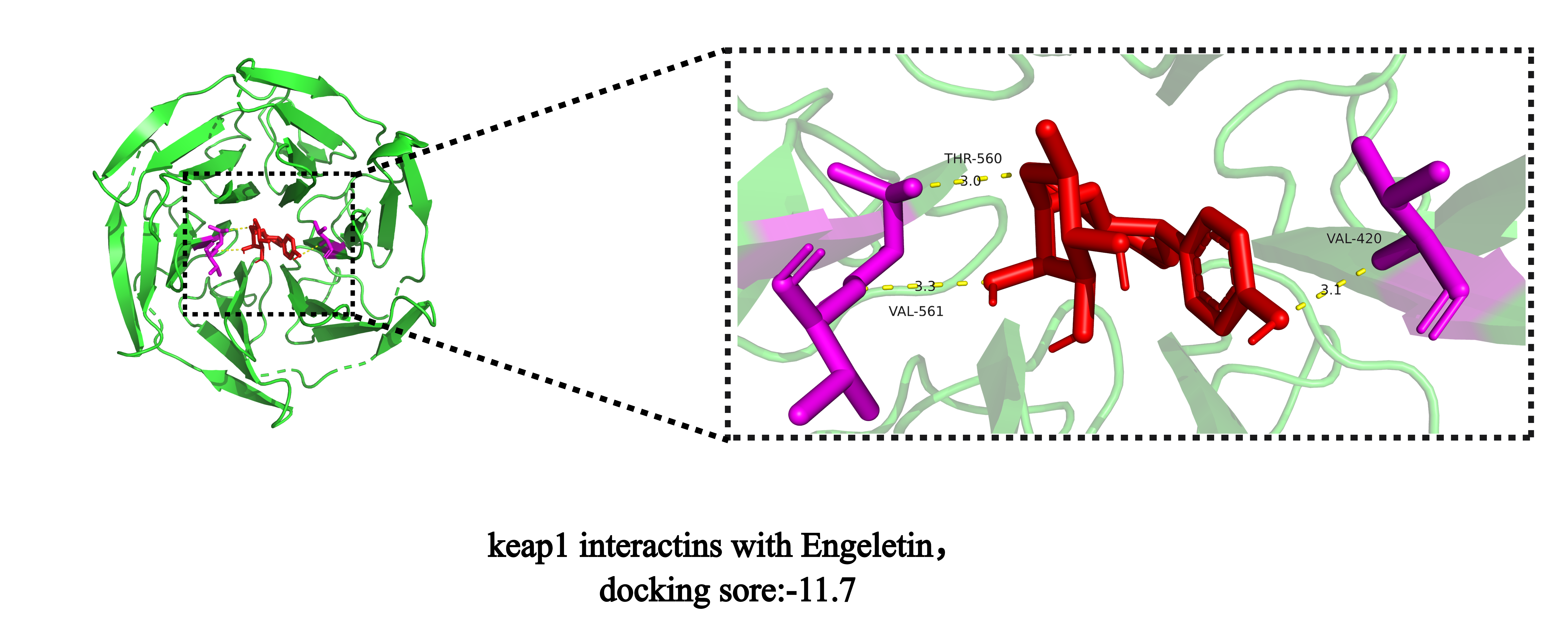

Supplement: Supplementary file 1 [file ijms-26-11446-s001.zip › keap1/keap combination.png]

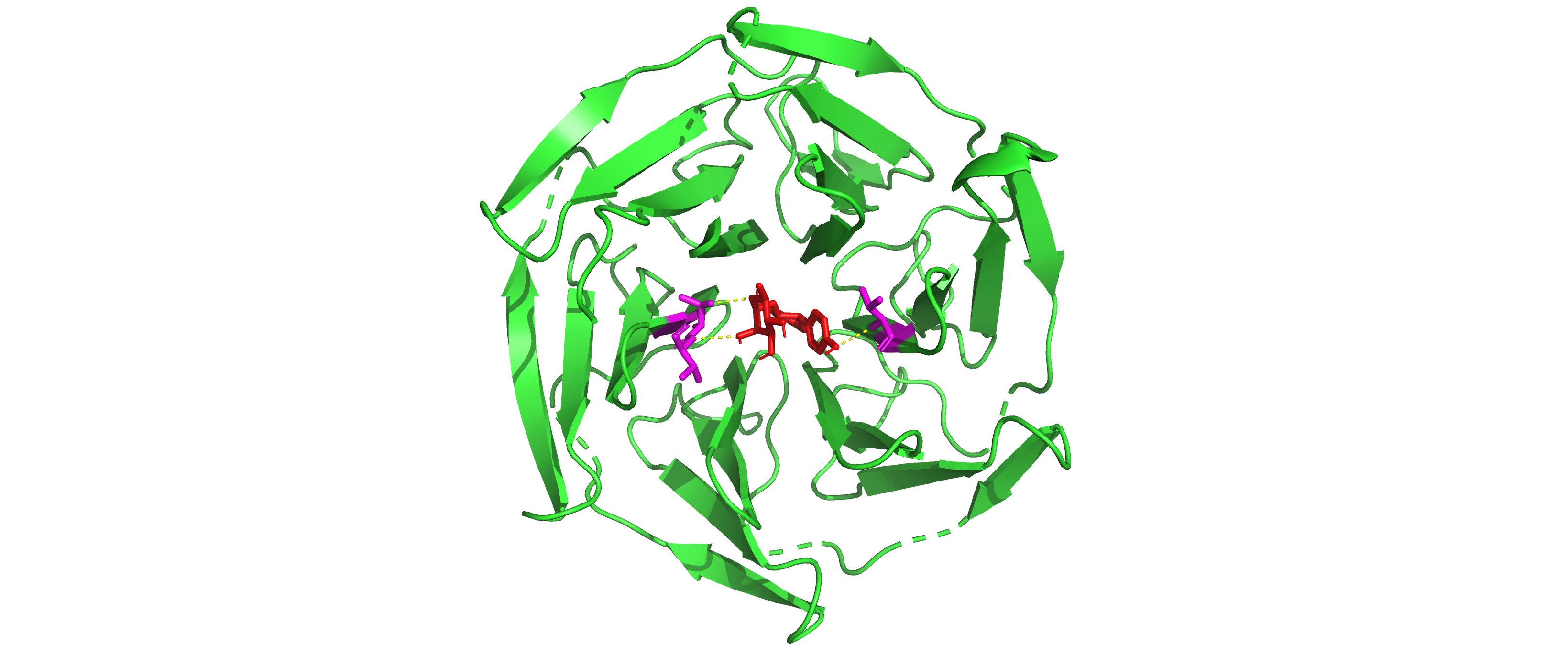

Supplement: Supplementary file 1 [file ijms-26-11446-s001.zip › keap1/big.png]

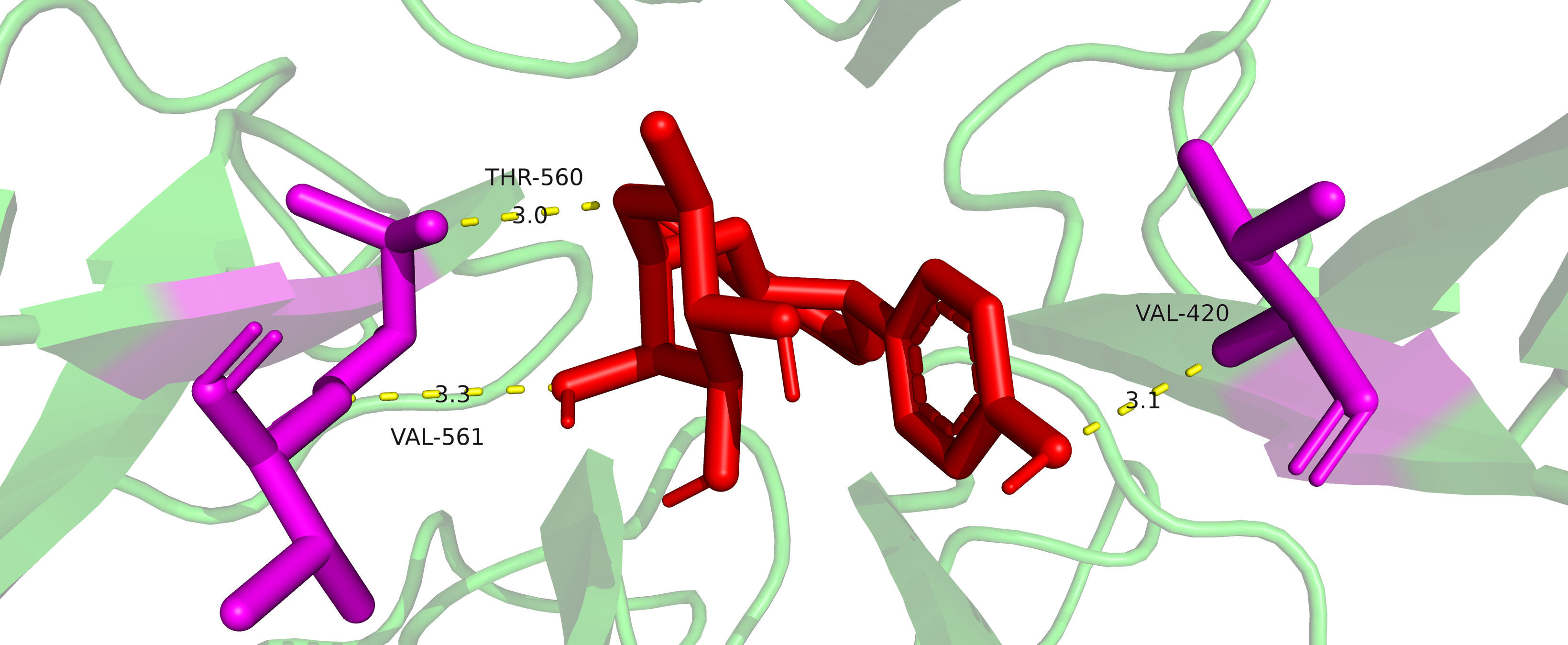

Supplement: Supplementary file 1 [file ijms-26-11446-s001.zip › keap1/small.png]

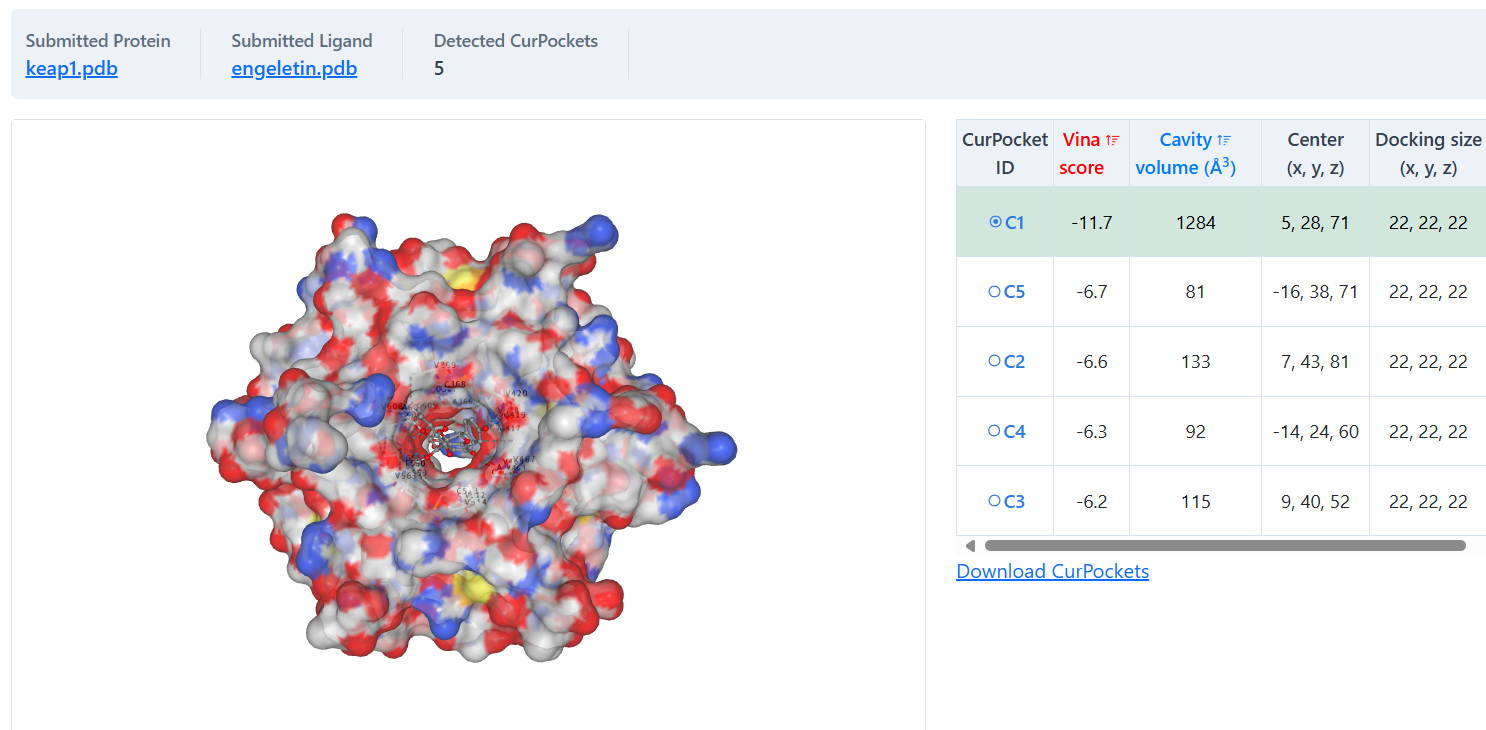

Supplement: Supplementary file 1 [file ijms-26-11446-s001.zip › keap1/free energy.png]

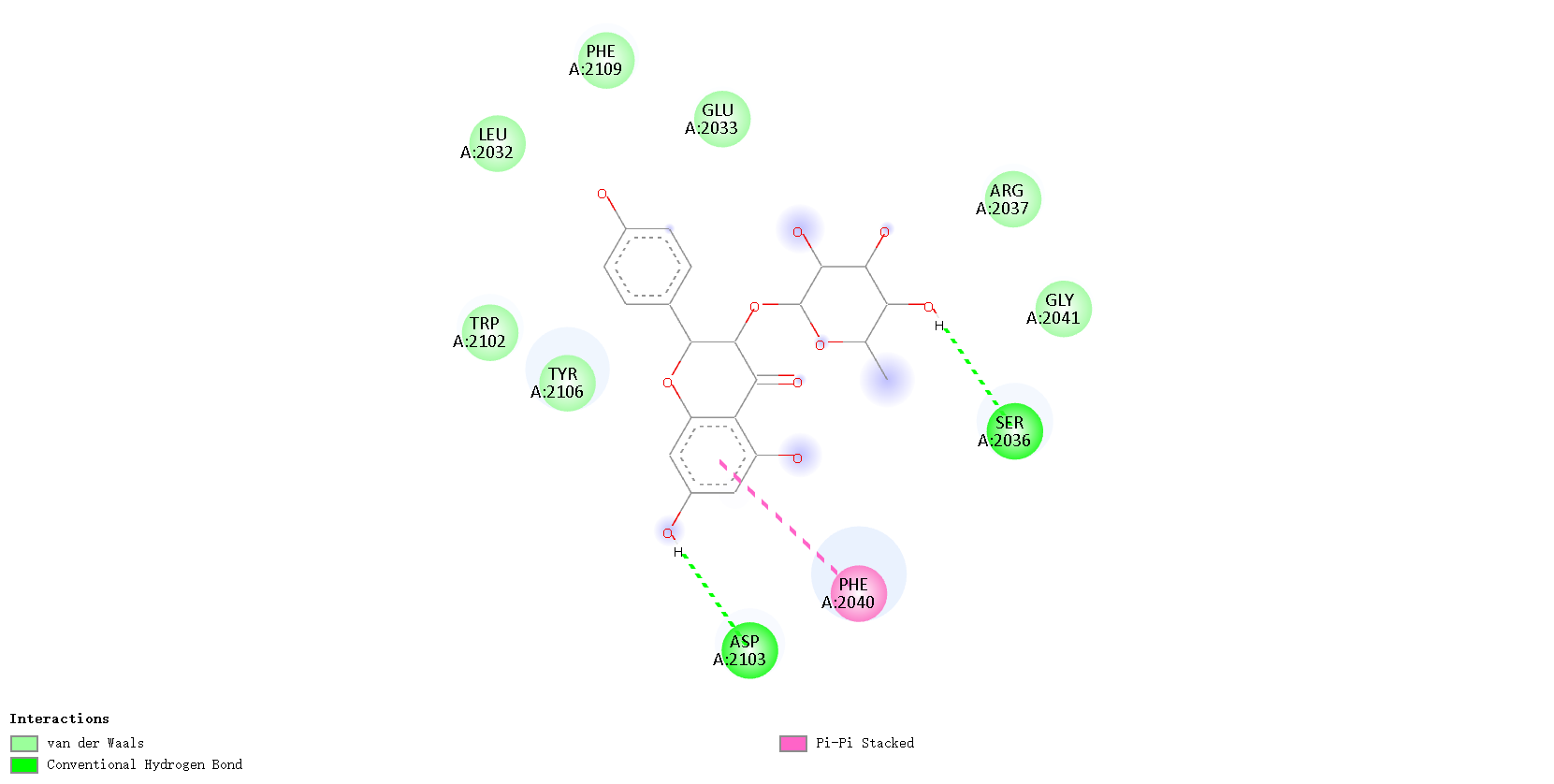

Supplement: Supplementary file 1 [file ijms-26-11446-s001.zip › MTOR/2D.png]

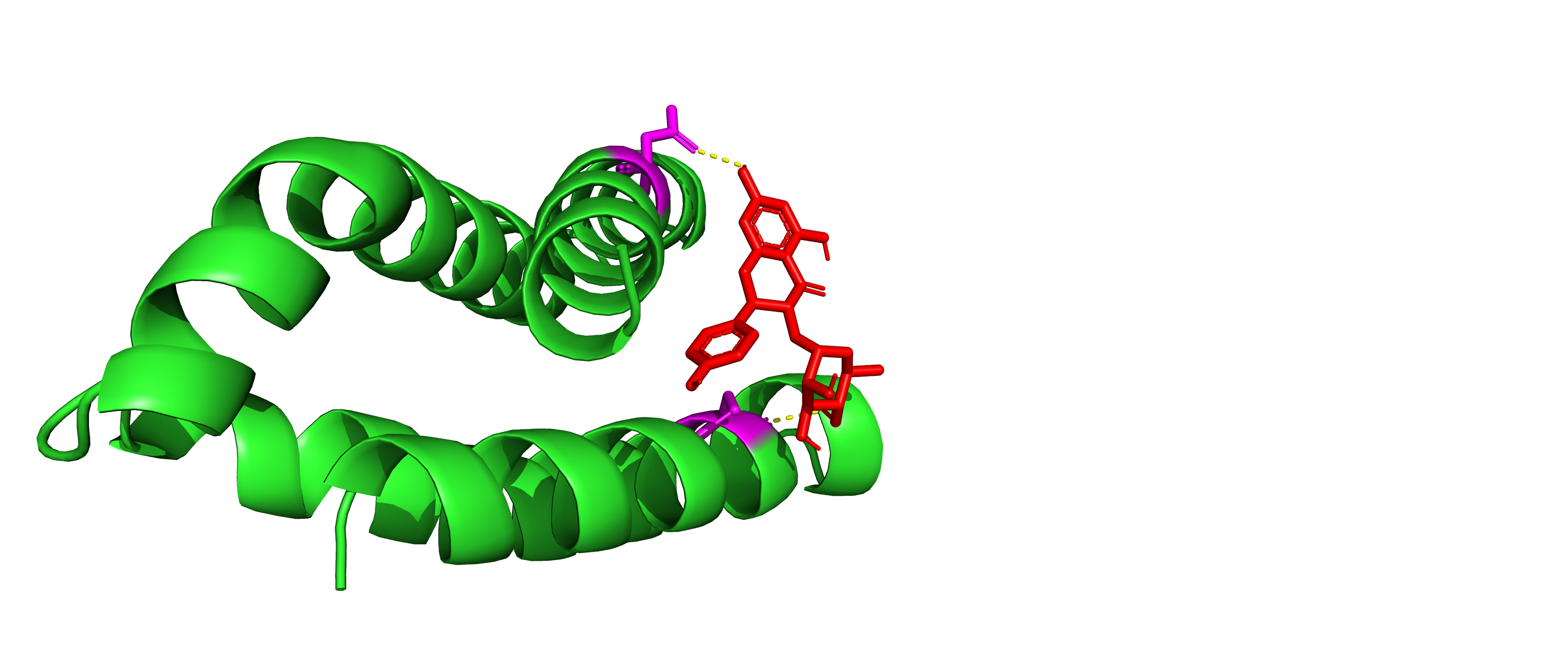

Supplement: Supplementary file 1 [file ijms-26-11446-s001.zip › MTOR/big.png]

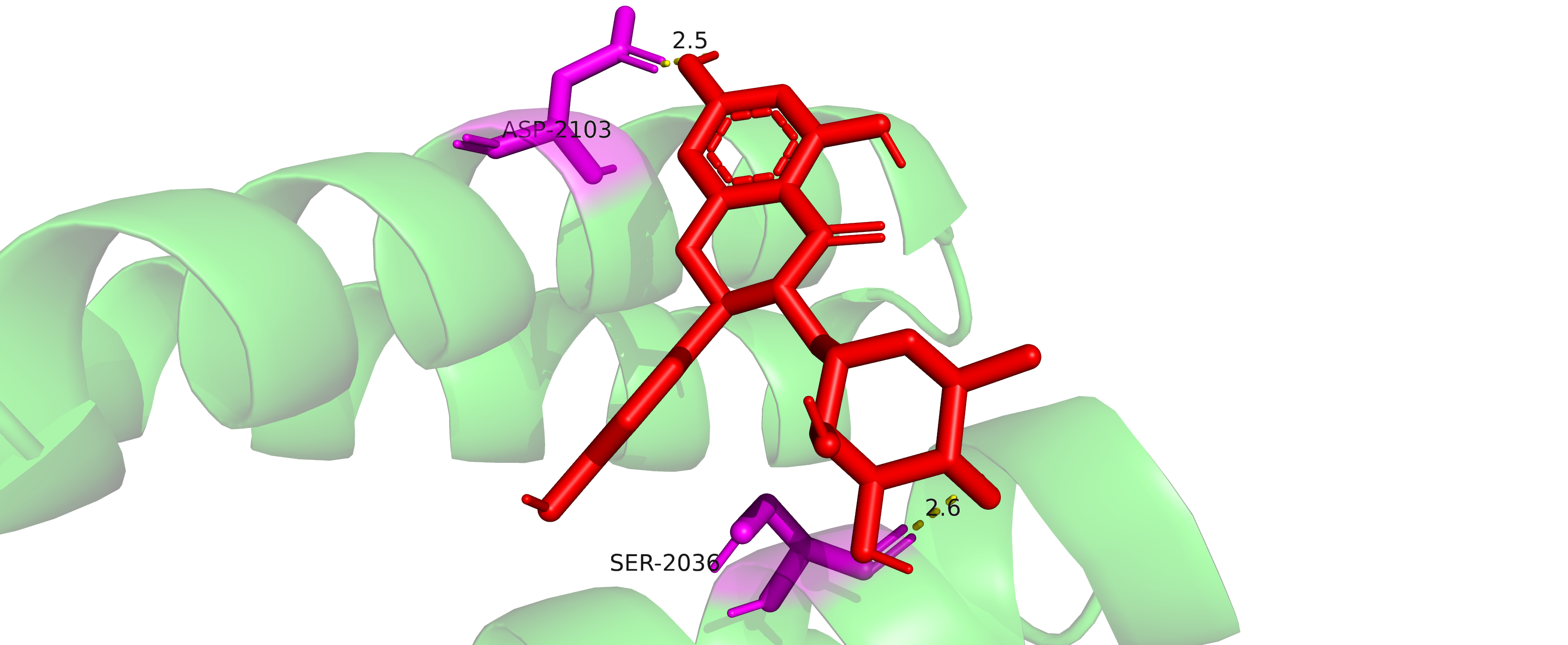

Supplement: Supplementary file 1 [file ijms-26-11446-s001.zip › MTOR/small.png]

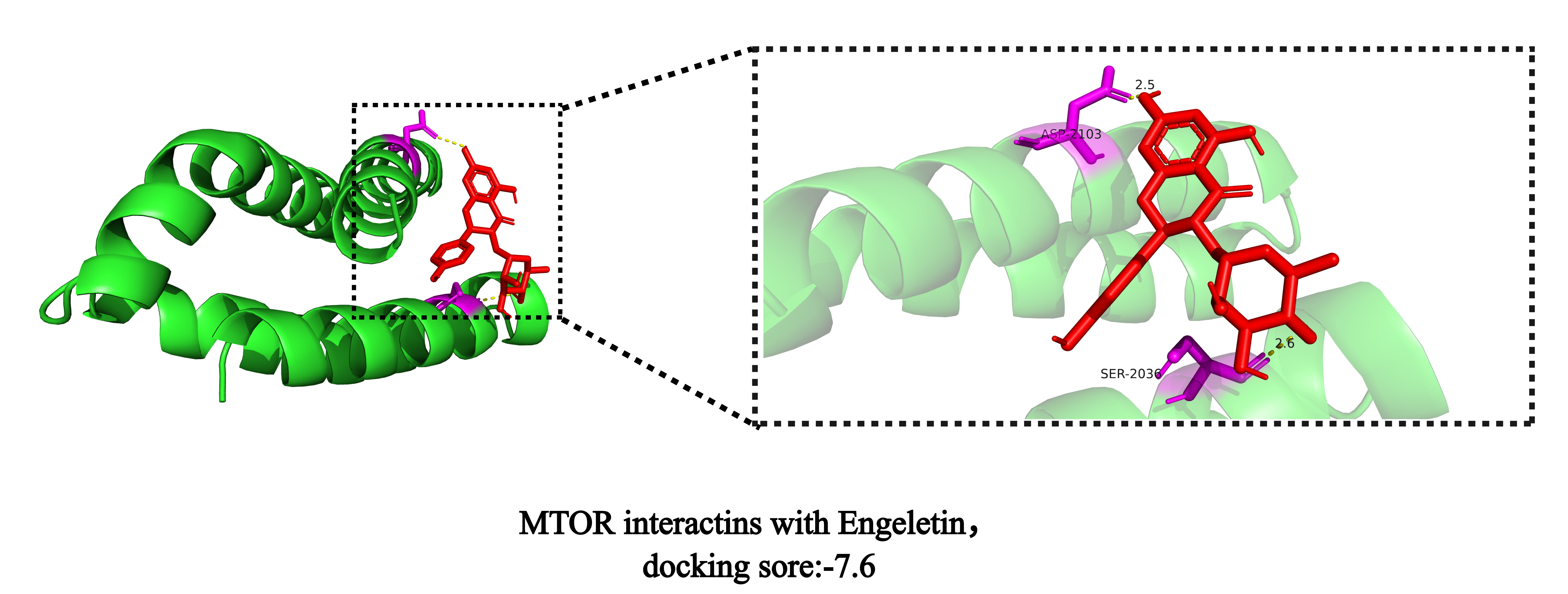

Supplement: Supplementary file 1 [file ijms-26-11446-s001.zip › MTOR/combination.png]

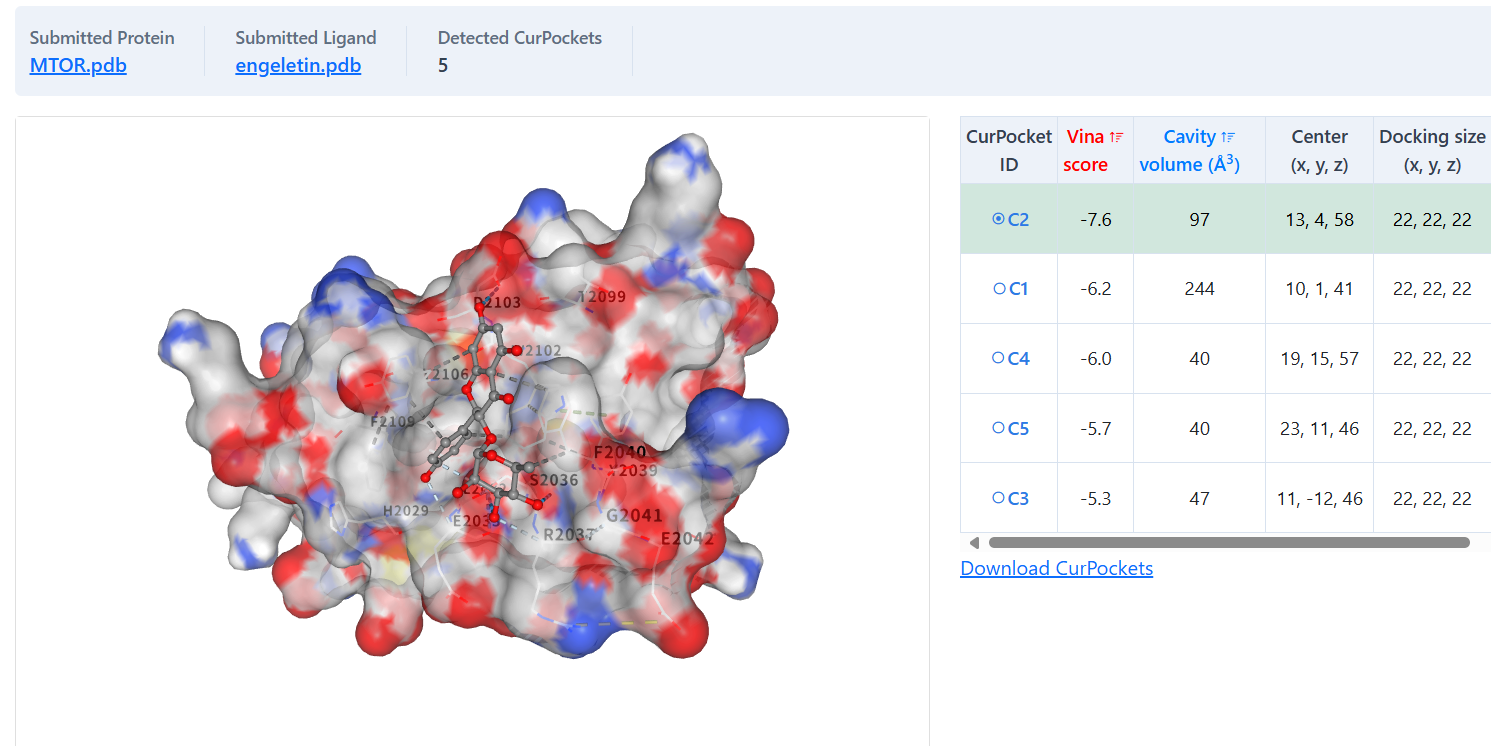

Supplement: Supplementary file 1 [file ijms-26-11446-s001.zip › MTOR/energy.png]

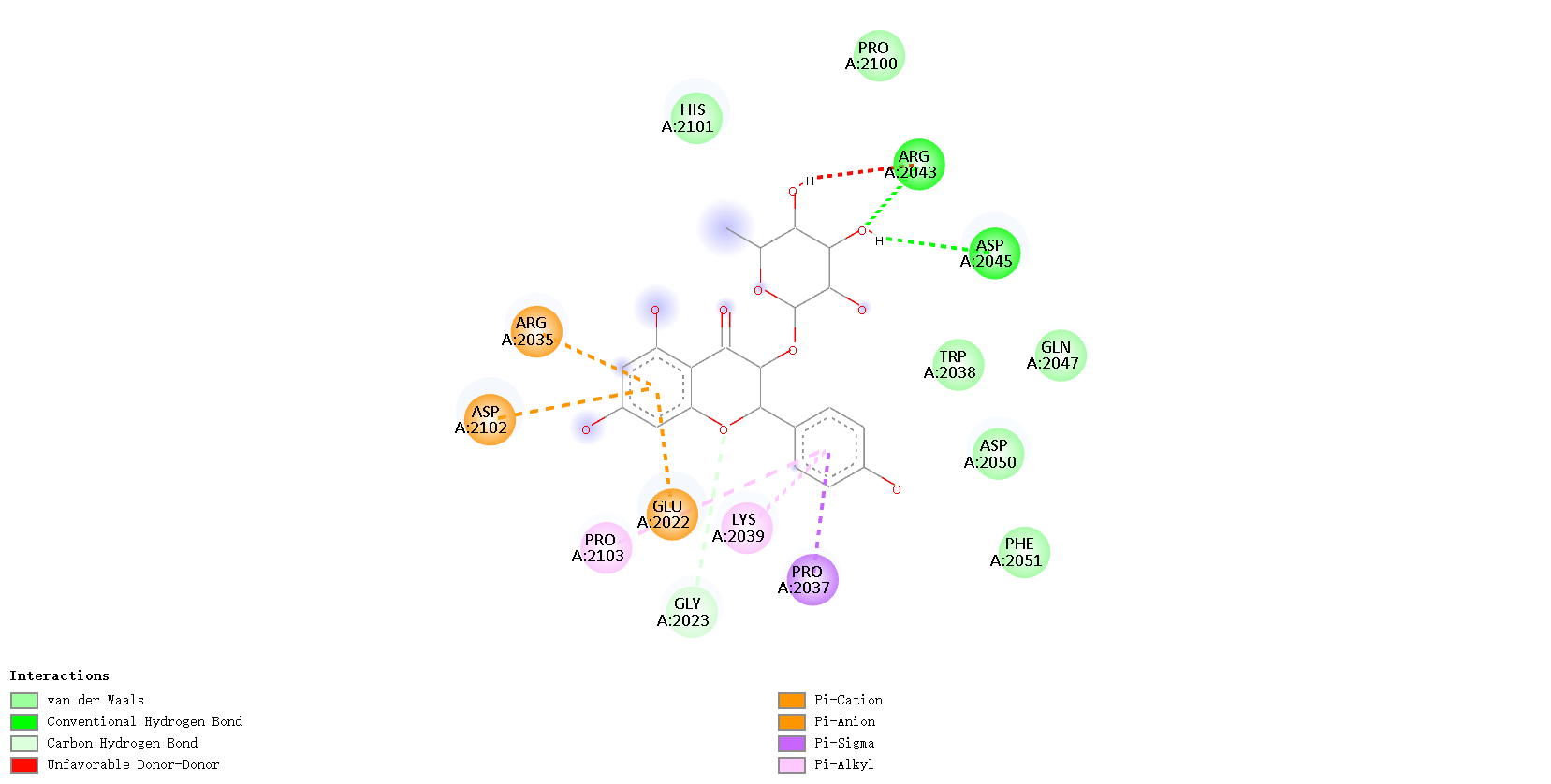

Supplement: Supplementary file 1 [file ijms-26-11446-s001.zip › NF-KB/2D.png]

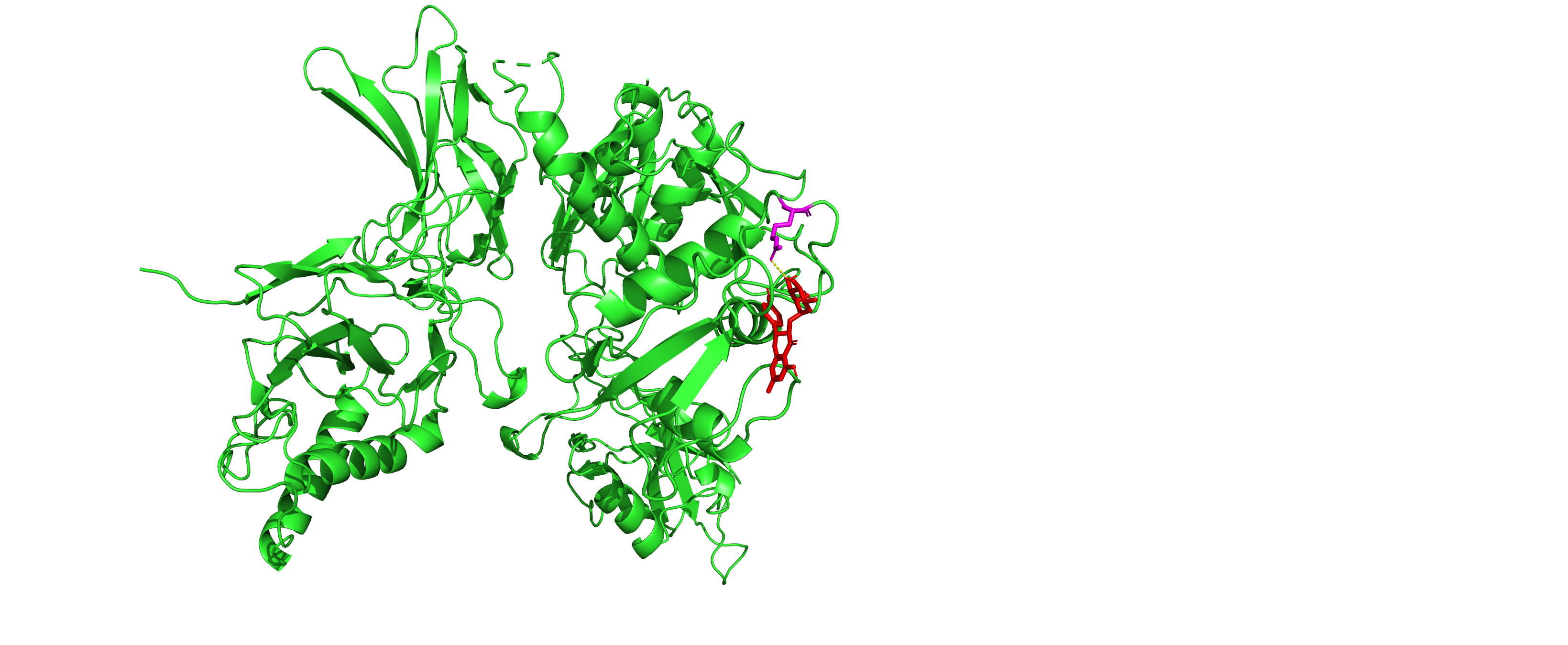

Supplement: Supplementary file 1 [file ijms-26-11446-s001.zip › NF-KB/big.png]

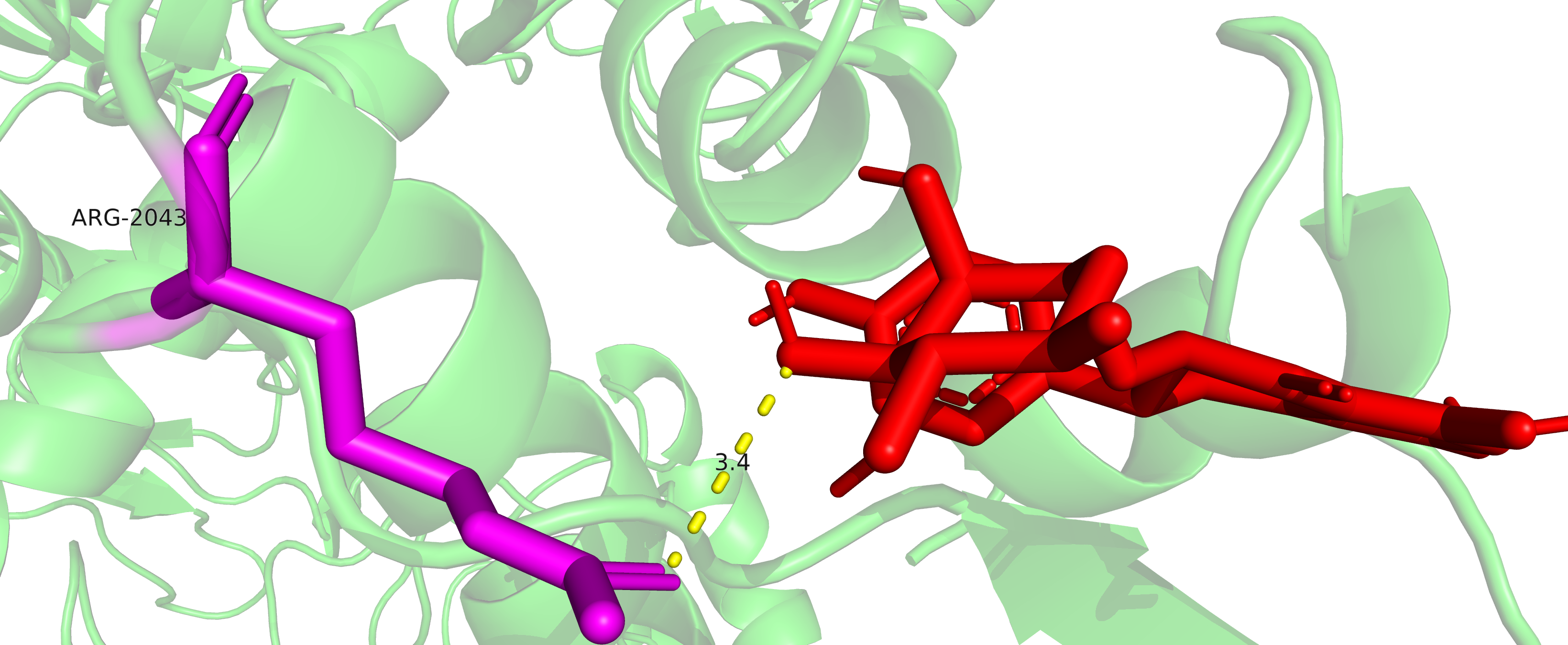

Supplement: Supplementary file 1 [file ijms-26-11446-s001.zip › NF-KB/small.png]

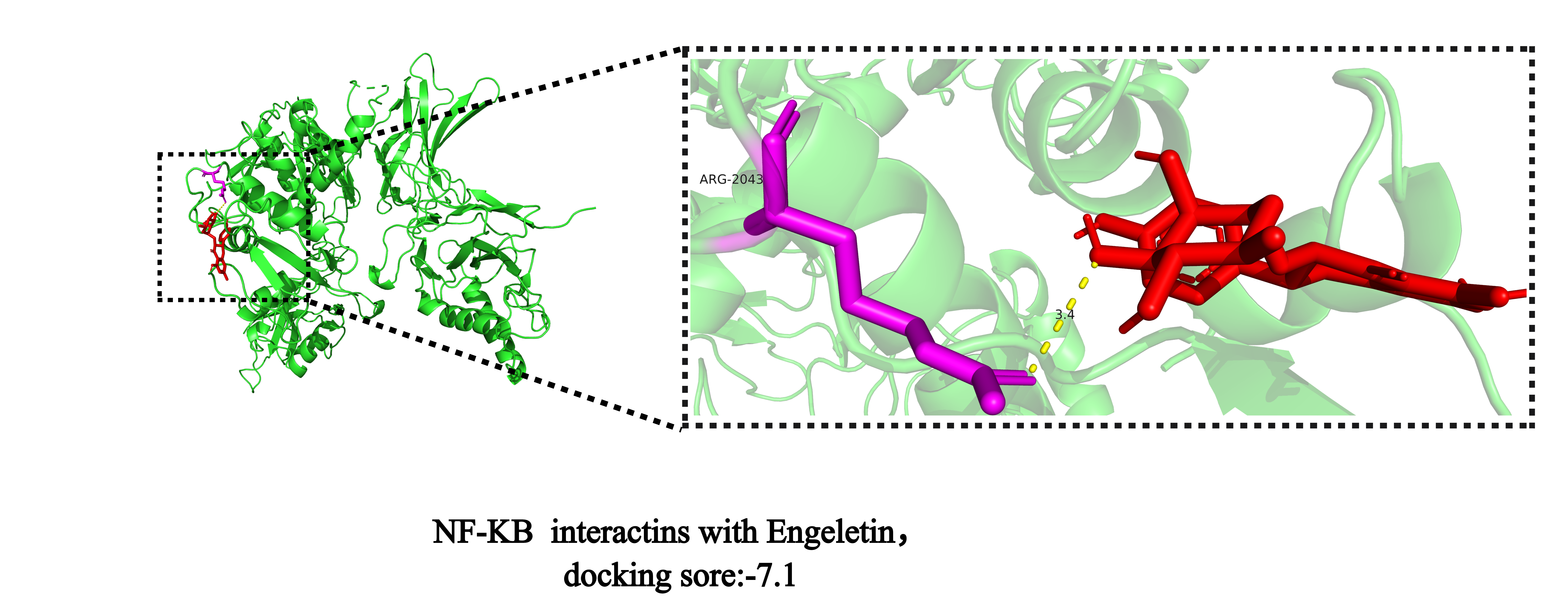

Supplement: Supplementary file 1 [file ijms-26-11446-s001.zip › NF-KB/combination.png]

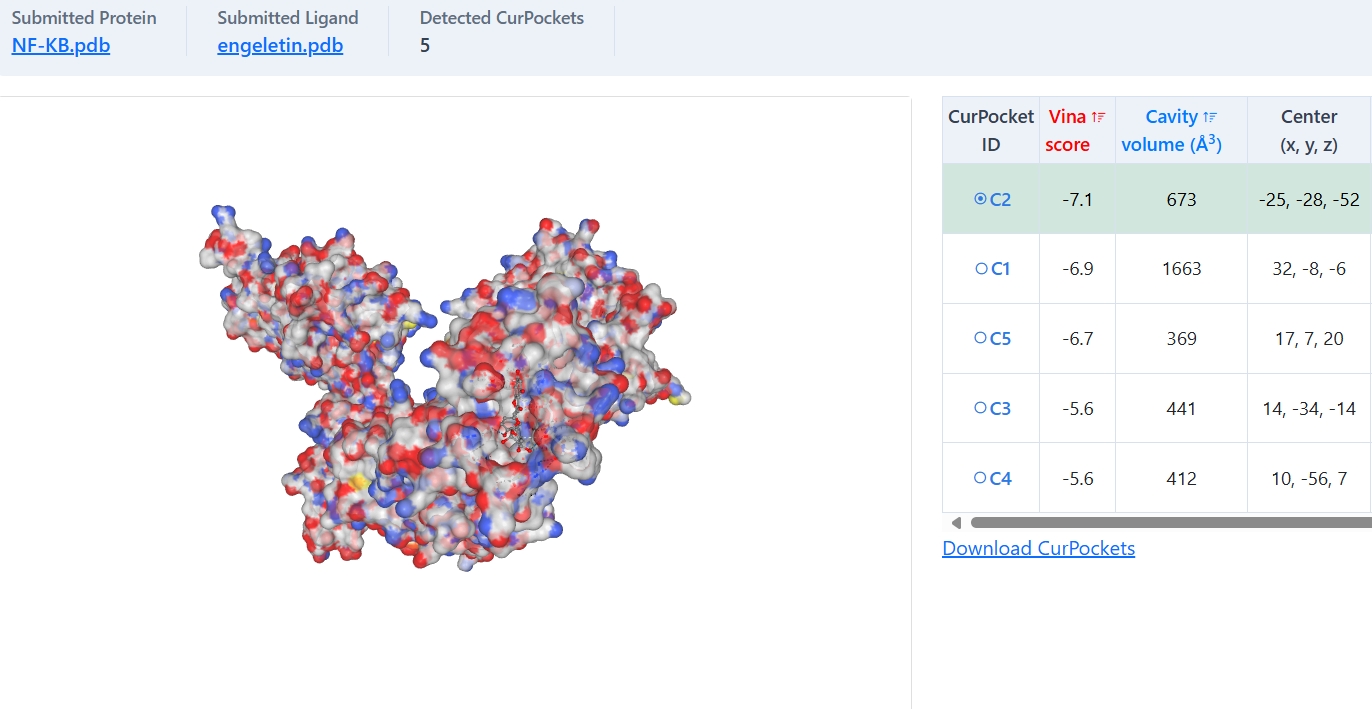

Supplement: Supplementary file 1 [file ijms-26-11446-s001.zip › NF-KB/energy.png]

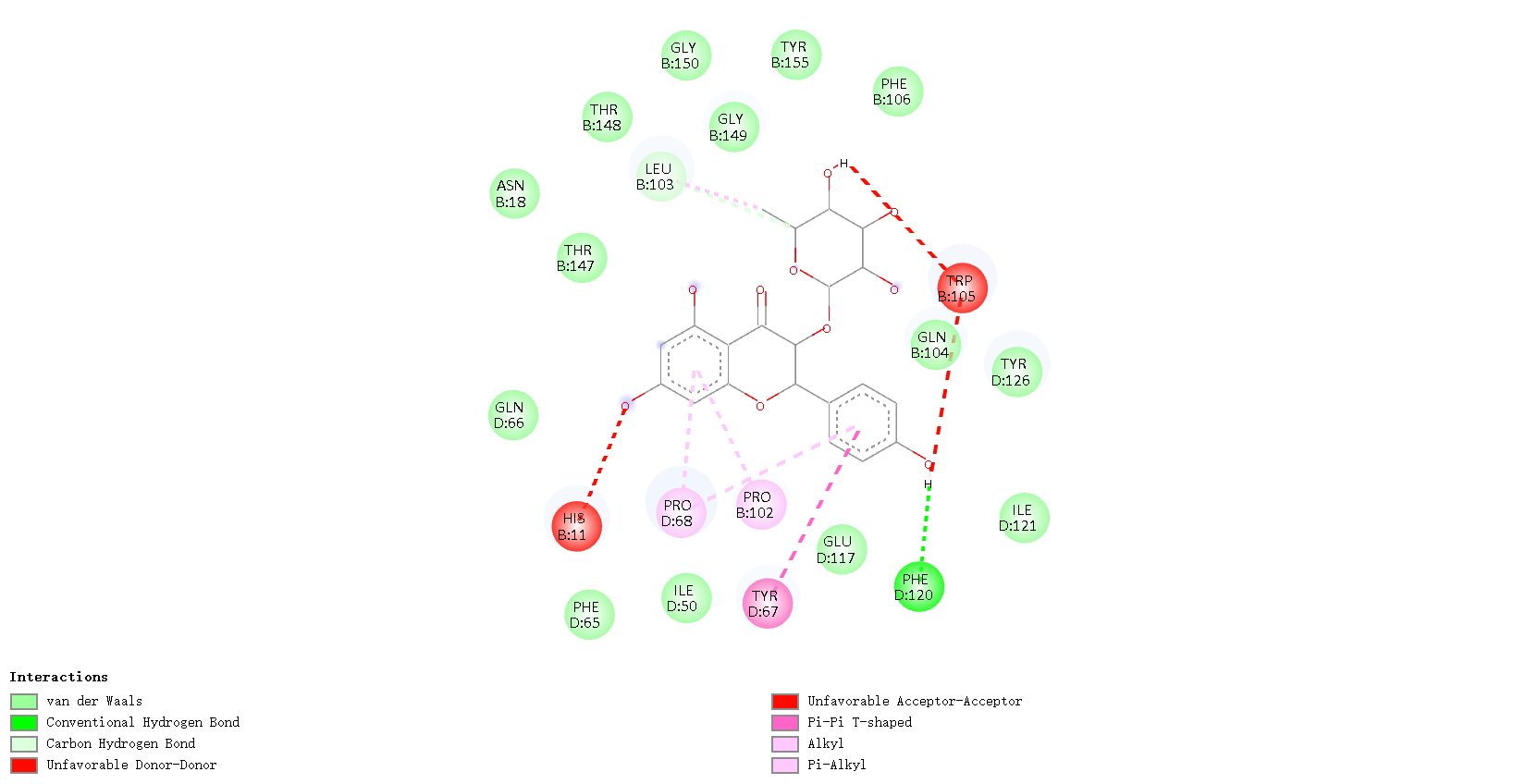

Supplement: Supplementary file 1 [file ijms-26-11446-s001.zip › NQ01/2d.png]

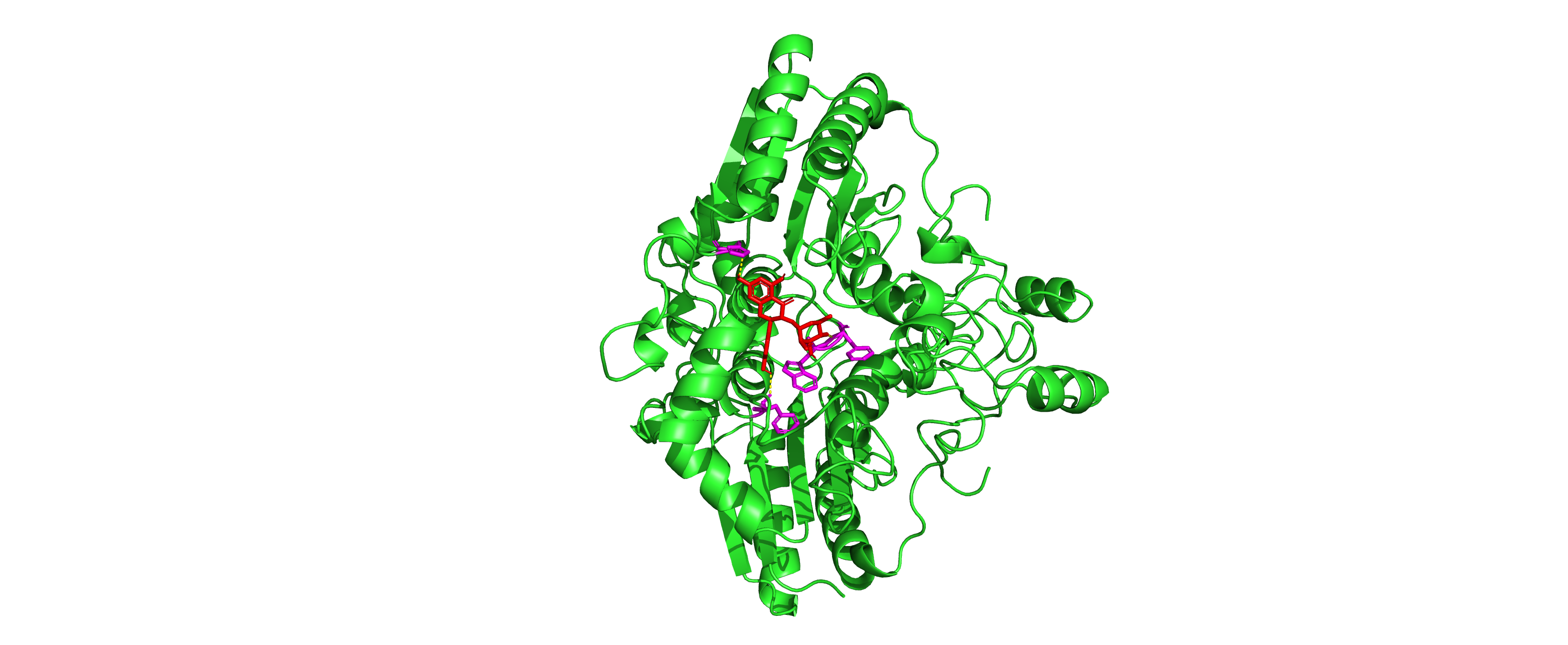

Supplement: Supplementary file 1 [file ijms-26-11446-s001.zip › NQ01/big.png]

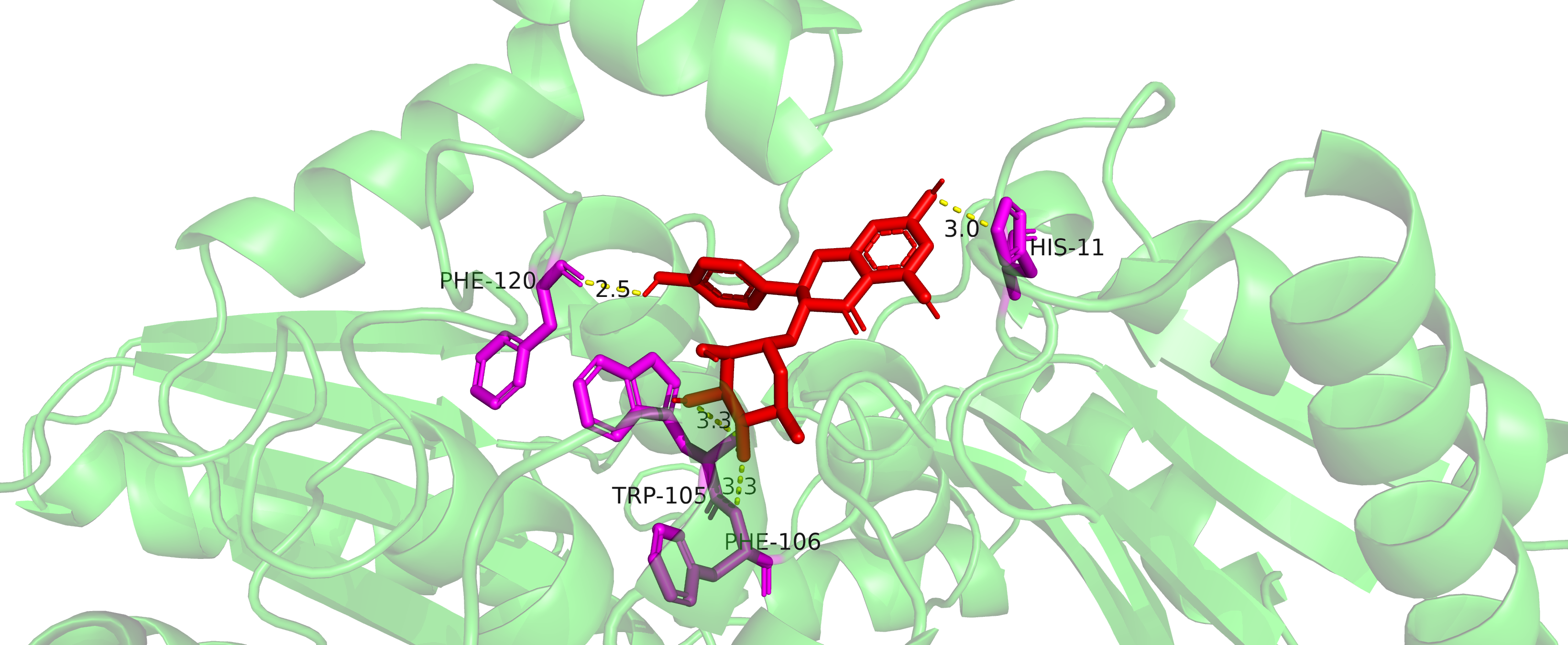

Supplement: Supplementary file 1 [file ijms-26-11446-s001.zip › NQ01/small.png]

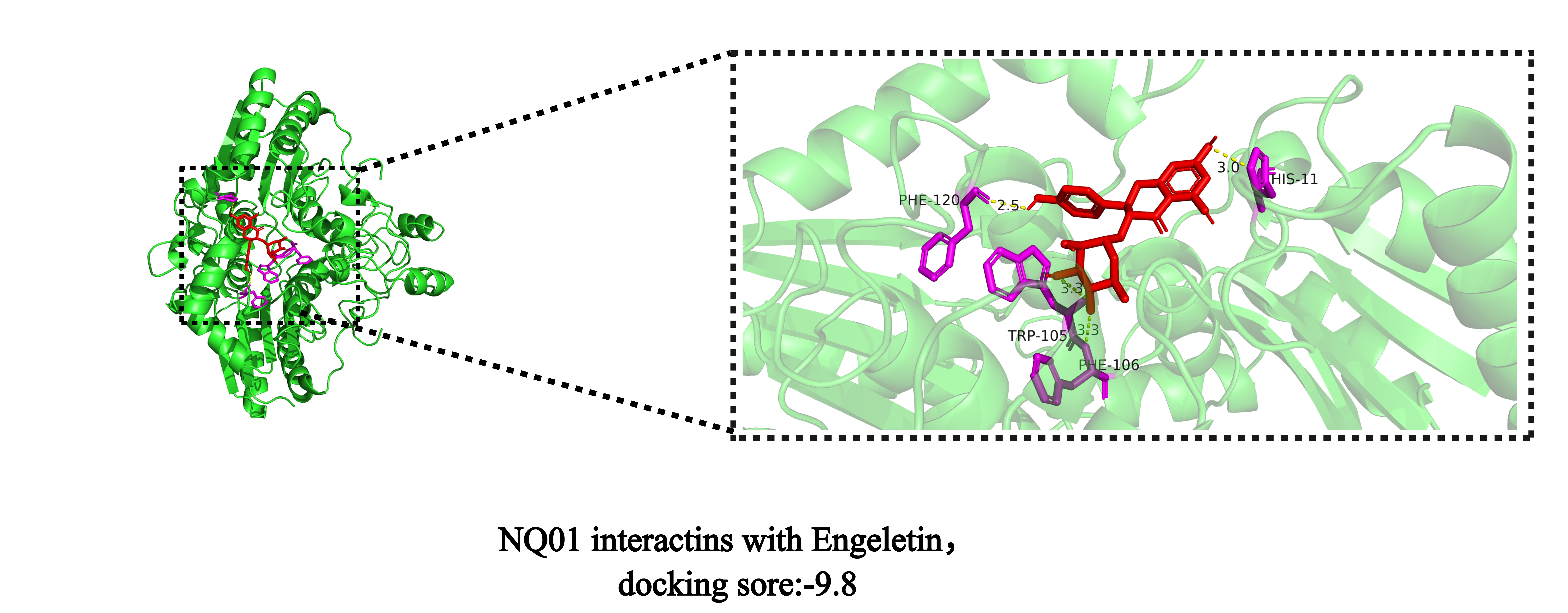

Supplement: Supplementary file 1 [file ijms-26-11446-s001.zip › NQ01/combination.png]

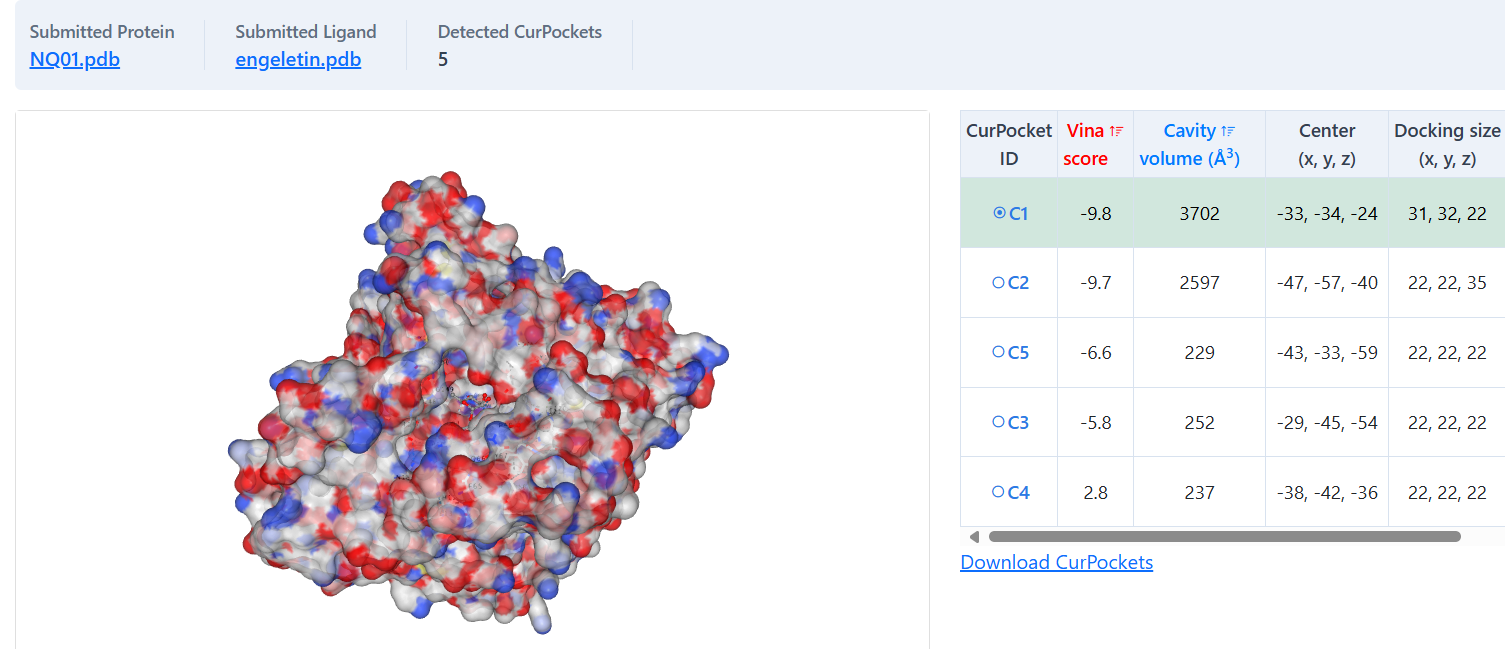

Supplement: Supplementary file 1 [file ijms-26-11446-s001.zip › NQ01/energy.png]

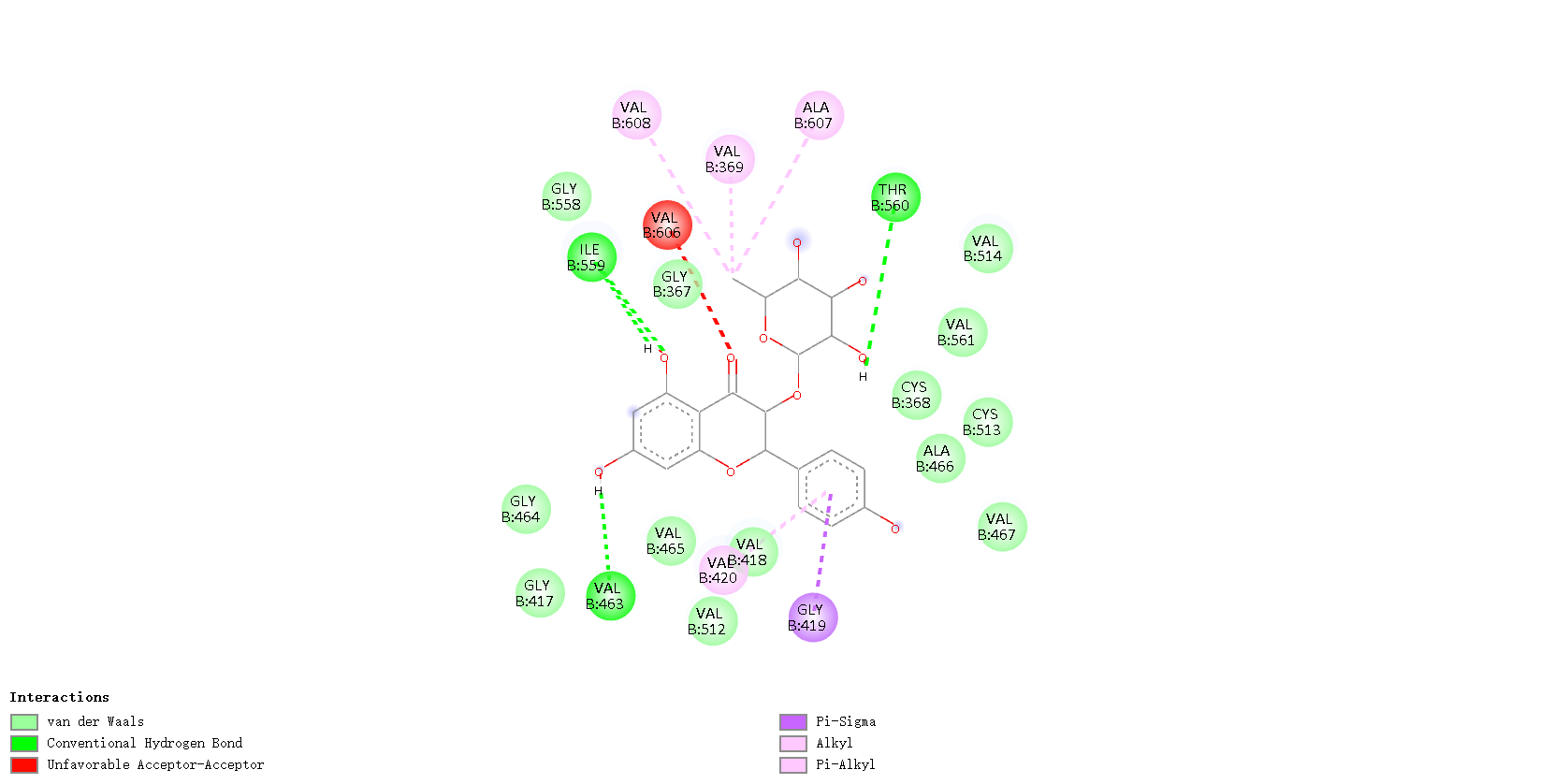

Supplement: Supplementary file 1 [file ijms-26-11446-s001.zip › nrf2/2D.png]

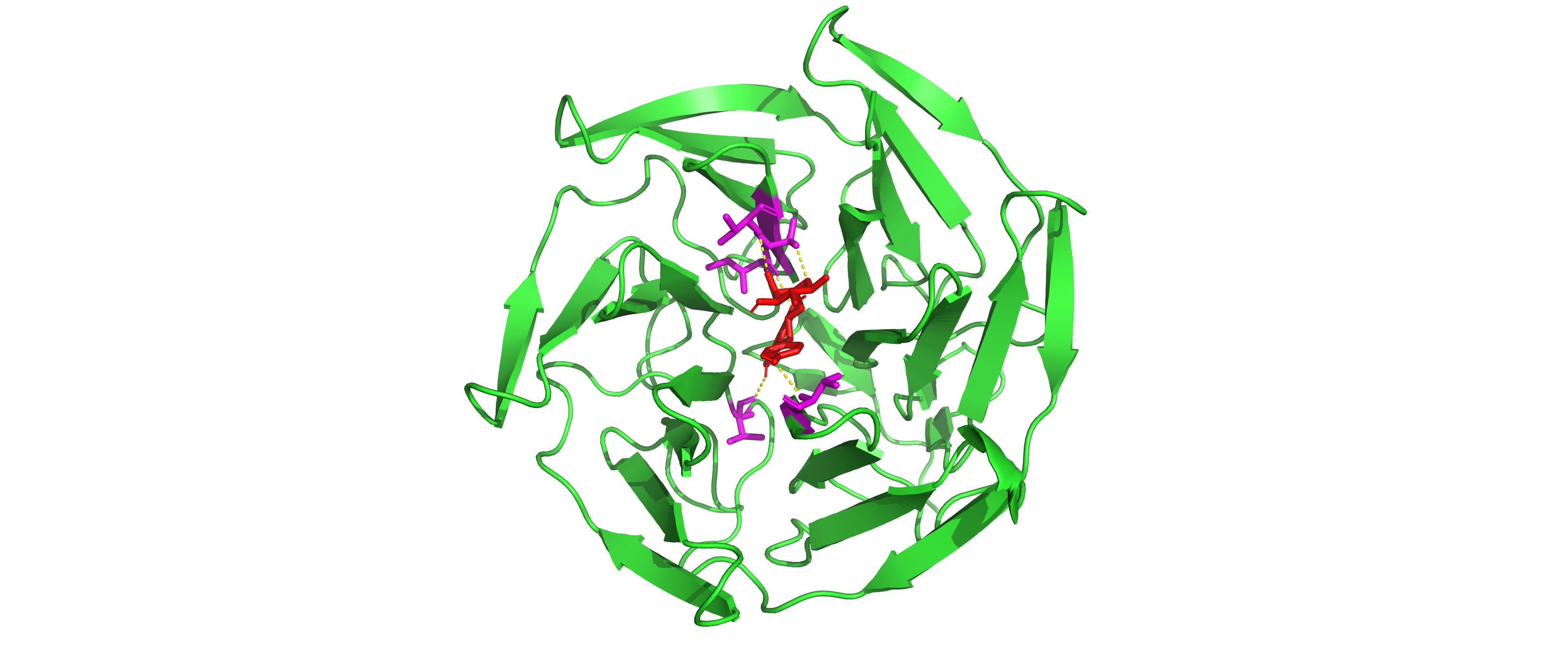

Supplement: Supplementary file 1 [file ijms-26-11446-s001.zip › nrf2/big.png]

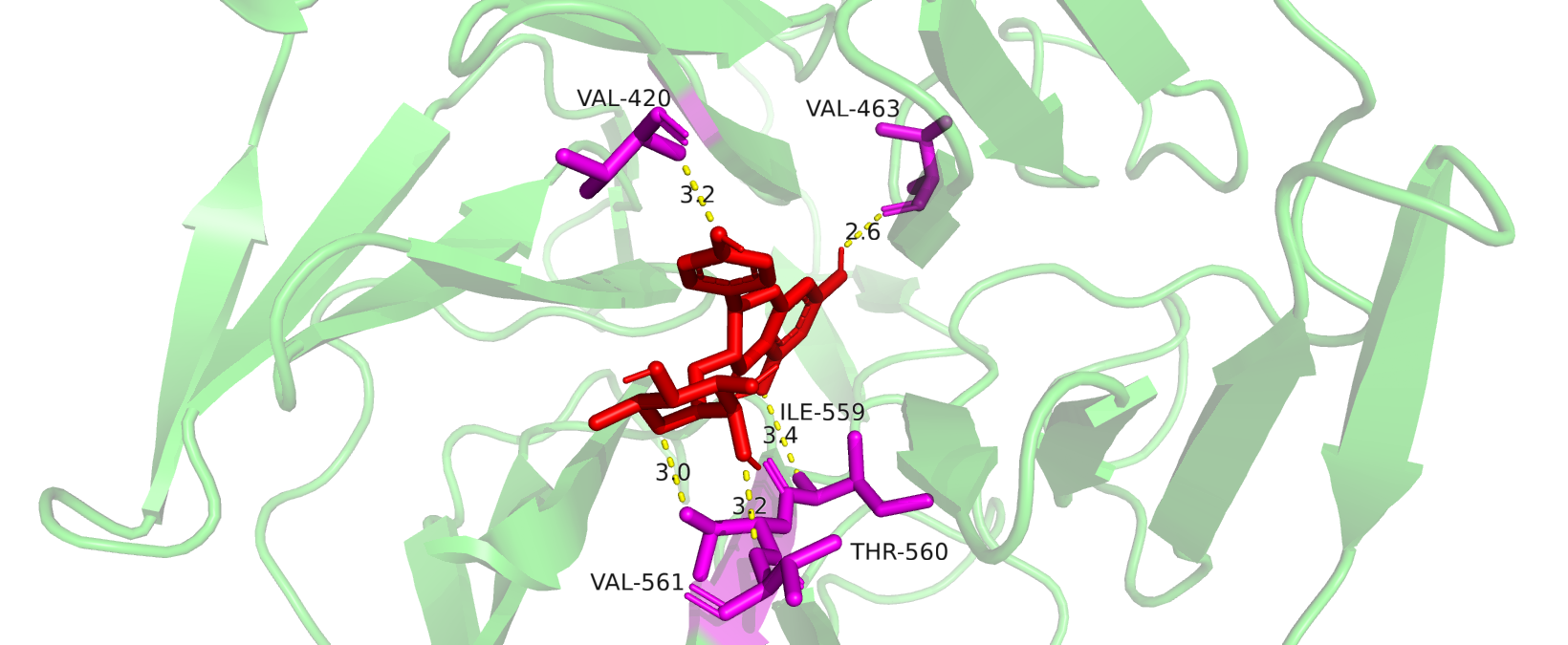

Supplement: Supplementary file 1 [file ijms-26-11446-s001.zip › nrf2/small.png]

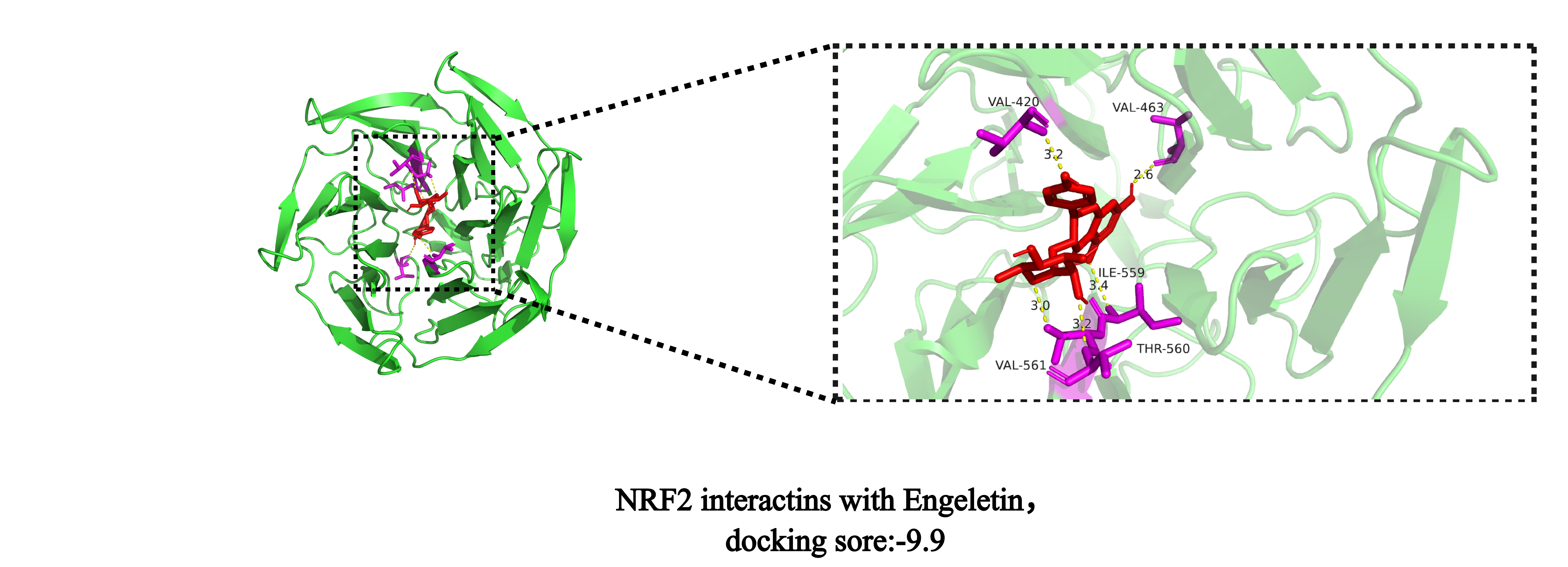

Supplement: Supplementary file 1 [file ijms-26-11446-s001.zip › nrf2/combination.png]

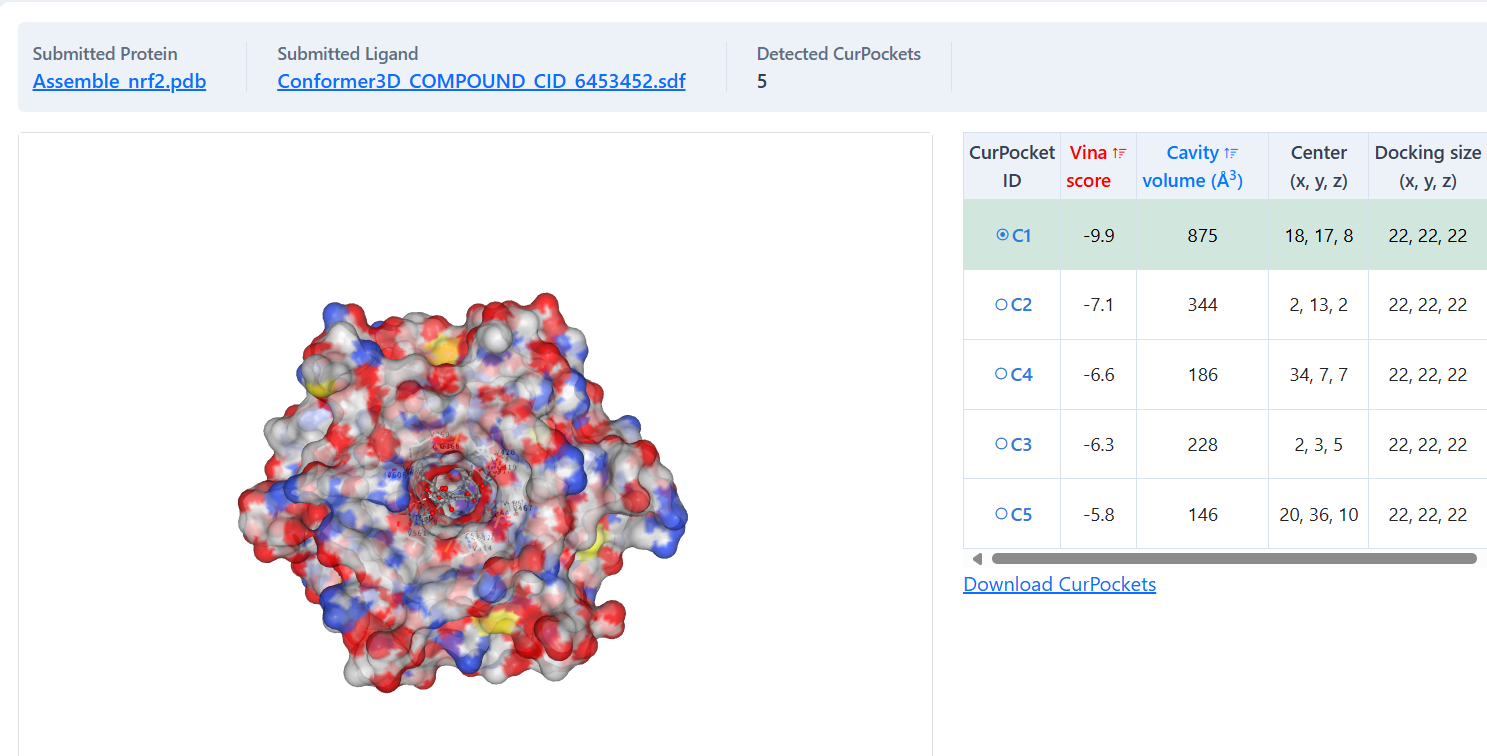

Supplement: Supplementary file 1 [file ijms-26-11446-s001.zip › nrf2/energy.png]

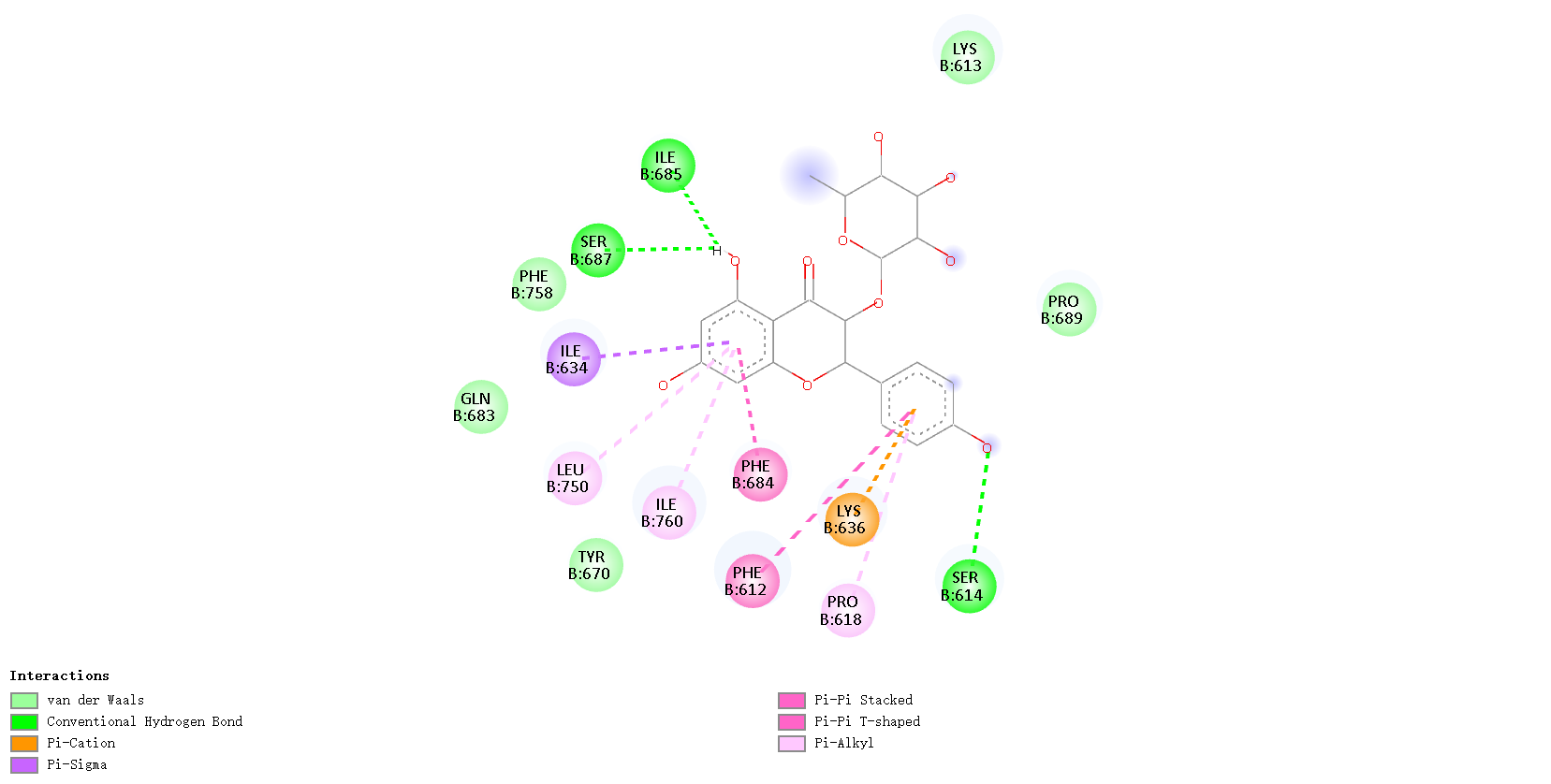

Supplement: Supplementary file 1 [file ijms-26-11446-s001.zip › PI3K/2D.png]

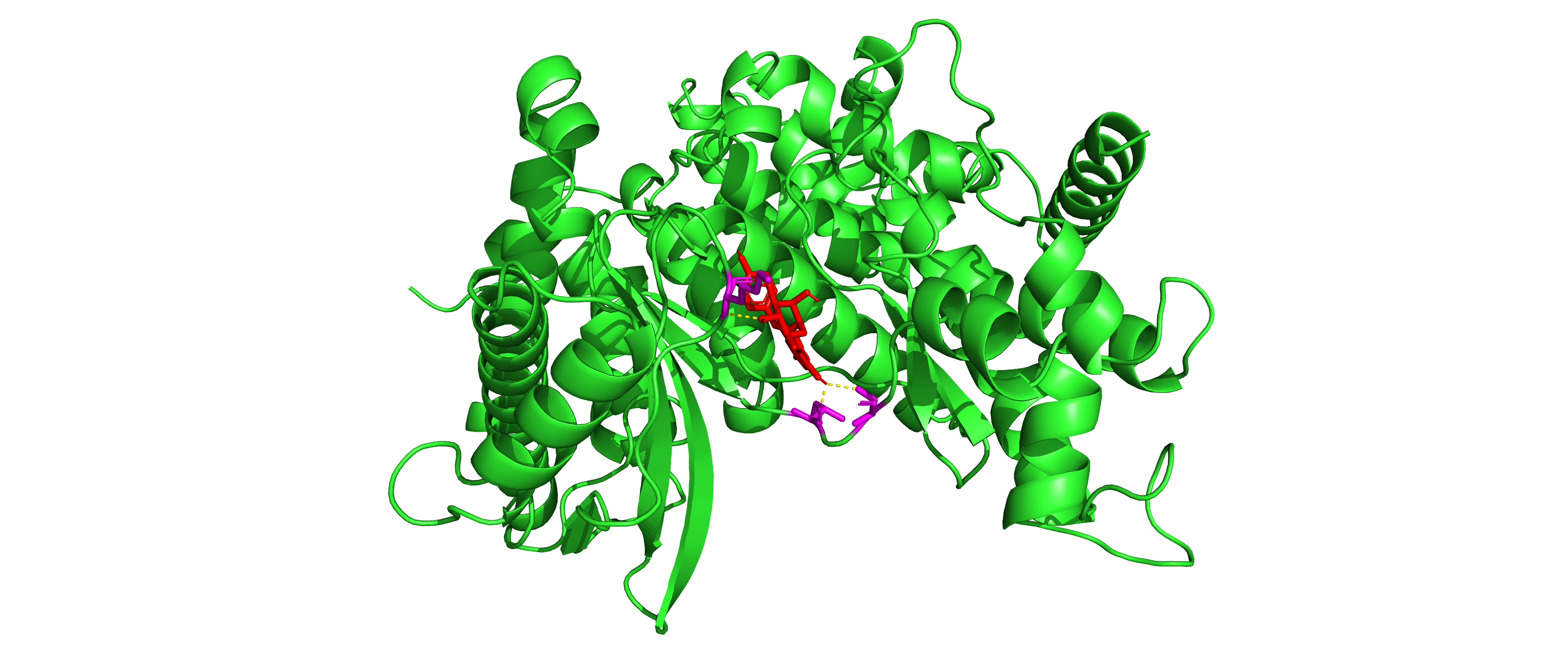

Supplement: Supplementary file 1 [file ijms-26-11446-s001.zip › PI3K/big.png]

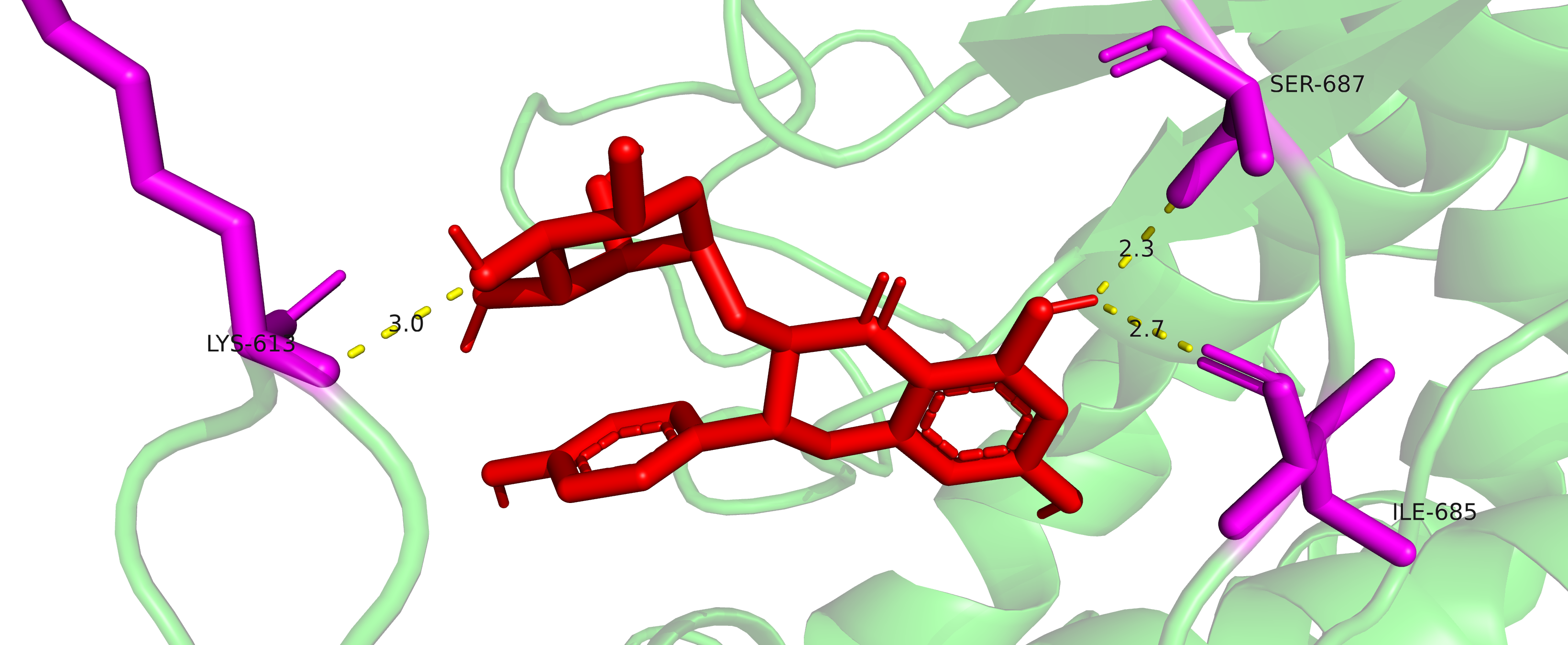

Supplement: Supplementary file 1 [file ijms-26-11446-s001.zip › PI3K/small.png]

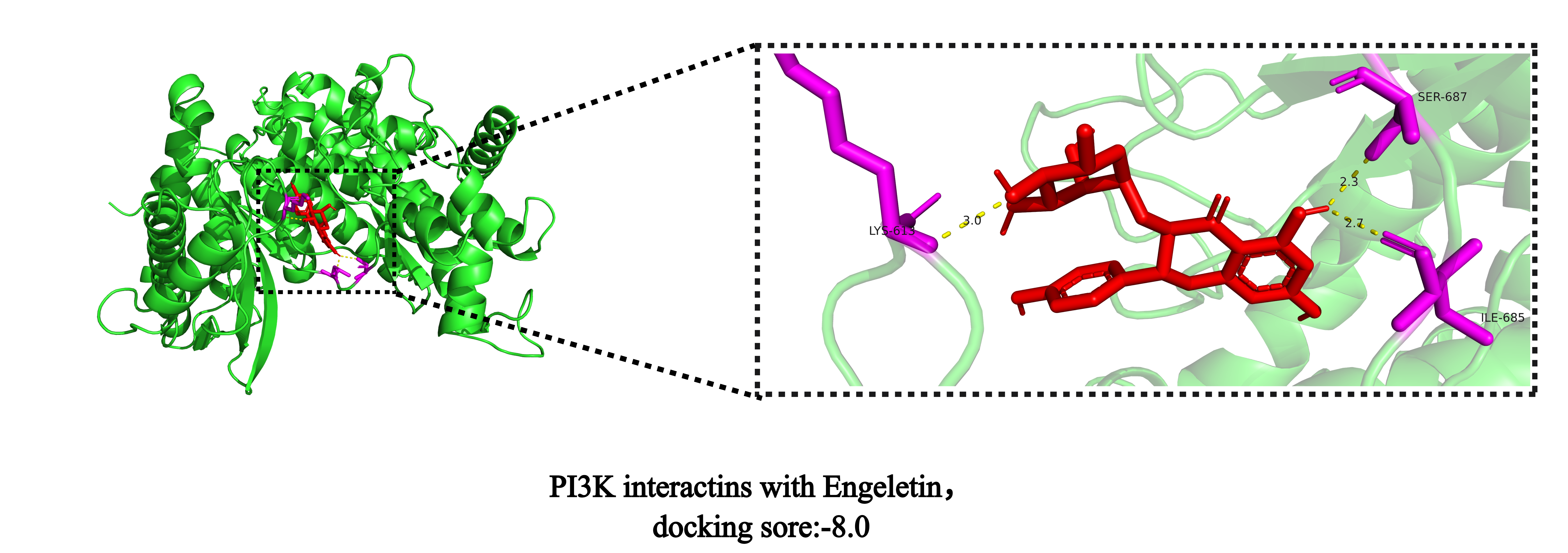

Supplement: Supplementary file 1 [file ijms-26-11446-s001.zip › PI3K/combination.png]

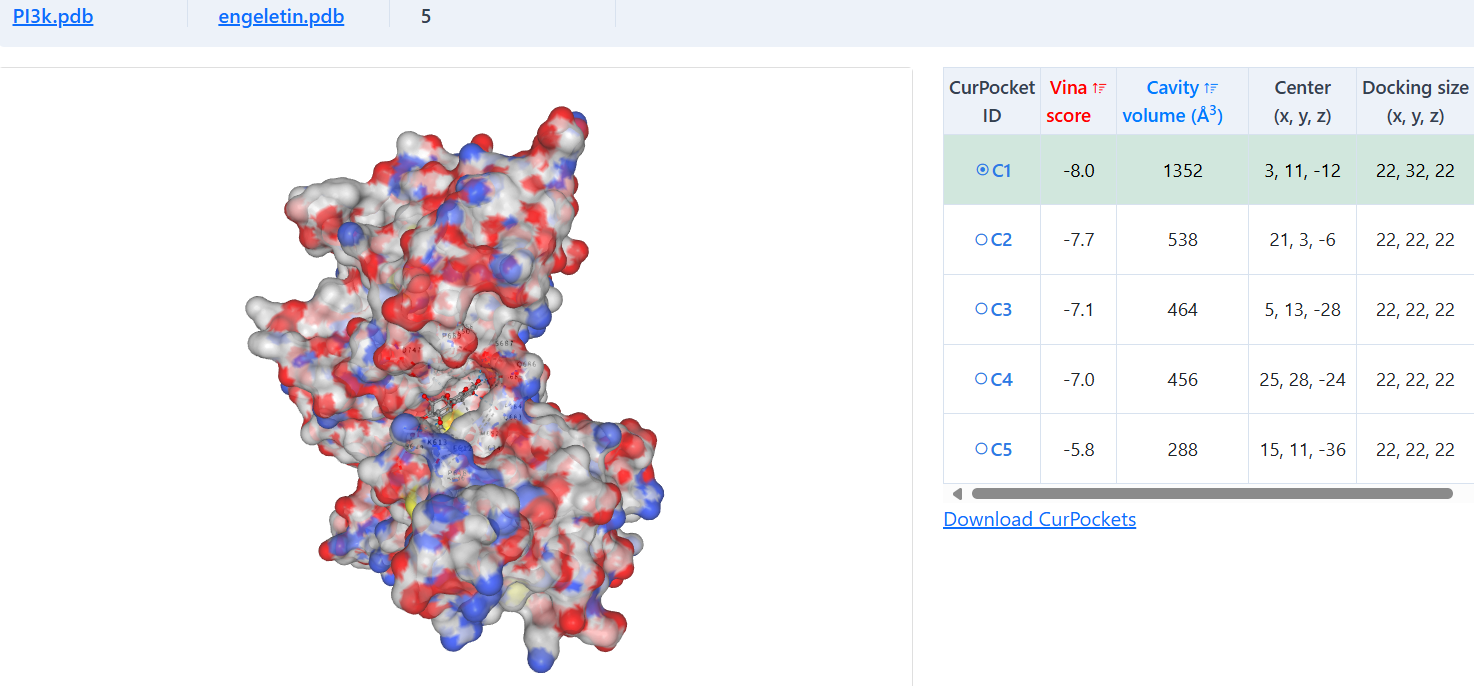

Supplement: Supplementary file 1 [file ijms-26-11446-s001.zip › PI3K/energy.png]

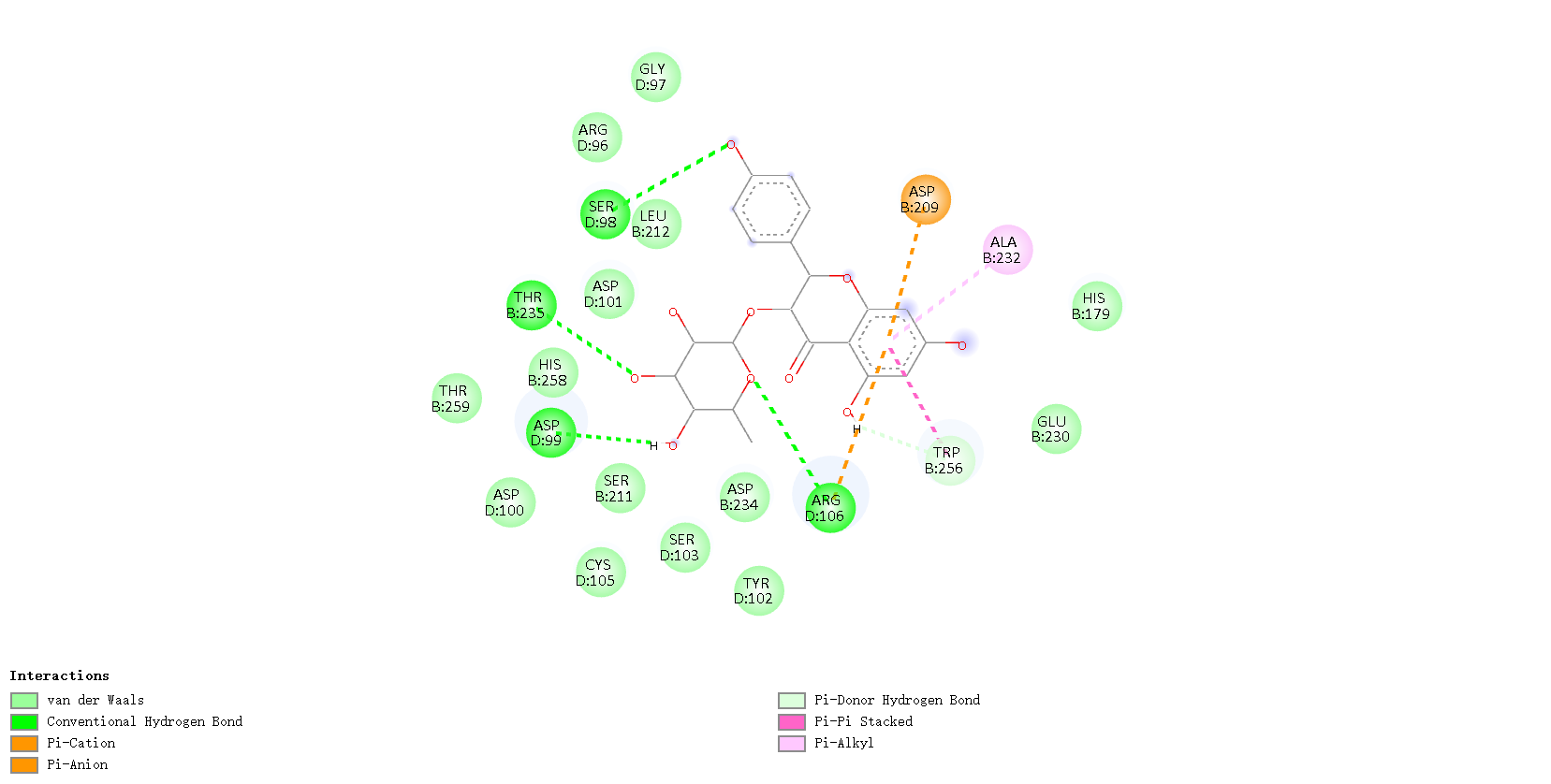

Supplement: Supplementary file 1 [file ijms-26-11446-s001.zip › TLR4/2D.png]

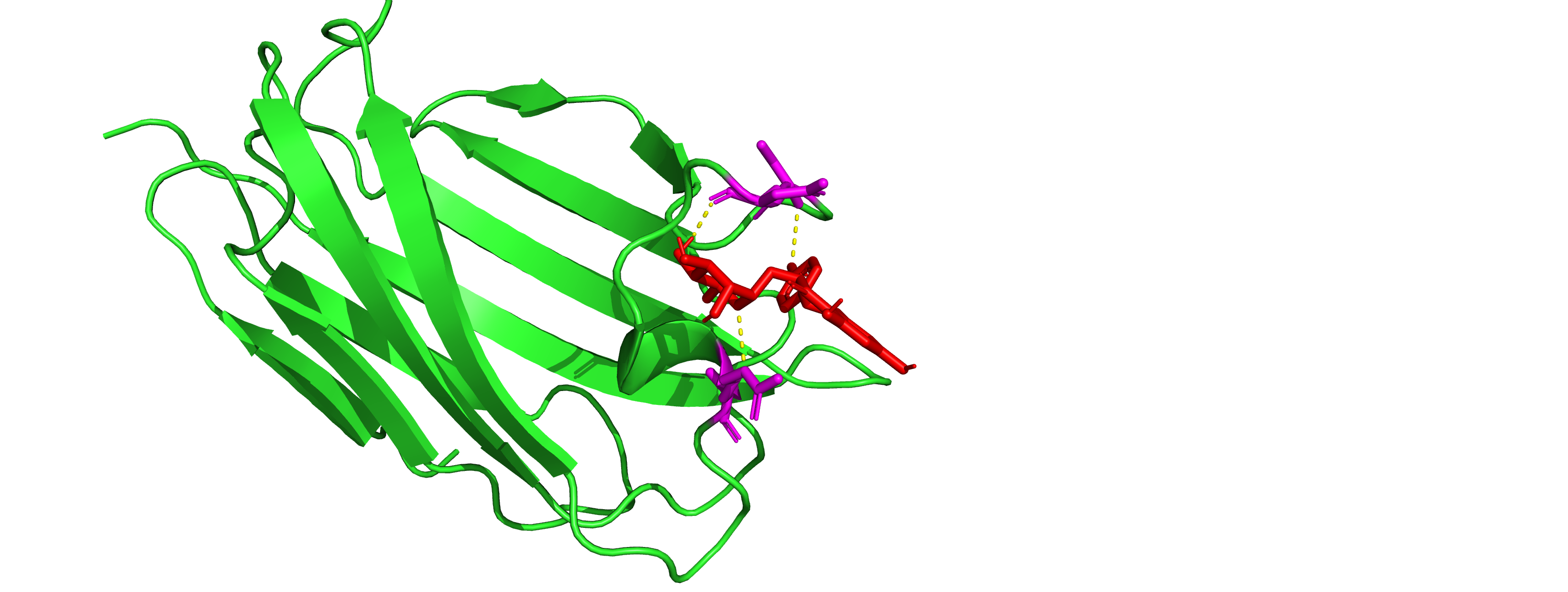

Supplement: Supplementary file 1 [file ijms-26-11446-s001.zip › TLR4/big.png]

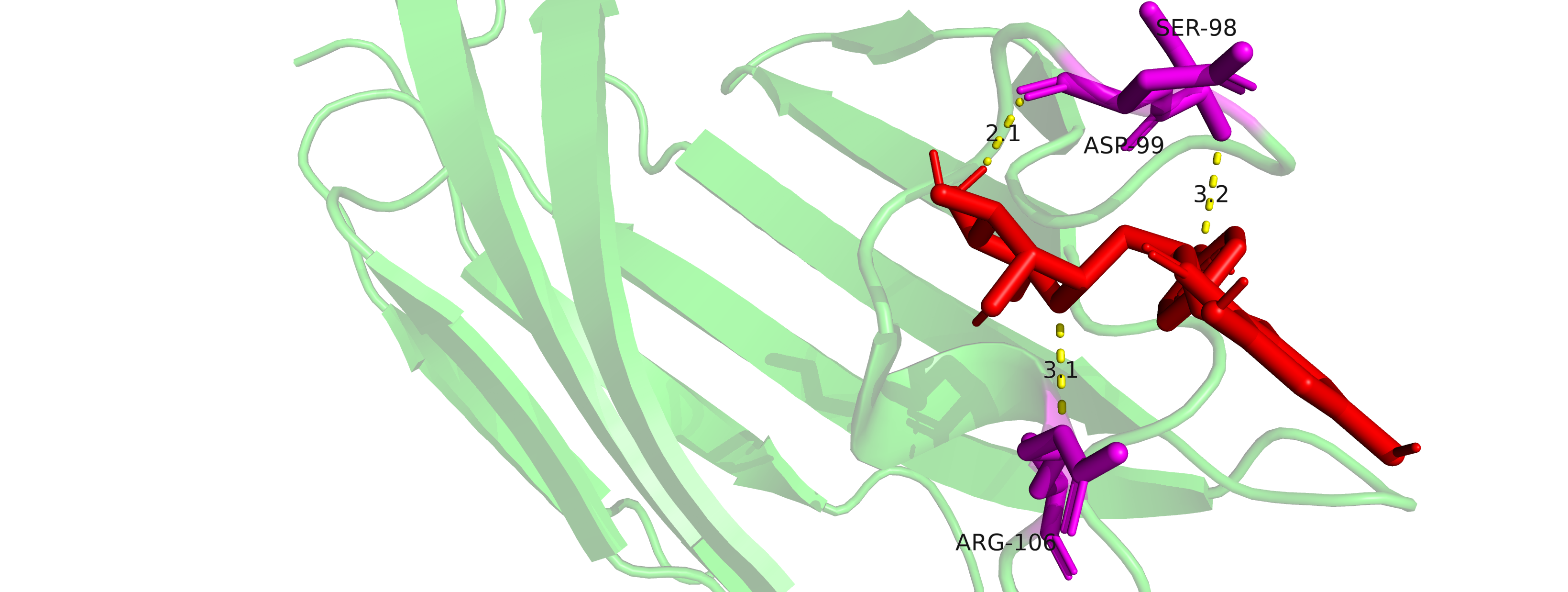

Supplement: Supplementary file 1 [file ijms-26-11446-s001.zip › TLR4/small.png]

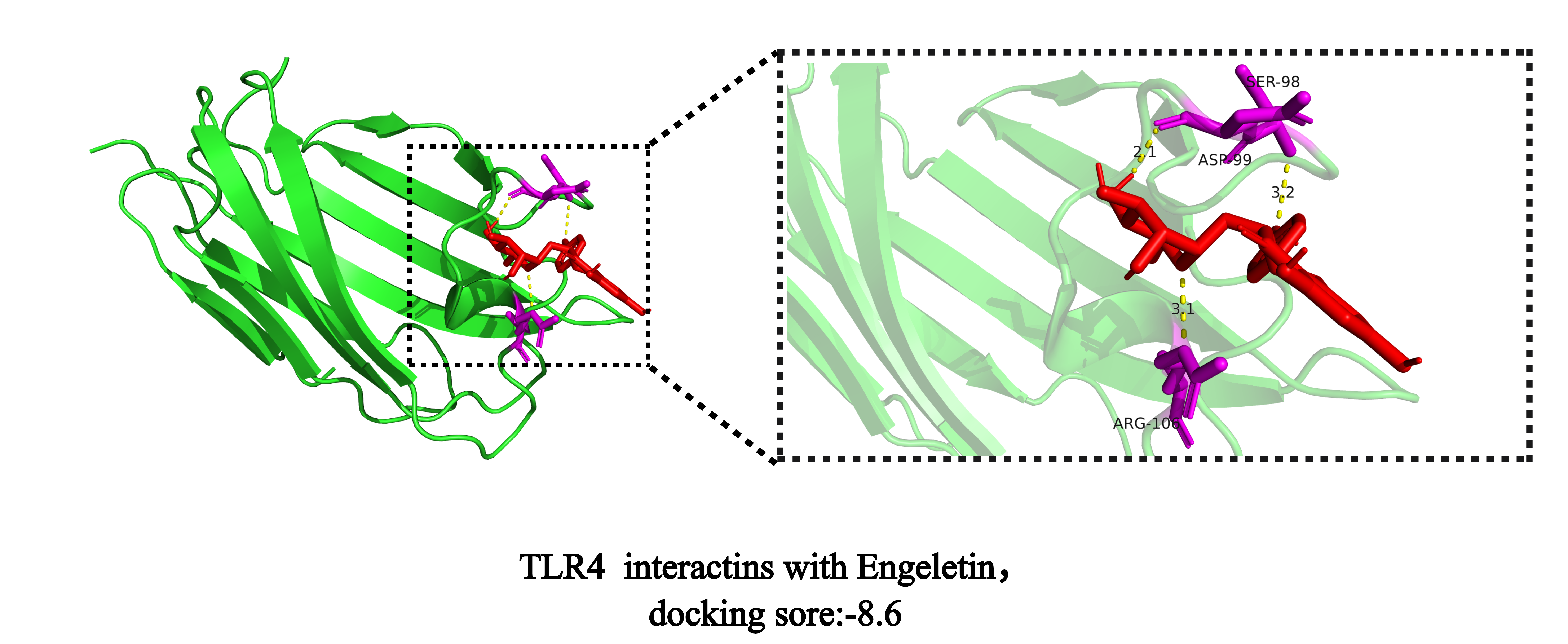

Supplement: Supplementary file 1 [file ijms-26-11446-s001.zip › TLR4/combination.png]

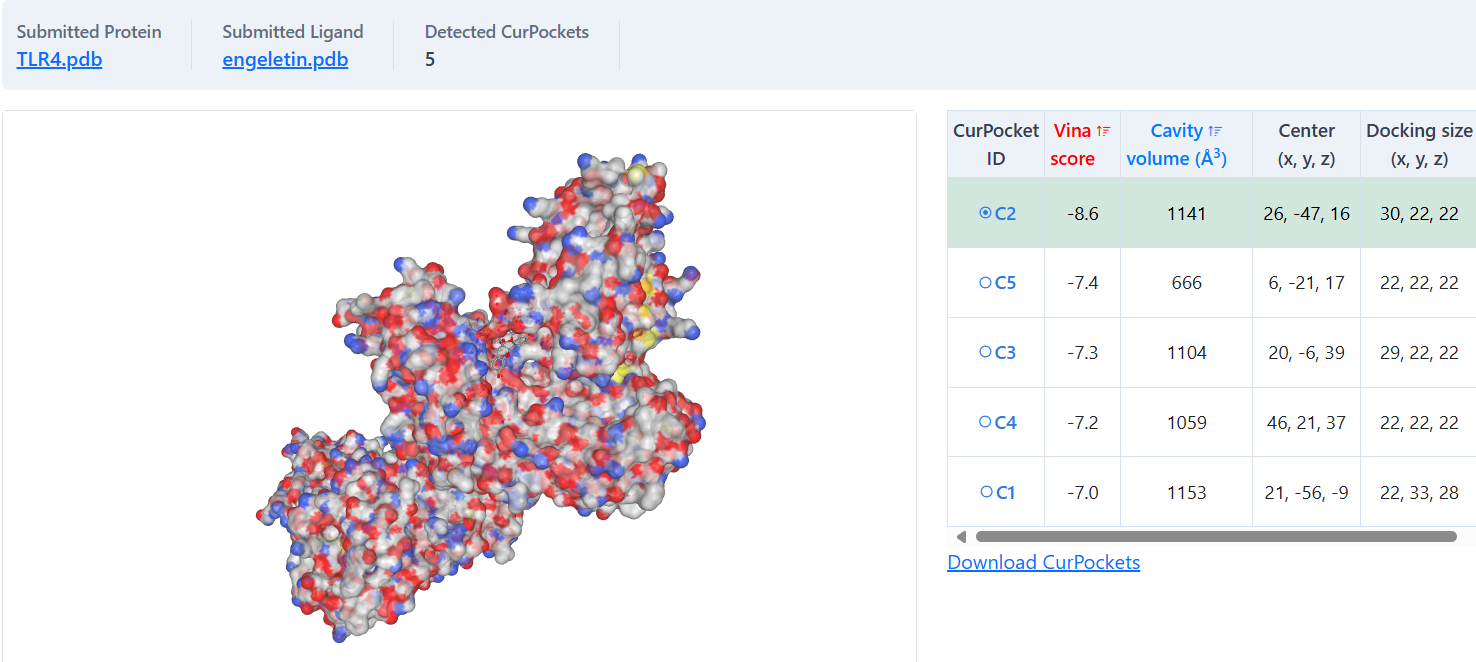

Supplement: Supplementary file 1 [file ijms-26-11446-s001.zip › TLR4/energy.png]
